# Improving Polymeric Structures with Hirshfeld Atom Refinement: A Study on MOFs and COFs

**DOI:** 10.1021/acsmaterialsau.5c00076

**Published:** 2025-07-31

**Authors:** Magdalena Woińska, Anna Makal, Paweł Grzymski-Ostręga, Michal L. Chodkiewicz, Krzysztof Wozniak

**Affiliations:** Faculty of Chemistry, 49605 University of Warsaw, Pasteura 1, 02-093 Warsaw, Poland

**Keywords:** Hirshfeld atom refinement, metal organic frameworks, covalent organic frameworks, polymeric crystal structures, hydrogen atoms

## Abstract

Many metal–organic frameworks (MOFs) and covalent organic frameworks (COFs) can form crystals amenable to single-crystal X-ray diffraction analysis. It makes them suitable for Hirshfeld atom refinement (HAR) which has a well-established advantage over the Independent Atom Model in terms of the determination of hydrogen atom positions in the case of molecular crystals. However, up until now, the application of HAR to crystals of polymeric compounds such as MOFs and COFs has not been thoroughly investigated. This study of X-ray data sets collected for 20 MOFs, COFs and other coordination polymers is designed to provide an extensive assessment of two different implementations of HAR with respect to hydrogen positions and refinement statistics, given varying data quality.

## Graphical abstract

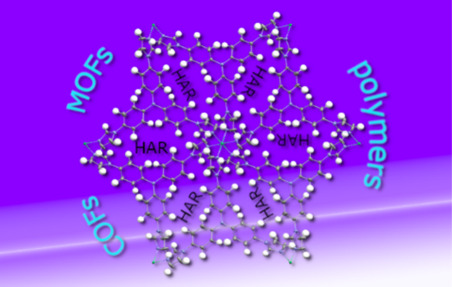

Metal–organic frameworks (MOFs) and covalent organic frameworks (COFs) are pivotal materials in modern science due to their extraordinary porosity, tunable structures, and diverse functionalities. MOFs, composed of metal nodes linked by organic ligands, and COFs, made of covalently bonded organic units, exhibit high surface areas, making them excellent candidates for applications such as gas storage and separation, catalysis, drug delivery and optoelectronics.^1,2^

The primary tool for the structure determination of MOFs and COFs is single-crystal X-ray diffraction. However, guest molecules in the pores—often disordered or partially occupied—can compromise data quality and necessitate solvent-masking software.^3^ Solvent exchange or removal can degrade the crystal structure, moreover, MOFs and COFs are also susceptible to twinning.^4^

Hydrogen atoms present in these materials create another challenge. Accurate hydrogen positions are essential due to the role of hydrogen atoms in the catalytic and conductive properties of MOFs^1^ and COFs.^5,6^ The Independent Atom Model (IAM),^7^ the most popular tool for X-ray data analysis, severely underestimates the bonds formed by hydrogen atoms. Hirshfeld atom refinement (HAR),^8,9^ which accounts for the aspherical atomic electron density, yields significantly improved hydrogen positions. In the case of the bond types typical of molecular crystals of organic compounds, IAM underestimates mean X–H bond lengths by 0.12 Å compared to the average neutron values,^10^ whereas HAR reduces the difference to 0.014 Å.^11^ HAR has also been successful at determining positions of hydrogen atoms bonded to transition metals^12,13^ with mean difference of 0.031 Å, compared to the neutron values. HAR has been extensively applied for molecular crystals, and software capable of handling disorder, constraints and fragmentation has been developed, including Tonto,^14^ HARt,^15^ NoSpherA2 ^16^ and DiSCaMB.^17,18^ This software has already been used to refine a few network structures,^15,16^ including one MOF.^19^ Despite the benefits of the molecular wave function based approach the most correct method for network structures is HAR with periodic boundary conditions (PBCs), available via the XHARPy package.^20^ This approach, applied to the ZIF-2 MOF, improved hydrogen positions and ADPs for nonsolvent atoms, equally as in the case of classical HAR.^19^ Here, we continue that initial study by validating classical and periodic HAR across 20 X-ray data sets of MOFs, COFs and other polymeric compounds.

To ensure diversity among network compounds, a search of the Cambridge Structural Database (CSD) was performed, excluding structures with disorder and unresolved errors. This yielded 104 MOF and 5 COF single-crystal X-ray structures. Additionally, 4 data sets of copper-containing polymeric compounds were included; 3 referred to the same structure investigated at ambient pressure and two temperatures (100 and 125 K) or at 1 GPa and 293 K (named SZ7–100K, SZ7–125K and SZ7–1GPa, respectively), and one remeasured structure, originally deposited in the CSD as ZOGKUD,^21^ here referred to as SZ3–150K. Experimental details for these Cu polymeric data sets are provided in the Supporting Information. IAM rerefinement performed for 109 structures was successful for 77 MOFs and 3 COFs. Solvent masking (SQUEEZE)^3^ and constraints on hydrogen positions and thermal motions were often necessary.

All successfully rerefined structures and the additional 4 data sets were subsequently subjected to classical HAR using DiSCaMB.^17^ Molecular wave functions were obtained via DFT with the B3LYP functional and either Def2-SVP or cc-pVDZ basis sets using clusters of atomic charges and dipoles on all molecules surrounding the central molecule within an 8 Å radius. DiSCaMB-HAR succeeded for 14 MOFs, 2 COFs and 4 Cu-polymers (25% total).

During DiSCaMB-HAR, most constraints on hydrogen positions and thermal motions were retained from IAM with a few exceptions. In total, 8 structures were refined with both methods without constraints. The 20 structures amenable to DiSCaMB-HAR were subjected to XHARPy-HAR,^20^ which uses real-space projector augmented wave (PAW) densities with 3-dimensional PBCs. Wave functions with PBCs were obtained via Γ-point DFT calculations with SCAN and PBE functionals. In some cases, additional constraints on hydrogen thermal motion were necessary. XHARPy-HAR was achievable for 1 MOF and 3 Cu-polymers measured at ambient pressure. Refinement details for each structure are provided in the Supporting Information. In the absence of neutron data for MOFs and COFs, geometry optimization results were used as a benchmark for bond lengths. Geometry optimizations, starting from IAM structures, were performed using either CASTEP with the PBE functional and C19 pseudopotentials or CRYSTAL17 with the B3LYP functional and the Def2-SVP basis set.

We discuss in detail the four structures for which XHARPy-HAR was attainable (SZ3–150K, SZ7–100K, SZ7–125K, and CUGLYM08) and the high-pressure structure SZ7–1GPa. The DiSCaMB-HAR and XHARPy-HAR structures and their overlays with the IAM structures are shown in Figure 1. Table 1 presents refinement statistics (R, wR2, GooF, and minimum and maximum residual density) and the X-ray X-H bond-related statistics, such as mean difference (MD) and mean absolute difference (MAD) from the optimized values. Full results, including residual density maps, are given in the Supporting Information.

**Figure 1:**
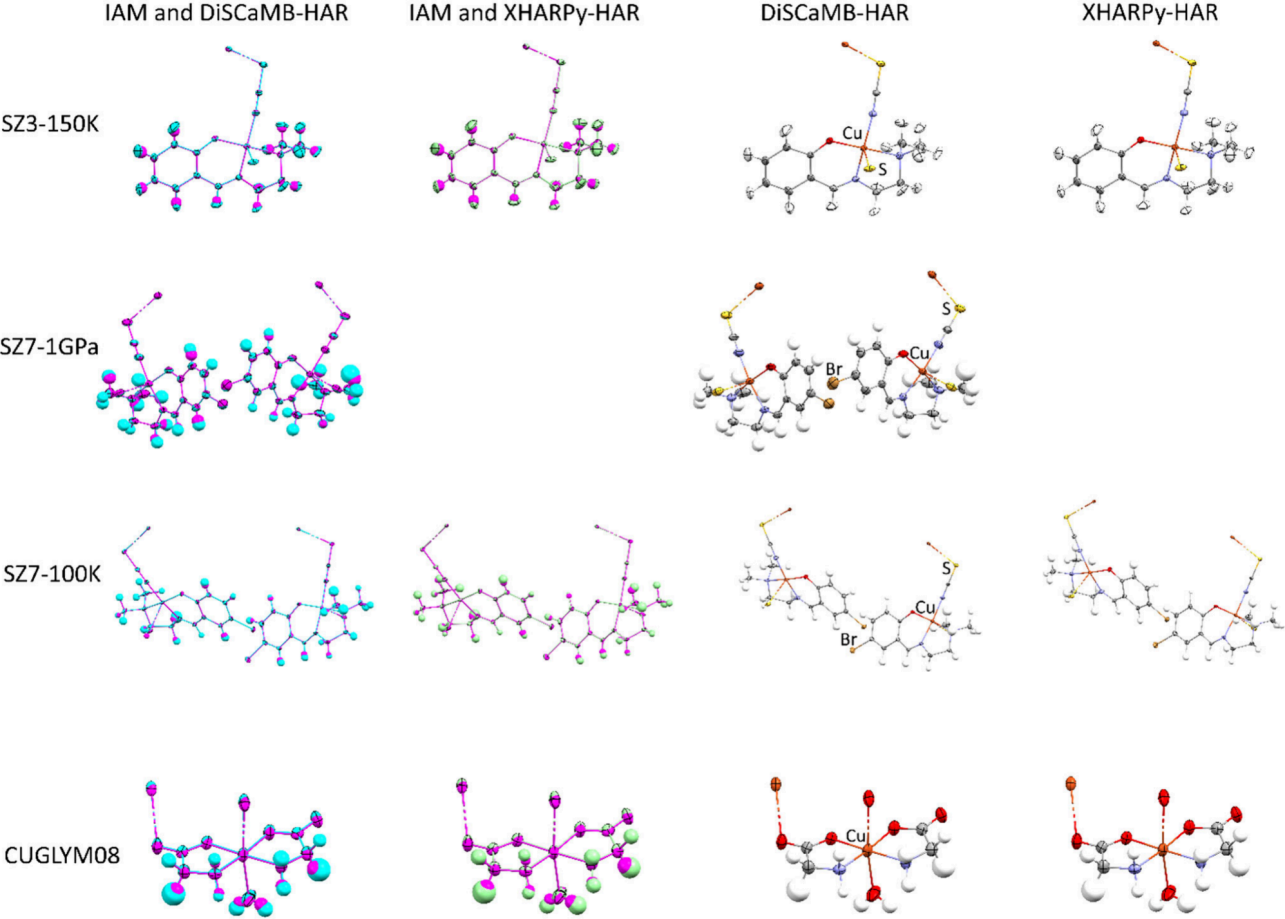
Single-crystal X-ray structures of SZ3–150K, SZ7–1GPa, SZ7–100K and CUGLYM08 obtained as a result of DiSCaMB-HAR and XHARPy-HAR (on the right). On the left, DiSCaMB-HAR (cyan) and XHARPy-HAR (light green) structures are overlaid with the IAM structures (magenta). Displacement ellipsoids are shown at the 50% probability level.

**Table 1:** Refinement Statistics (R, wR2, GooF, Minimum and Maximum Residual Density) for Five Single-Crystal X-ray Structures (SZ3-150K, SZ7-1GPa, SZ7-100K, SZ7-125K and CUGLYM08) for Three Refinement Types (IAM, DiSCaMB-HAR and XHARPy-HAR)^a^

**Identifier**	**Refinement type**	**R**	**wR2**	**GooF**	**Δρ**_**min/max**_ **[e/ Å** ^**3**^ **]**	**Refined H positions**	**H thermal motion**	**MD [Å]**	**MAD [Å]**	**Mean σ [Å]**
**SZ3–150K**	IAM	3.08%	7.47%	1.023	–1.008/0.569	All	Iso, all refined	–0.170	0.170	0.029
	DiSCaMB-HAR	2.63%	6.48%	0.906	–0.754/0.639	All	Anis, all refined	–0.017	0.019	0.026
	XHARPy-HAR	2.63%	5.35%	1.003	–0.815/0.573	All	Anis, all refined	–0.016	0.019	0.025
**SZ7–1GPa**	IAM	4.13%	7.41%	1.017	–0.919/0.924	All	Iso, all refined	–0.150	0.158	0.048
	DiSCaMB-HAR	3.93%	6.09%	1.015	–0.989/0.935	All	Iso, all refined	–0.005*	0.051	0.051
**SZ7–100K**	IAM	3.93%	7.46%	1.045	–0.673/0.598	All	Iso, all refined	–0.138	0.142	0.058
	DiSCaMB-HAR	3.77%	7.19%	1.006	–0.647/0.606	All	Iso, all refined	0.009*	0.049	0.063
	XHARPy-HAR	3.76%	5.19%	1.628	–0.691/0.780	All	Iso, 18 refined, 10 fixed	0.004*	0.034	0.051
**SZ7–125K**	IAM	1.97%	4.89%	1.059	–0.401/0.272	All	Iso, all refined	–0.161	0.161	0.040
	DiSCaMB-HAR	1.67%	4.15%	0.897	–0.418/0.278	All	Iso, all refined	–0.005*	0.025	0.039
	XHARPy-HAR	1.67%	3.39%	1.334	–0.452/0.343	All	Iso, all fixed	–0.007*	0.030	0.035
**CUGLYM08**	IAM	3.98%	8.70%	1.043	–0.543/0.469	All	Iso, all refined	–0.224	0.224	0.054
	DiSCaMB-HAR	3.75%	8.13%	0.974	–0.530/0.401	All	Iso, all refined	–0.071*	0.039	0.064
	XHARPy-HAR	3.76%	6.44%	1.132	–0.524/0.398	All	Iso, all refined	–0.021*	0.043	0.059

a Mean σ stands for bond length standard deviation averaged for all X-H bond lengths in a given structure. *Difference is statistically insignificant.

SZ3–150K was refined anisotropically with both HAR methods, yielding very similar displacement ellipsoids. Only one hydrogen atom showed a slightly different tilt. Both HARs produced very similar MD and MAD, 1 order of magnitude lower than IAM. SZ3–150K was characterized by the lowest mean standard deviation of X-H bonds of all of the structures across the refinement methods. Similarly, for CUGLYM08, MD and MAD from both types of HAR were 1 order of magnitude lower than from IAM. Whereas MAD in the case of CUGLYM08 was very similar for both types of HAR (DiSCaMB-HAR: 0.039 Å, XHARPy-HAR: 0.043 Å), MD was lower for DiSCaMB-HAR, suggesting a greater tendency toward shorter X-H bond lengths with this method. IAM provided much worse agreement with the optimized bond lengths (MAD = 0.224 Å). Both types of HAR improved R, wR2 (lower value for XHARPy-HAR) and residual density in comparison with IAM, while slightly worsened GOOF. For SZ7–125K R, wR2 and residual density were lower than for SZ7–100K, however, IAM resulted in slightly higher discrepancy from the optimized X-H bond lengths. Nevertheless, for both structures, both types of HAR considerably improved the agreement with theoretical X-H bond lengths: MD became nearly 0 and MAD was comparable to CUGLYM08.

IAM and DiSCaMB-HAR enabled refinement of isotropic hydrogen thermal vibrations for SZ7–100K and SZ7–125K, while XHARPy-HAR enabled refinement of thermal vibrations of most hydrogen atoms for SZ7–100K but required fixed hydrogen thermal vibrations for SZ7–125K. For SZ7–1GPa, XHARPy-HAR was not attainable; however, DiSCAMB-HAR was successful and matched the ambient pressure structures in terms of the agreement of X-H bond lengths with the optimized values. DiSCaMB-HAR also resulted in lower R and wR2, similar GOOF and slightly higher range of residual density than IAM. For the structures in Table 1., DiSCaMB-HAR and XHARPy-HAR decreased the R factor by a very similar value, compared to IAM. DiSCaMB-HAR provided lower wR2 than IAM and XHARPy-HAR reduced it further. However, XHARPy-HAR yielded the highest discrepancy of GooF from 1 among the applied methods. No particular trend in residual density extremes was observed across the methods.

Analysis of the average MD and MAD for the X-H bond lengths in all 20 structures provides valuable observations (Table 2). The X-ray values were compared both to the optimized values and mean neutron bond lengths.^22^ IAM yields MD = −0.125 Å for the optimized values and MD = −0.118 Å for mean neutron values – consistent with prior studies of molecular organic single-crystal X-ray structures^10^ (MD = −0.12 Å). Corresponding MAD values are 0.154 and 0.134 Å, respectively. DiSCaMB-HAR reduces them by half, to 0.082 and 0.066 Å. MD between DiSCaMB-HAR and the optimized X-H bond lengths is −0.025 Å, almost twice as high as in the study of molecular organic crystals^10^ (−0.014 Å), however, it is reduced to −0.010 Å when mean neutron values are the reference. Both MDs are statistically insignificant; therefore, DiSCaMB-HAR applied to polymeric compounds provides X-H bond lengths in statistical agreement with both geometry-optimized and average neutron distances. XHARPy-HAR shows even better agreement: MD = −0.007 Å and MAD = 0.031 Å for comparison with the optimized and mean neutron distances. Across the 20 structures, the method, HAR or IAM, that yields better refinement statistics or residual density maps (see Supporting Information) varies by the structure.

**Table 2:** Statistics Calculated for X-H Bond Lengths for All the Examined Single-Crystal X-ray Structures for Three Refinement Types (IAM, DiSCaMB-HAR and XHARPy-HAR)^a^

**Reference**	**Refinement type**	**MD [Å]**	**MAD [Å]**	**Mean σ [Å]**	**structures**	**bonds**
**Optimized**	IAM	–0.125	0.154	0.047	20	224
	DiSCaMB-HAR	–0.025*	0.082	0.049	20	213
	XHARPy-HAR	–0.007*	0.031	0.038	4	81
**Average neutron**	IAM	–0.118	0.134	0.047	20	183
	DiSCaMB-HAR	–0.010*	0.066	0.049	20	183
	XHARPy-HAR	–0.007*	0.031	0.038	4	82

a Mean σ stands for bond length standard deviation averaged for all X-H bond lengths. *Difference is statistically insignificant.

To assess how data/refinement quality affects hydrogen positions, we plotted MAD between the theoretical X-H bond lengths and the experimental values (Figure 2). The 20 crystal structures in the plots were arranged according to data quality (ranking based on data completeness, resolution, and R_int_) and refinement quality (ranking established on R, wR2, GooF and residual density range). The details of the ranking procedure are available in the Supporting Information. XHARPy-HAR was feasible only for the structures from the top of the data-refinement quality ranking. In the top 7 structures, MAD from DiSCaMB-HAR (and XHARPy-HAR) was clearly lower than that from IAM. With decreasing position in the ranking, the difference between the methods decreases and the error bars for HAR and IAM start overlapping. In the bottom half of the structures, HAR and IAM alternately show higher discrepancy with the theoretical X-H bond lengths, and no method has a general advantage. The discrepancies are always statistically significant except the cases marked in Table 1.

**Figure 2:**
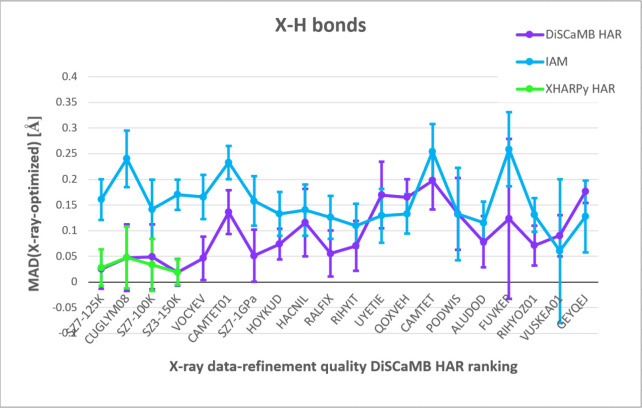
Mean absolute difference between the optimized bond lengths and the experimental bond lengths obtained with three refinement types (IAM, DiSCaMB-HAR and XHARPy-HAR) against X-ray data for 20 polymeric structures arranged according to the DiSCaMB-HAR data-refinement quality ranking from the best to the worst. The error bars represent average standard deviation of X-H bond lengths for a given structure.

In summary, we refined 20 single-crystal X-ray structures of polymeric compounds, including MOFs and COFs, using IAM and two types of HAR (DiSCaMB and XHARPy). On average, DiSCaMB-HAR improved the agreement of the bond lengths formed by hydrogen atoms with the optimized values, reducing MAD almost twice, as compared to IAM. XHARPy-HAR could be achieved for 20% of the structures characterized by the highest data and refinement quality, yielding hydrogen positions very similar to DiSCaMB-HAR. The advantage of HAR over IAM was strongly pronounced for one-third of all the structures characterized by the highest data and refinement quality. The less computationally demanding version of HAR based on molecular wave function seems sufficient to obtain improvement of hydrogen positions, provided that X-ray data of adequate quality is available. Feasibility of applying HAR to structures of polymeric compounds, such as MOFs and COFs, opens a pathway to investigating structure–property relationships in materials, where an accurate description of hydrogen atoms is crucial. These include processes governed by hydrogen bonding and other noncovalent interactions, e.g., guest binding, framework flexibility and stability, gas sorption, catalysis, and proton conductivity. HAR provides improved crystal structures without resorting to neutron diffraction, thereby facilitating the discovery of materials with tailored properties.

## Supplementary Material





## Data Availability

All data needed to evaluate the conclusions in the paper are present in the paper and/or the . Additional data related to this paper may be requested from the authors.

## Notes

Crystallographic data (ZIP)
Experimental and computational details of the investigated crystal structures, residual density maps and plots presenting refinement-related statistics (PDF)

## Notes

CCDC Deposition Numbers 2447206–2447249 contain the supplementary crystallographic data for this paper.
