# Supplementary material for: Improving Polymeric Structures with Hirshfeld Atom Refinement: A Study on MOFs and COFs
Source: ACS Mater Au. 2025 Jul 31;5(5):767–71. doi: 10.1021/acsmaterialsau.5c00076 (PMC12426777; doi:10.1021/acsmaterialsau.5c00076)
Supplement: Supplementary file 2 [file mg5c00076_si_002.pdf]

Improving Polymeric Structures with Hirshfeld Atom Refinement: A Study on MOFs and COFs

Magdalena Woińska, Anna Makal, Paweł Grzymski-Ostręga, Michał L. Chodkiewicz, Krzysztof Woźniak

Faculty of Chemistry, University of Warsaw, Pasteura 1, Warsaw, 02-093, Poland
Correspondence to: magdalena.woinska@uw.edu.pl, kwozniak@chem.uw.edu.pl

1 Residual density maps

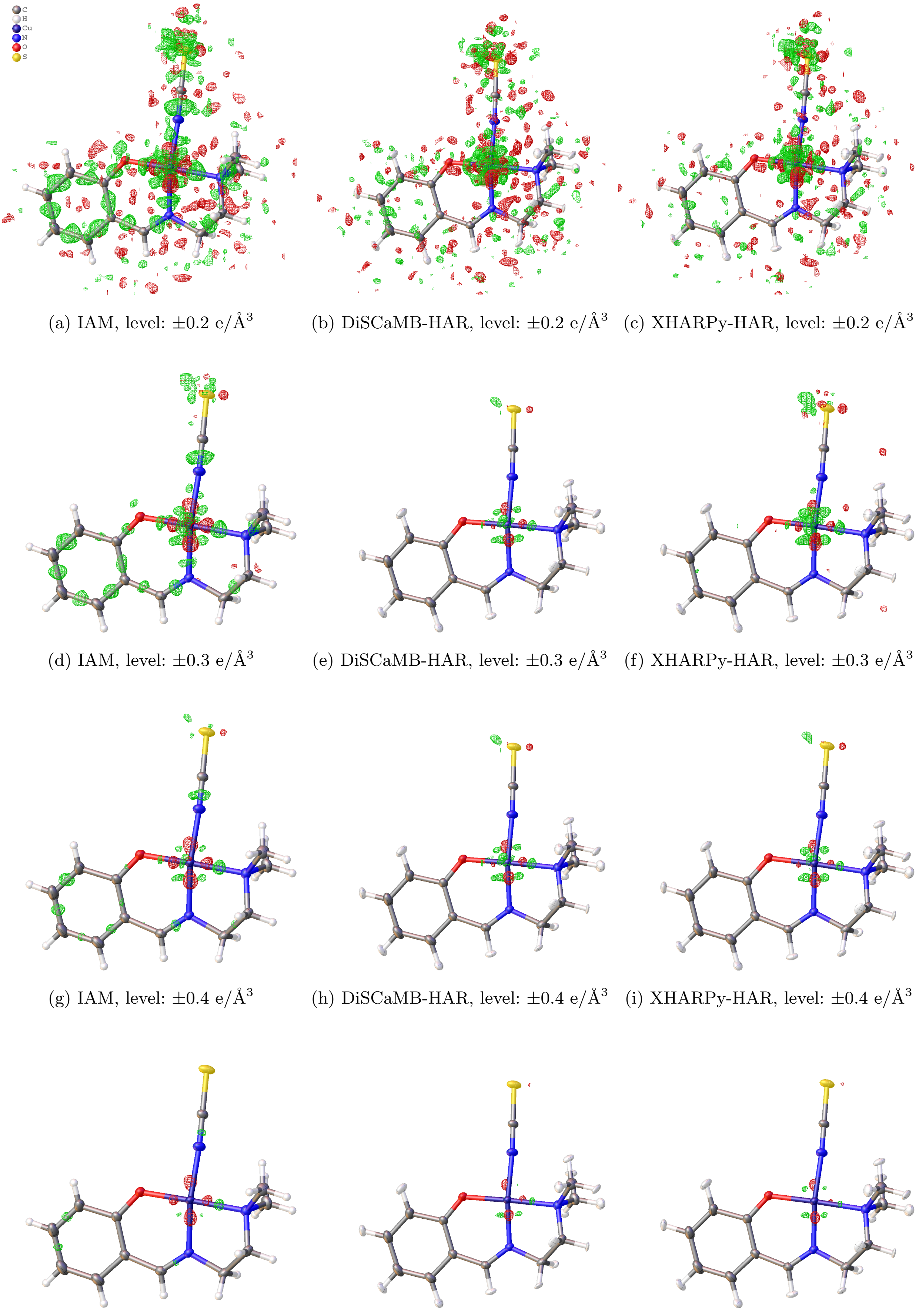

(a) IAM, level: ±0.2 e/Å^3 (b) DiSCaMB-HAR, level: ±0.2 e/Å^3 (c) XHARPy-HAR, level: ±0.2 e/Å^3

(d) IAM, level: ±0.3 e/Å^3 (e) DiSCaMB-HAR, level: ±0.3 e/Å^3 (f) XHARPy-HAR, level: ±0.3 e/Å^3

(g) IAM, level: ±0.4 e/Å^3 (h) DiSCaMB-HAR, level: ±0.4 e/Å^3 (i) XHARPy-HAR, level: ±0.4 e/Å^3

(j) IAM, level: ±0.5 e/Å^3 (k) DiSCaMB-HAR, level: ±0.5 e/Å^3 (l) XHARPy-HAR, level: ±0.5 e/Å^3

Figure S 1: Residual density for SZ3-150K obtained with various refinement techniques.

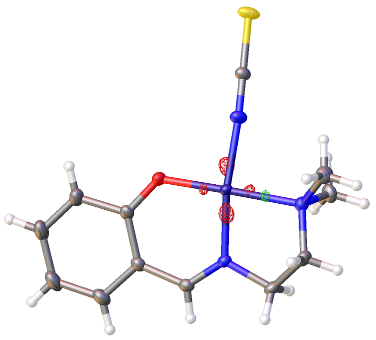

(a) IAM, level: ±0.6 e/Å^3

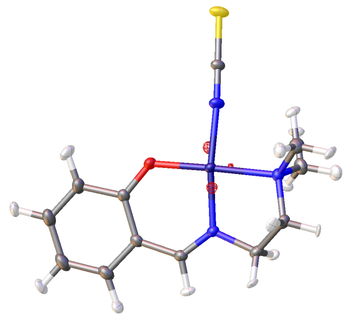

(b) DiSCaMB-HAR, level: ±0.6 e/Å^3

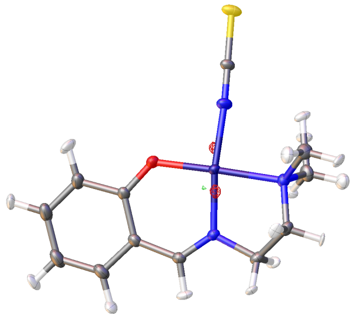

(c) XHARPy-HAR, level: ±0.6 e/Å^3

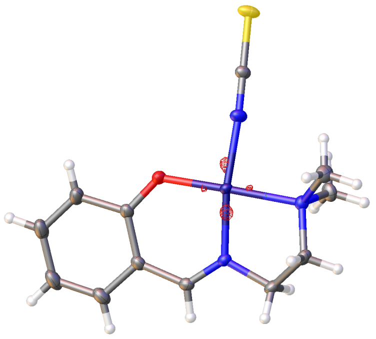

(d) IAM, level: ±0.7 e/Å^3

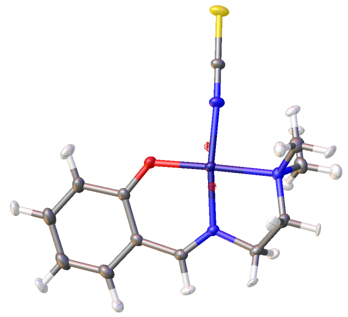

(e) DiSCaMB-HAR, level: ±0.7 e/Å^3

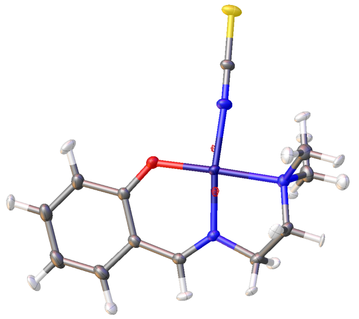

(f) XHARPy-HAR, level: ±0.7 e/Å^3

Figure S 2: Residual density for SZ3-150K obtained with various refinement techniques.

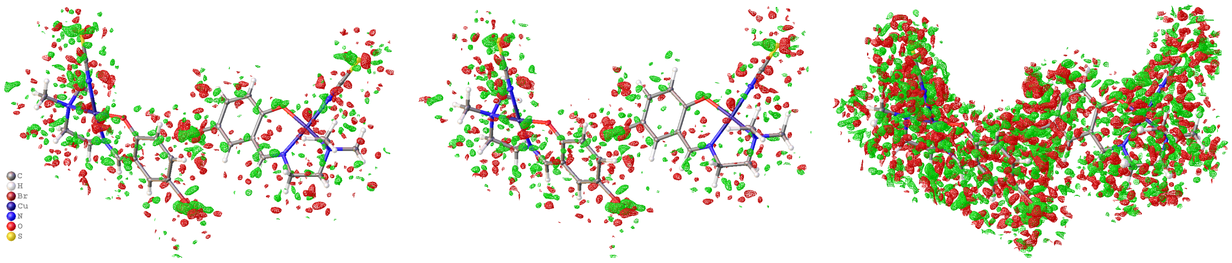

(a) IAM, level: ±0.2 e/Å^3 (b) DiSCaMB-HAR, level: ±0.2 e/Å^3 (c) XHARPy-HAR, level: ±0.2 e/Å^3

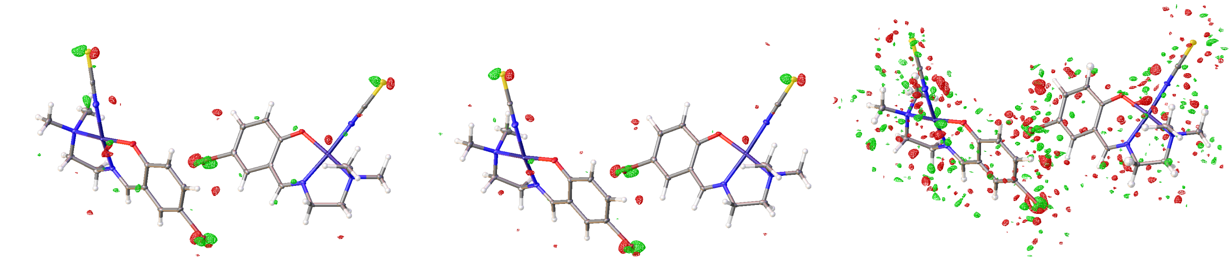

(d) IAM, level: ±0.3 e/Å^3 (e) DiSCaMB-HAR, level: ±0.3 e/Å^3 (f) XHARPy-HAR, level: ±0.3 e/Å^3

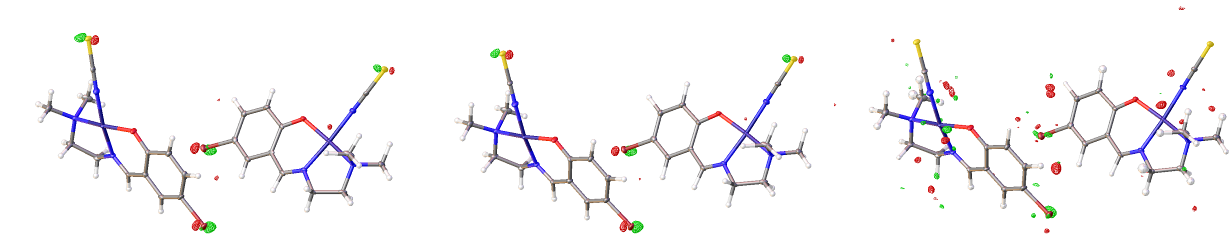

(g) IAM, level: ±0.4 e/Å^3 (h) DiSCaMB-HAR, level: ±0.4 e/Å^3 (i) XHARPy-HAR, level: ±0.4 e/Å^3

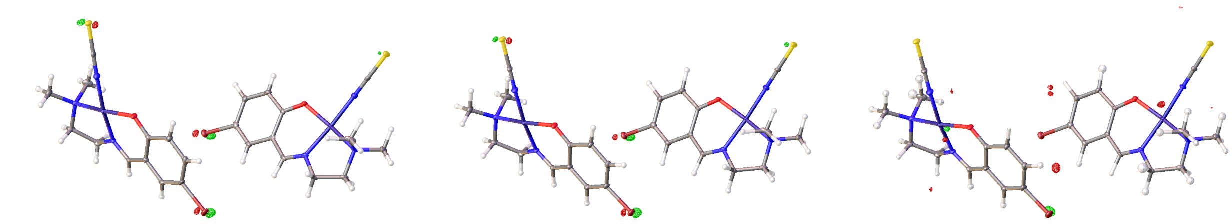

(j) IAM, level: ±0.5 e/Å^3 (k) DiSCaMB-HAR, level: ±0.5 e/Å^3 (l) XHARPy-HAR, level: ±0.5 e/Å^3

Figure S 3: Residual density for SZ7-100K obtained with various refinement techniques.

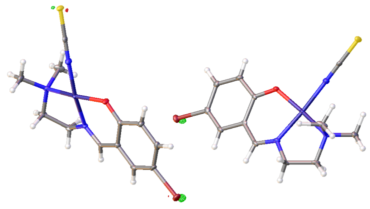

(a) IAM, level: ±0.6 e/Å^3

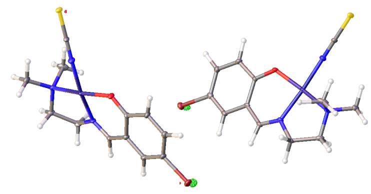

(b) DiSCaMB-HAR, level: ±0.6 e/Å^3

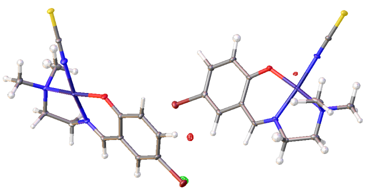

(c) XHARPy-HAR, level: ±0.6 e/Å^3

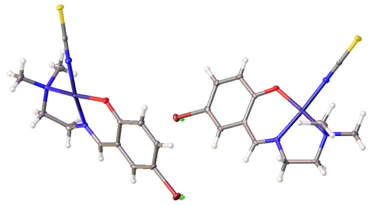

(d) IAM, level: ±0.7 e/Å^3

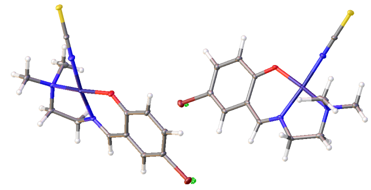

(e) DiSCaMB-HAR, level: ±0.7 e/Å^3

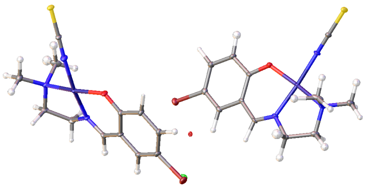

(f) XHARPy-HAR, level: ±0.7 e/Å^3

Figure S 4: Residual density for SZ7-100K obtained with various refinement techniques.

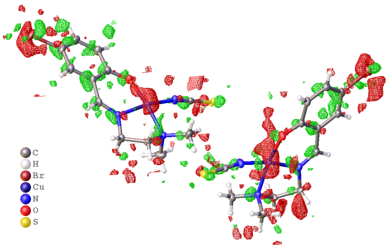

(a) IAM, level: ±0.1 e/Å^3

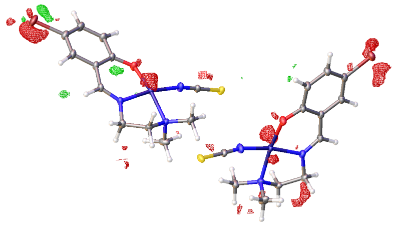

(b) DiSCaMB-HAR, level: ±0.1 e/Å^3

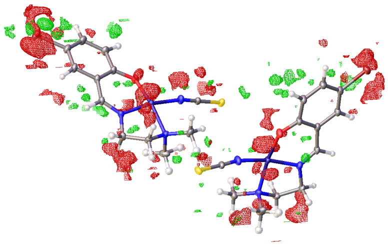

(c) XHARPy-HAR, level: ±0.1 e/Å^3

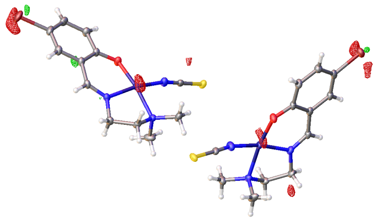

(d) IAM, level: ±0.2 e/Å^3

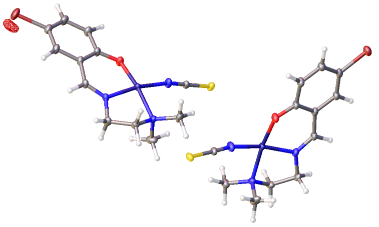

(e) DiSCaMB-HAR, level: ±0.2 e/Å^3

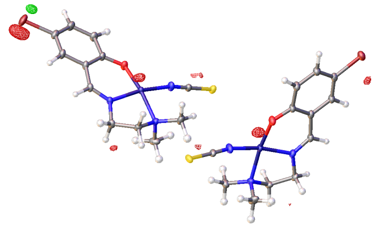

(f) XHARPy-HAR, level: ±0.2 e/Å^3

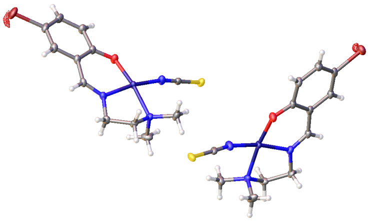

(g) IAM, level: ±0.3 e/Å^3

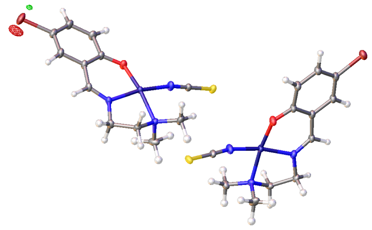

(h) XHARPy-HAR, level: ±0.3 e/Å^3

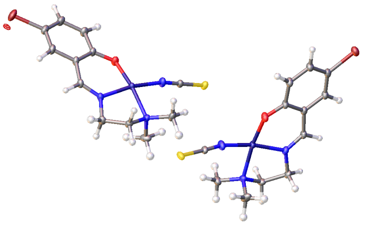

(i) XHARPy-HAR, level: ±0.4 e/Å^3

Figure S 5: Residual density for SZ7-125K obtained with various refinement techniques.

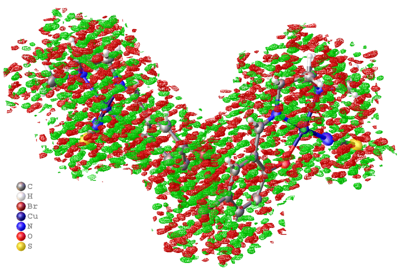

(a) IAM, level: ±0.3 e/Å^3

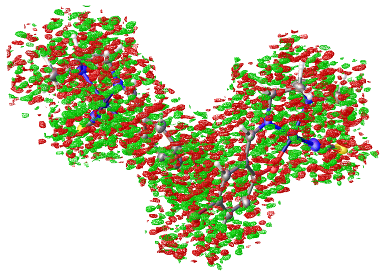

(b) DiSCaMB-HAR, level: ±0.3 e/Å^3

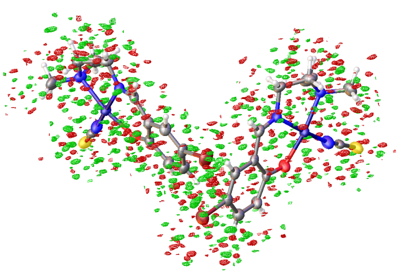

(c) IAM, level: ±0.4 e/Å^3

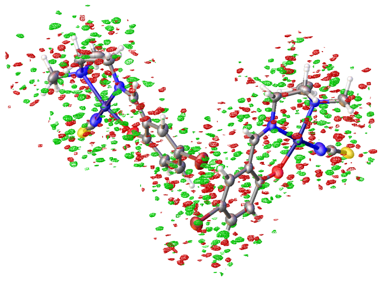

(d) DiSCaMB-HAR, level: ±0.4 e/Å^3

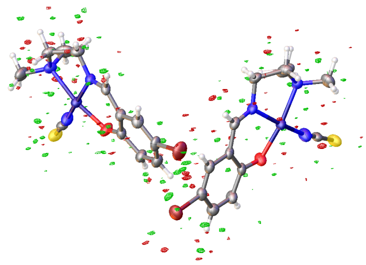

(e) IAM, level: ±0.5 e/Å^3

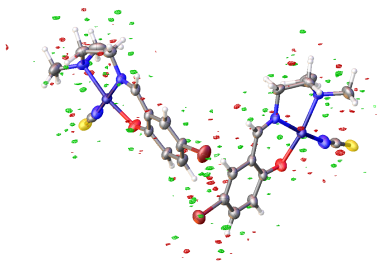

(f) DiSCaMB-HAR, level: ±0.5 e/Å^3

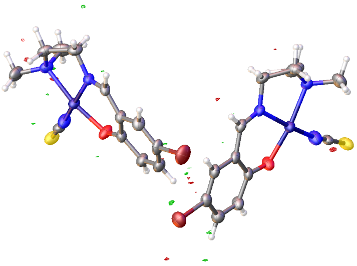

(g) IAM, level: ±0.6 e/Å^3

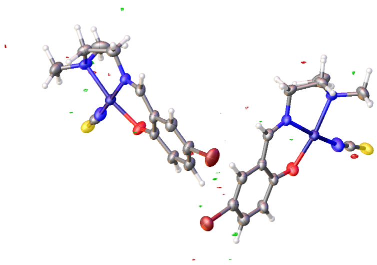

(h) DiSCaMB-HAR, level: ±0.6 e/Å^3

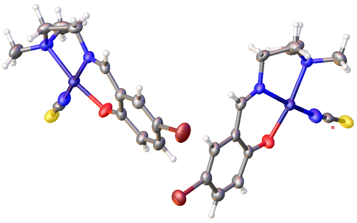

(i) IAM, level: ±0.7 e/Å^3

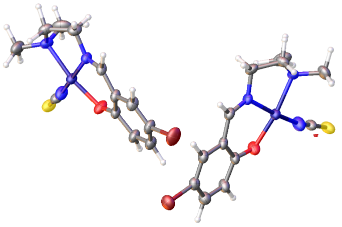

(j) DiSCaMB-HAR, level: ±0.7 e/Å^3

Figure S 6: Residual density for SZ7-1GPa obtained with various refinement techniques.

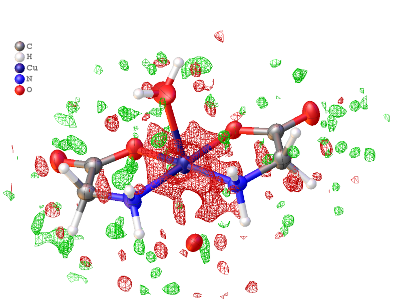

(a) DiSCaMB-HAR, level: ±0.2 e/Å^3

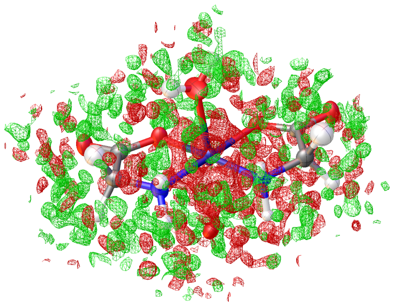

(b) XHARPy-HAR, level: ±0.2 e/Å^3

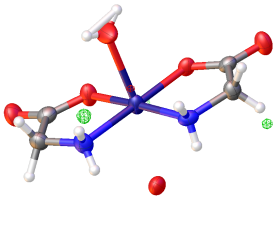

(c) IAM, level: ±0.3 e/Å^3

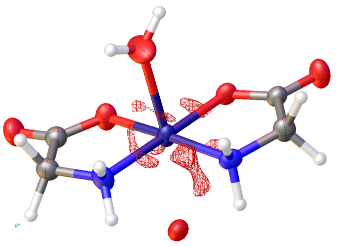

(d) DiSCaMB-HAR, level: ±0.3 e/Å^3

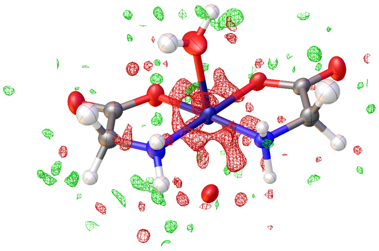

(e) XHARPy-HAR, level: ±0.3 e/Å^3

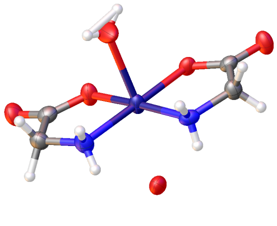

(f) IAM, level: ±0.4 e/Å^3

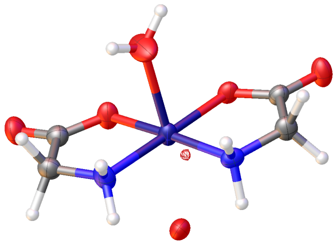

(g) DiSCaMB-HAR, level: ±0.4 e/Å^3

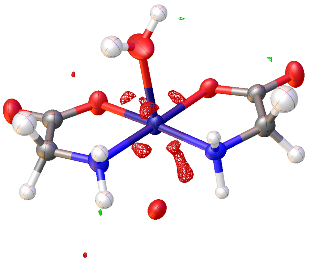

(h) XHARPy-HAR, level: ±0.4 e/Å^3

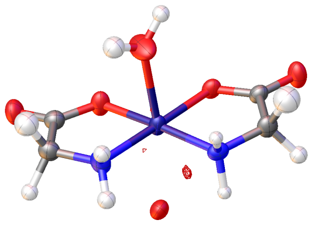

(i) XHARPy-HAR, level: ±0.5 e/Å^3

Figure S 7: Residual density for CUGLYM08 obtained with various refinement techniques.

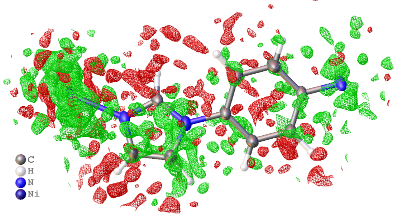

(a) IAM, level: ±0.3 e/Å^3

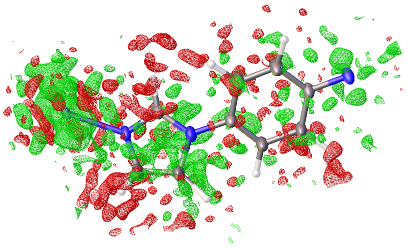

(b) DiSCaMB-HAR, level: ±0.3 e/Å^3

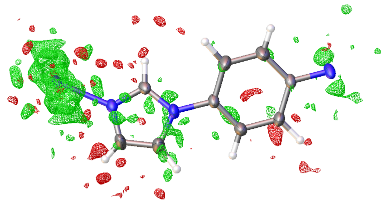

(c) IAM, level: ±0.4 e/Å^3

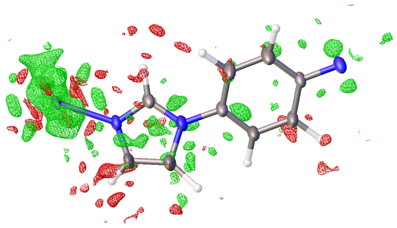

(d) DiSCaMB-HAR, level: ±0.4 e/Å^3

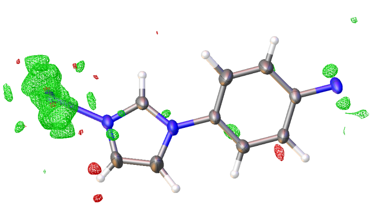

(e) IAM, level: ±0.5 e/Å^3

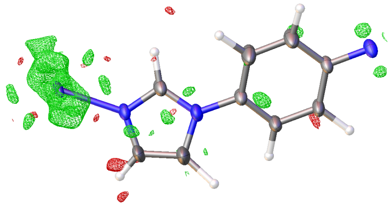

(f) DiSCaMB-HAR, level: ±0.5 e/Å^3

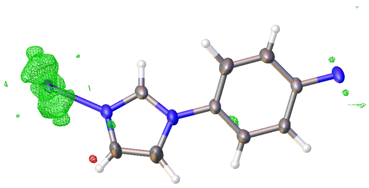

(g) IAM, level: ±0.6 e/Å^3

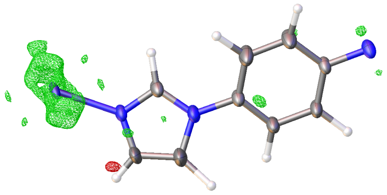

(h) DiSCaMB-HAR, level: ±0.6 e/Å^3

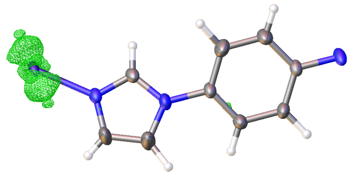

(i) IAM, level: ±0.7 e/Å^3

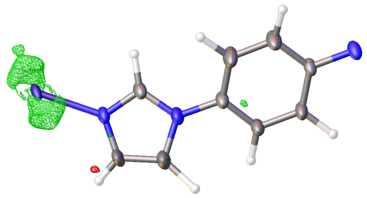

(j) DiSCaMB-HAR, level: ±0.7 e/Å^3

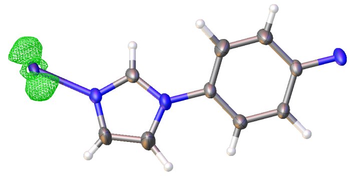

(k) IAM, level: ±0.8 e/Å^3

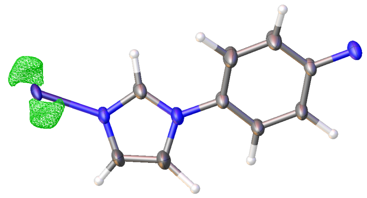

(l) DiSCaMB-HAR, level: ±0.8 e/Å^3

Figure S 8: Residual density for ALUDOD obtained with various refinement techniques.

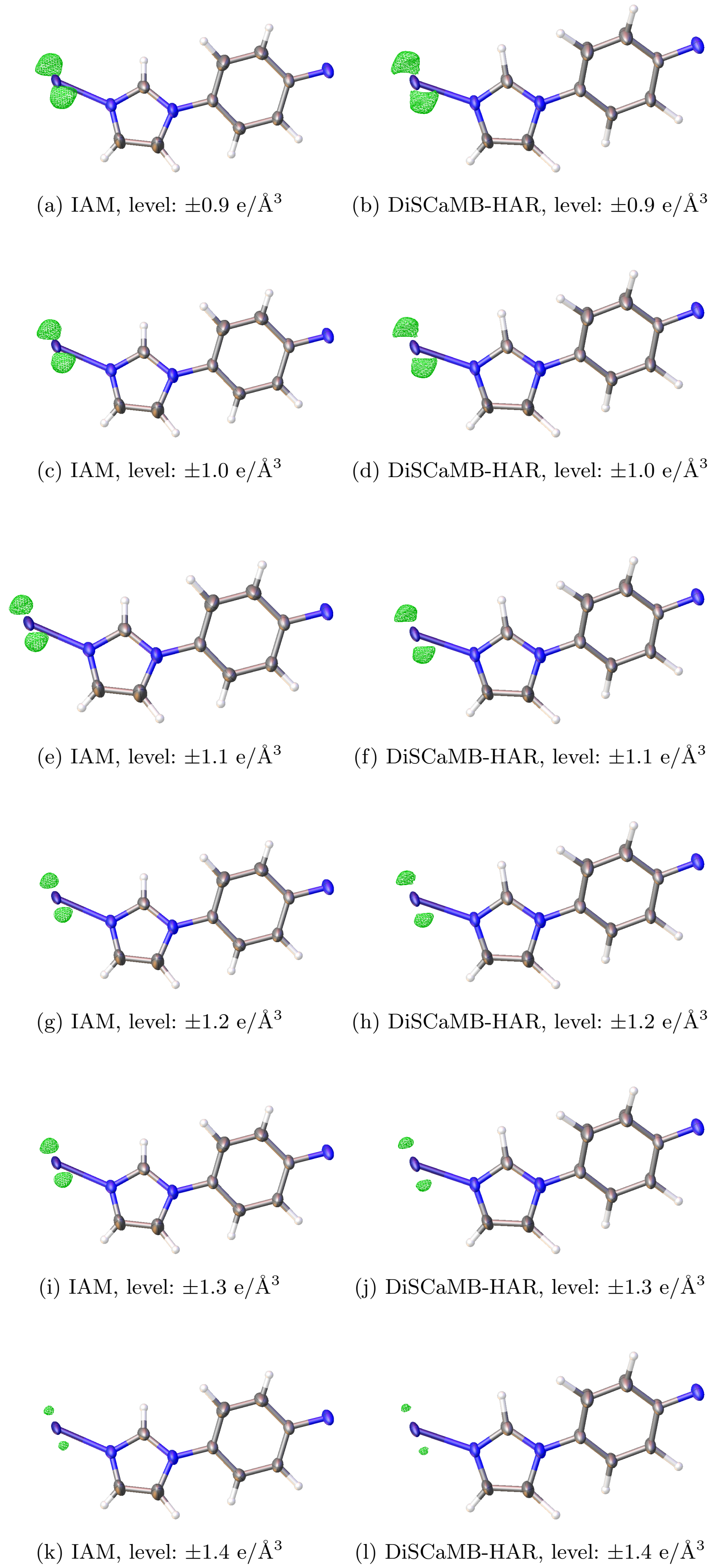

(a) IAM, level: ±0.9 e/Å^3
(b) DiSCaMB-HAR, level: ±0.9 e/Å^3
(c) IAM, level: ±1.0 e/Å^3
(d) DiSCaMB-HAR, level: ±1.0 e/Å^3
(e) IAM, level: ±1.1 e/Å^3
(f) DiSCaMB-HAR, level: ±1.1 e/Å^3
(g) IAM, level: ±1.2 e/Å^3
(h) DiSCaMB-HAR, level: ±1.2 e/Å^3
(i) IAM, level: ±1.3 e/Å^3
(j) DiSCaMB-HAR, level: ±1.3 e/Å^3
(k) IAM, level: ±1.4 e/Å^3
(l) DiSCaMB-HAR, level: ±1.4 e/Å^3

Figure S 9: Residual density for ALUDOD obtained with various refinement techniques.

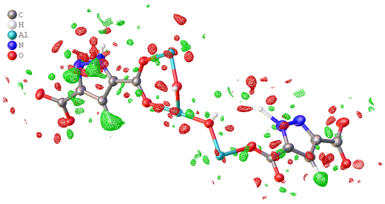

(a) IAM, level: ±0.3 e/Å^3

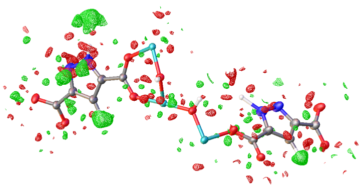

(b) DiSCaMB-HAR, level: ±0.3 e/Å^3

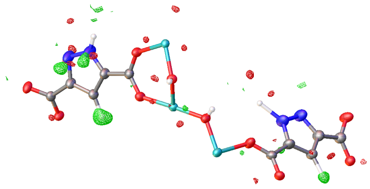

(c) IAM, level: ±0.4 e/Å^3

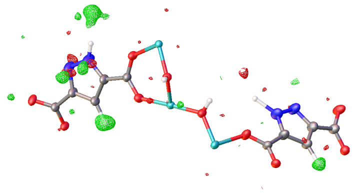

(d) DiSCaMB-HAR, level: ±0.4 e/Å^3

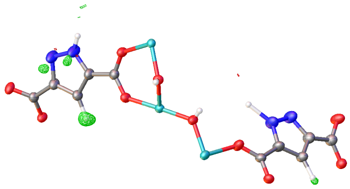

(e) IAM, level: ±0.5 e/Å^3

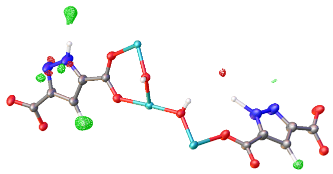

(f) DiSCaMB-HAR, level: ±0.5 e/Å^3

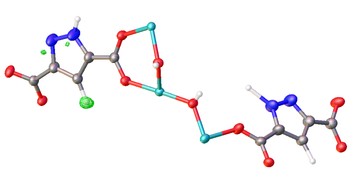

(g) IAM, level: ±0.6 e/Å^3

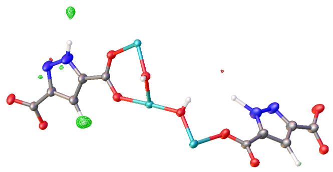

(h) DiSCaMB-HAR, level: ±0.6 e/Å^3

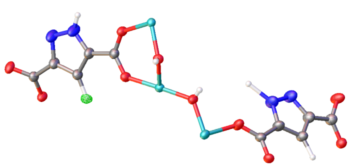

(i) IAM, level: ±0.7 e/Å^3

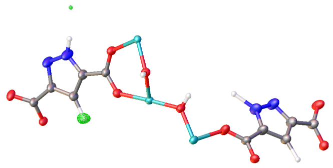

(j) DiSCaMB-HAR, level: ±0.7 e/Å^3

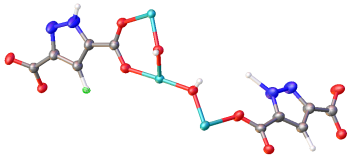

(k) IAM, level: ±0.8 e/Å^3

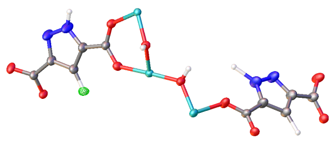

(l) DiSCaMB-HAR, level: ±0.8 e/Å^3

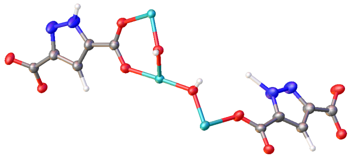

(m) IAM, level: ±0.9 e/Å^3

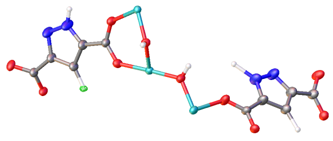

(n) DiSCaMB-HAR, level: ±0.9 e/Å^3

Figure S 10: Residual density for CAMTET obtained with various refinement techniques.

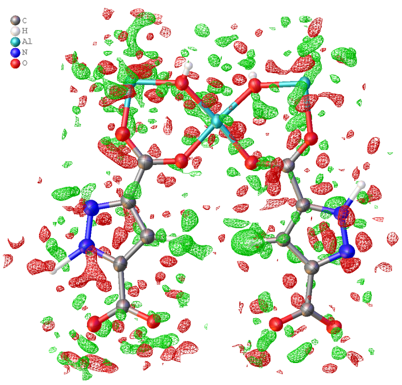

(a) IAM, level: ±0.2 e/Å^3

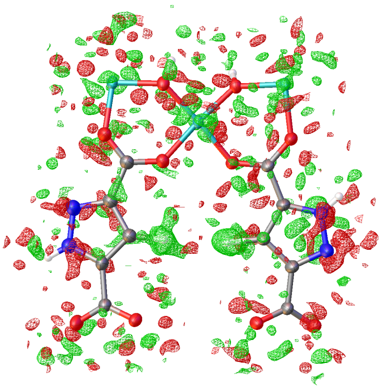

(b) DiSCaMB-HAR, level: ±0.2 e/Å^3

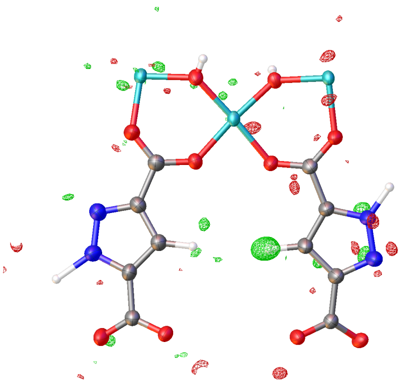

(c) IAM, level: ±0.3 e/Å^3

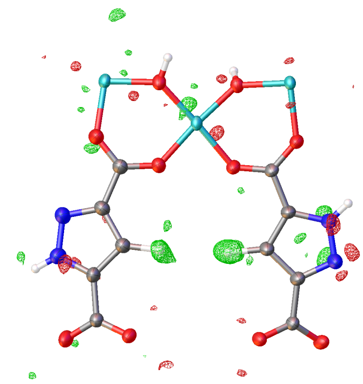

(d) DiSCaMB-HAR, level: ±0.3 e/Å^3

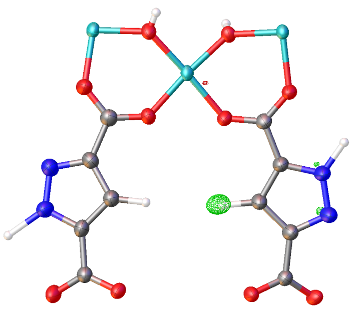

(e) IAM, level: ±0.4 e/Å^3

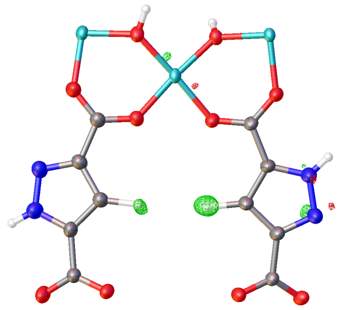

(f) DiSCaMB-HAR, level: ±0.4 e/Å^3

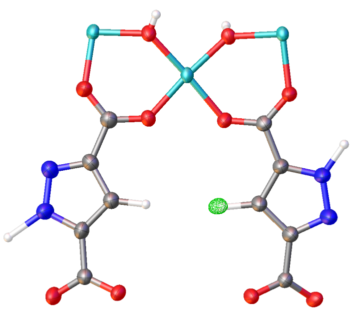

(g) IAM, level: ±0.5 e/Å^3

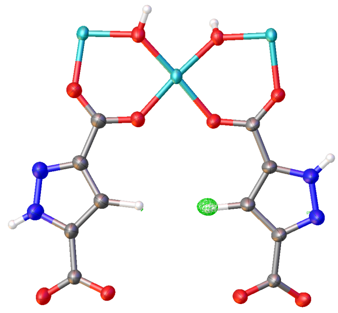

(h) DiSCaMB-HAR, level: ±0.5 e/Å^3

Figure S 11: Residual density for CAMTET01 obtained with various refinement techniques.

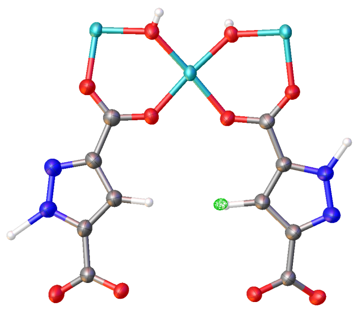

(a) IAM, level: ±0.6 e/Å^3

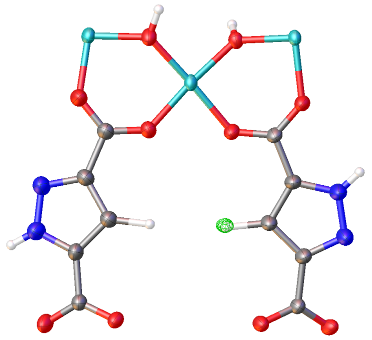

(b) DiSCaMB-HAR, level: ±0.6 e/Å^3

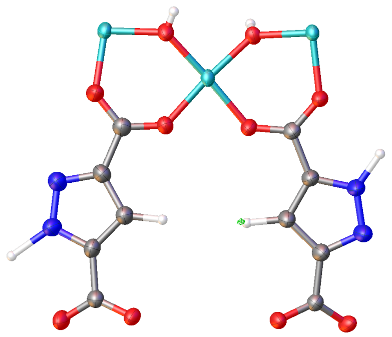

(c) IAM, level: ±0.7 e/Å^3

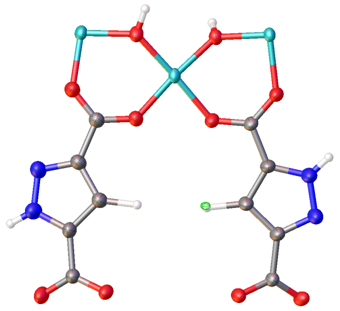

(d) DiSCaMB-HAR, level: ±0.7 e/Å^3

Figure S 12: Residual density for CAMTET01 obtained with various refinement techniques.

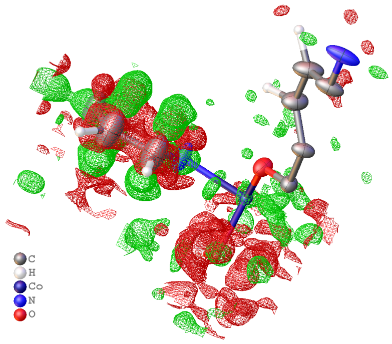

(a) DiSCaMB-HAR, level: ±0.3 e/Å^3

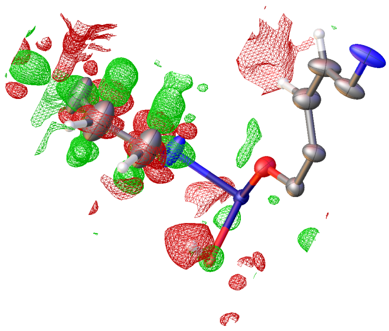

(b) IAM, level: ±0.5 e/Å^3

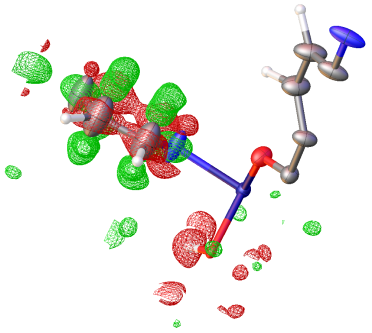

(c) DiSCaMB-HAR, level: ±0.5 e/Å^3

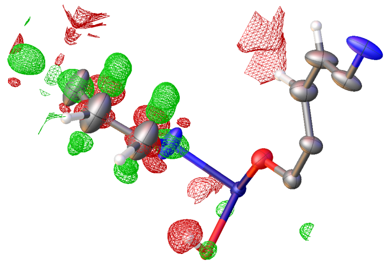

(d) IAM, level: ±0.7 e/Å^3

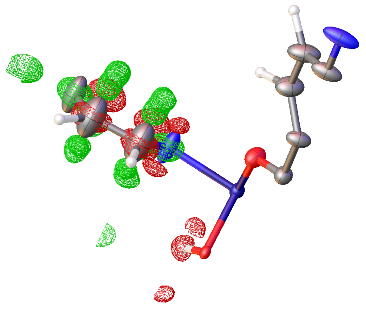

(e) DiSCaMB-HAR, level: ±0.7 e/Å^3

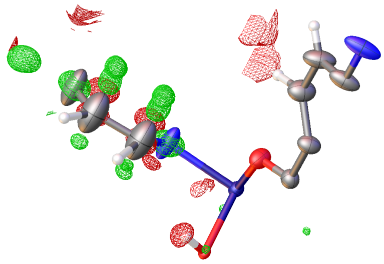

(f) IAM, level: ±0.9 e/Å^3

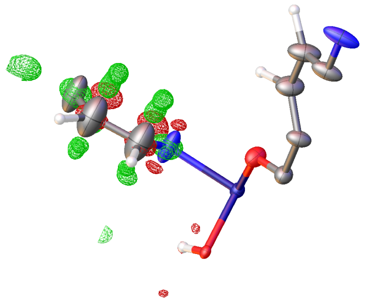

(g) DiSCaMB-HAR, level: ±0.9 e/Å^3

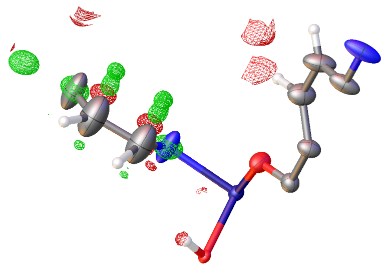

(h) IAM, level: ±1.1 e/Å^3

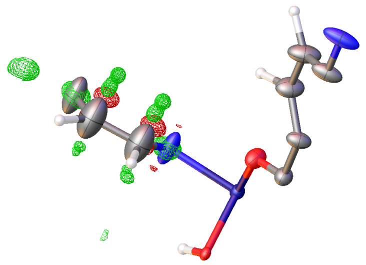

(i) DiSCaMB-HAR, level: ±1.1 e/Å^3

Figure S 13: Residual density for FUVKEP obtained with various refinement techniques.

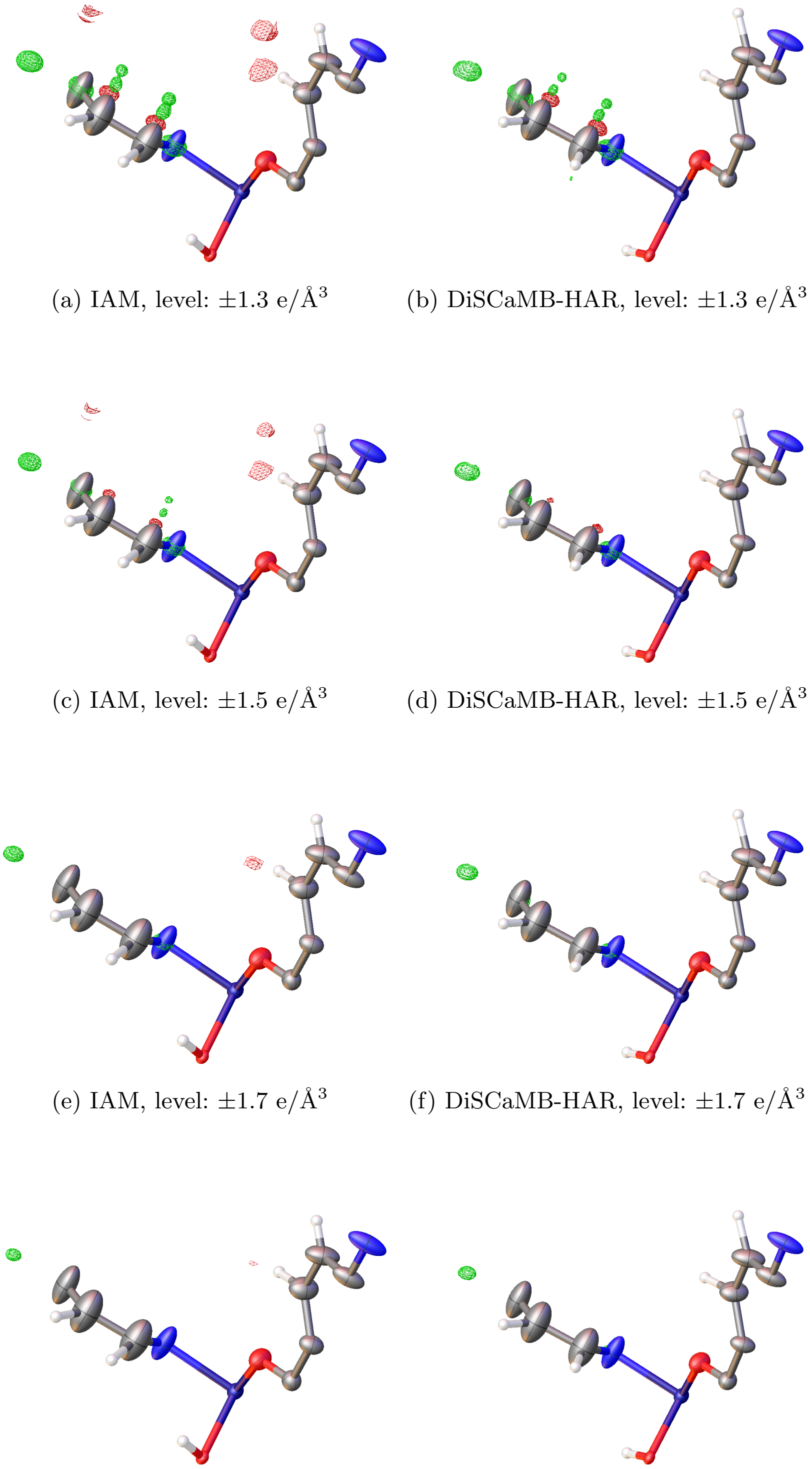

(a) IAM, level: ±1.3 e/Å^3 (b) DiSCaMB-HAR, level: ±1.3 e/Å^3

(c) IAM, level: ±1.5 e/Å^3 (d) DiSCaMB-HAR, level: ±1.5 e/Å^3

(e) IAM, level: ±1.7 e/Å^3 (f) DiSCaMB-HAR, level: ±1.7 e/Å^3

(g) IAM, level: ±1.9 e/Å^3 (h) DiSCaMB-HAR, level: ±1.9 e/Å^3

Figure S 14: Residual density for FUVKEP obtained with various refinement techniques.

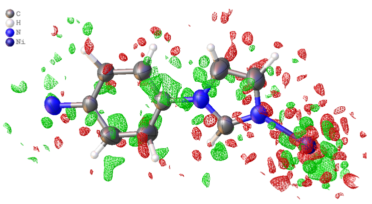

(a) IAM, level: ±0.3 e/Å^3

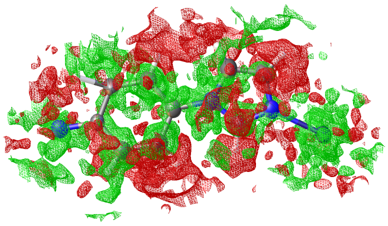

(b) DiSCaMB-HAR, level: ±0.3 e/Å^3

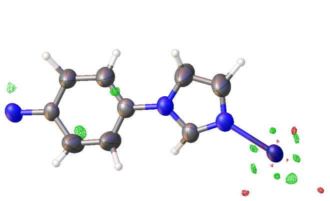

(c) IAM, level: ±0.5 e/Å^3

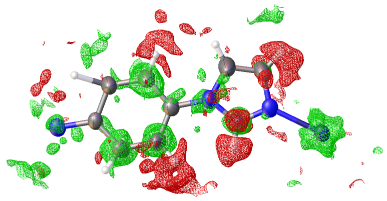

(d) DiSCaMB-HAR, level: ±0.5 e/Å^3

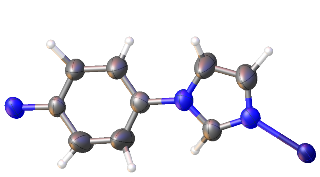

(e) IAM, level: ±0.7 e/Å^3

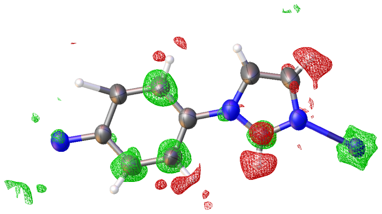

(f) DiSCaMB-HAR, level: ±0.7 e/Å^3

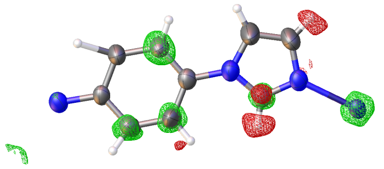

(g) DiSCaMB-HAR, level: ±0.9 e/Å^3

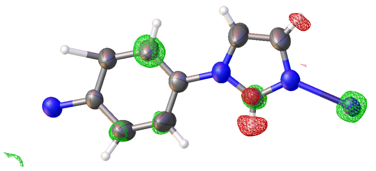

(h) DiSCaMB-HAR, level: ±1.1 e/Å^3

Figure S 15: Residual density for GEYQEJ obtained with various refinement techniques.

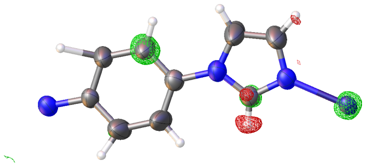

(a) DiSCaMB-HAR, level: ±1.3 e/Å^3

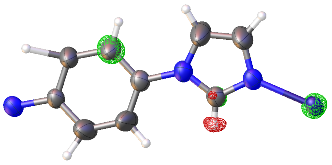

(b) DiSCaMB-HAR, level: ±1.5 e/Å^3

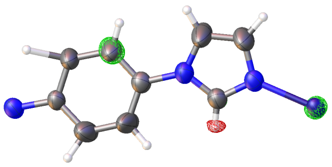

(c) DiSCaMB-HAR, level: ±1.7 e/Å^3

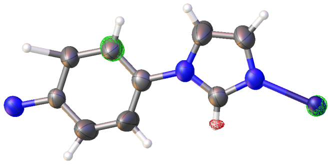

(d) DiSCaMB-HAR, level: ±1.9 e/Å^3

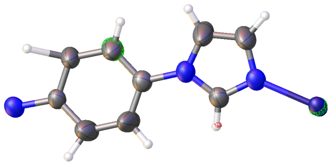

(e) DiSCaMB-HAR, level: ±2.1 e/Å^3

Figure S 16: Residual density for GEYQEJ obtained with various refinement techniques.

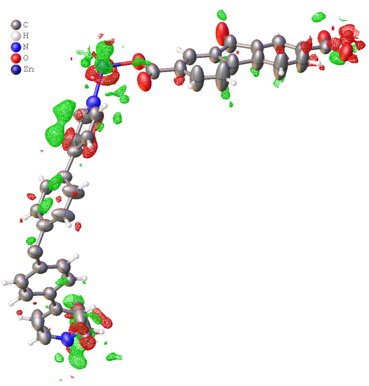

(a) IAM, level: ±0.2 e/Å^3

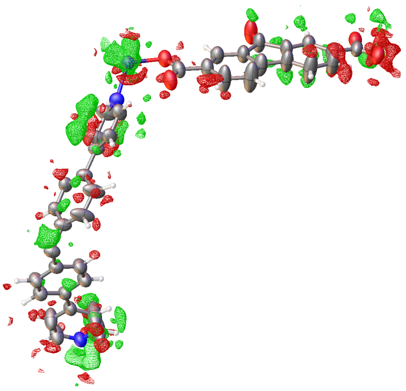

(b) DiSCaMB-HAR, level: ±0.2 e/Å^3

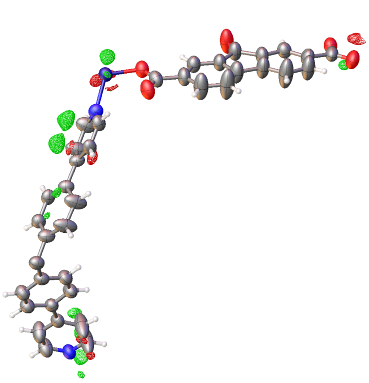

(c) IAM, level: ±0.3 e/Å^3

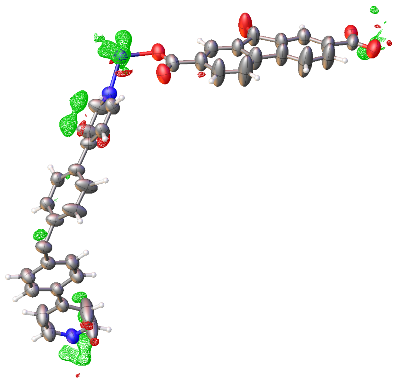

(d) DiSCaMB-HAR, level: ±0.3 e/Å^3

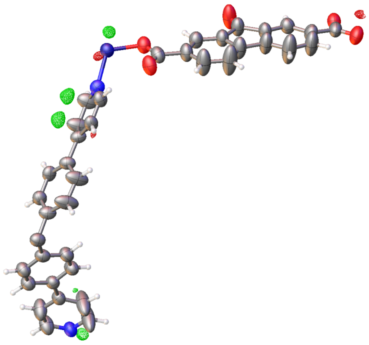

(e) IAM, level: ±0.4 e/Å^3

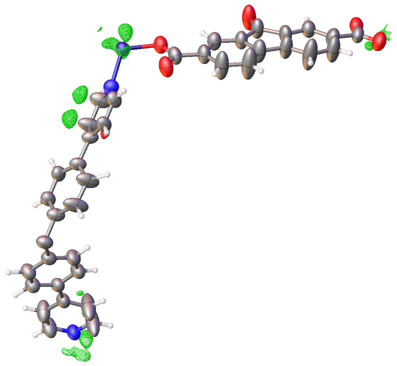

(f) DiSCaMB-HAR, level: ±0.4 e/Å^3

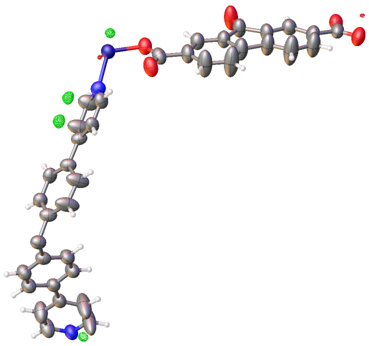

(g) IAM, level: ±0.5 e/Å^3

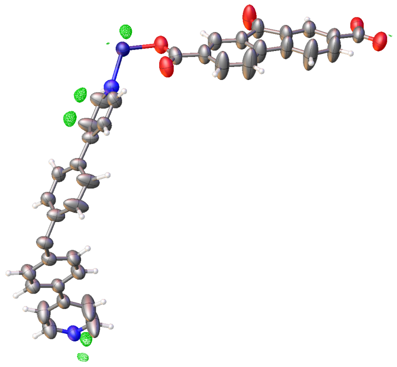

(h) DiSCaMB-HAR, level: ±0.5 e/Å^3

Figure S 17: Residual density for HACNIL obtained with various refinement techniques.

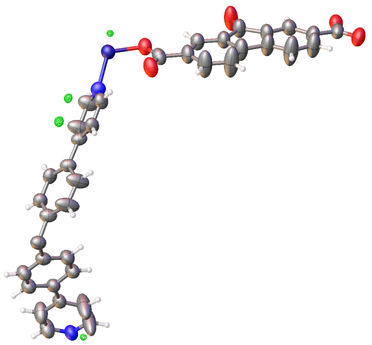

(a) IAM, level: ±0.6 e/Å^3

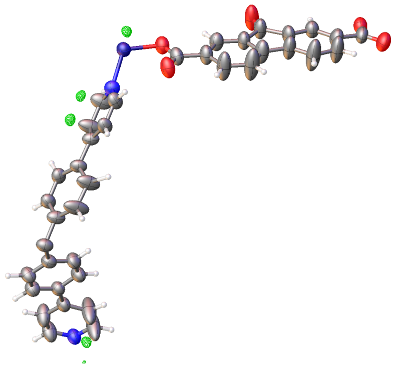

(b) DiSCaMB-HAR, level: ±0.6 e/Å^3

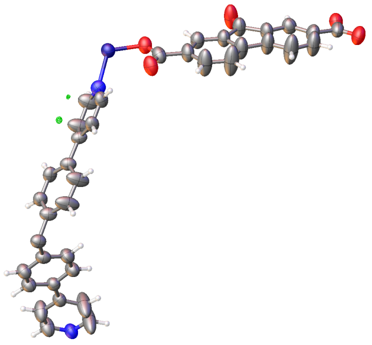

(c) IAM, level: ±0.7 e/Å^3

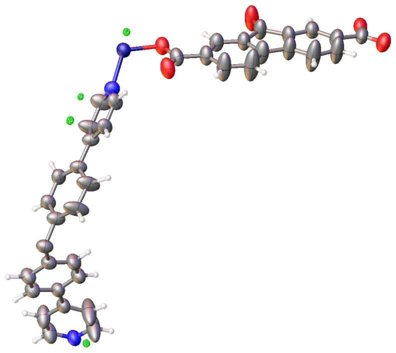

(d) DiSCaMB-HAR, level: ±0.7 e/Å^3

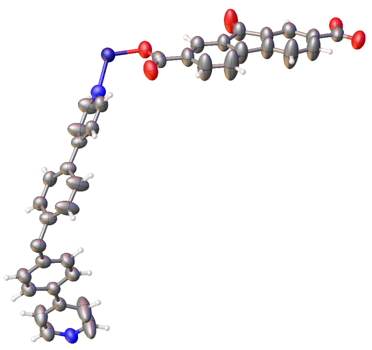

(e) IAM, level: ±0.8 e/Å^3

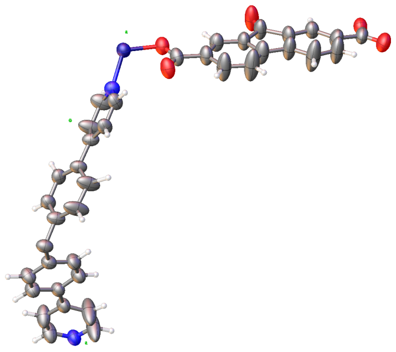

(f) DiSCaMB-HAR, level: ±0.8 e/Å^3

Figure S 18: Residual density for HACNIL obtained with various refinement techniques.

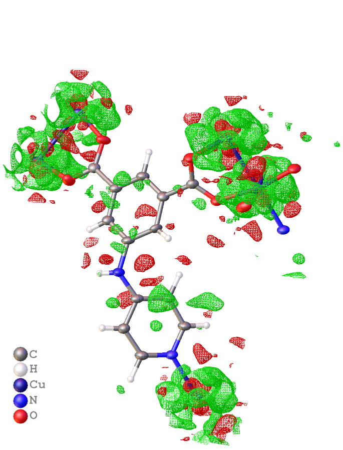

(a) IAM, level: ±0.2 e/Å^3

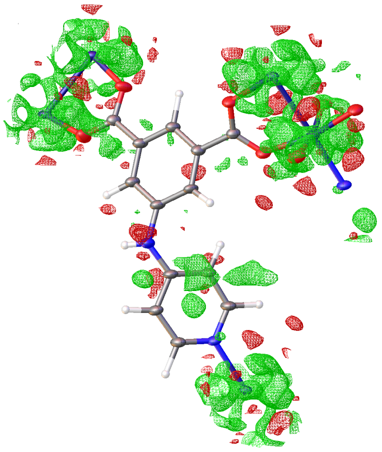

(b) DiSCaMB-HAR, level: ±0.2 e/Å^3

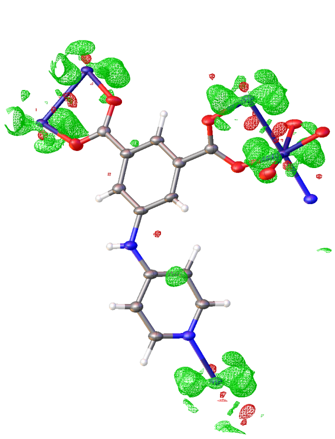

(c) IAM, level: ±0.3 e/Å^3

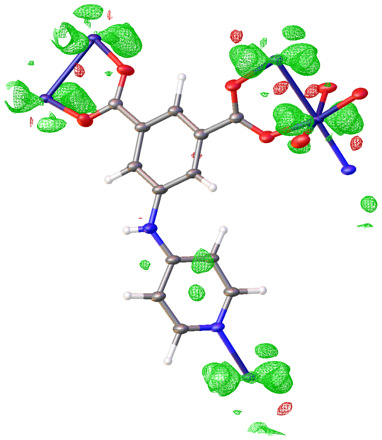

(d) DiSCaMB-HAR, level: ±0.3 e/Å^3

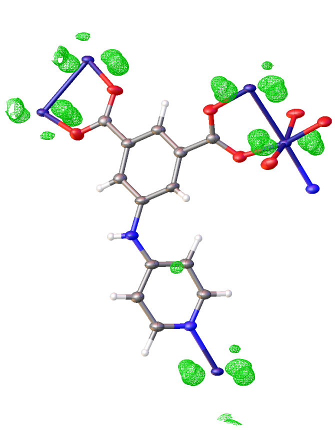

(e) IAM, level: ±0.4 e/Å^3

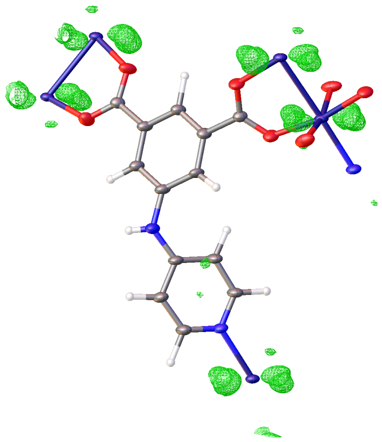

(f) DiSCaMB-HAR, level: ±0.4 e/Å^3

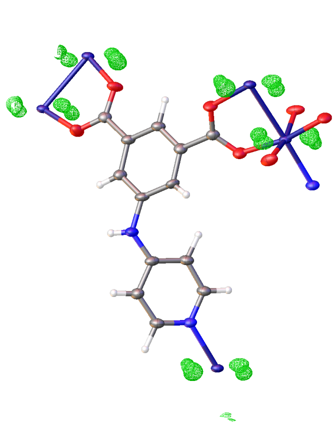

(g) IAM, level: ±0.5 e/Å^3

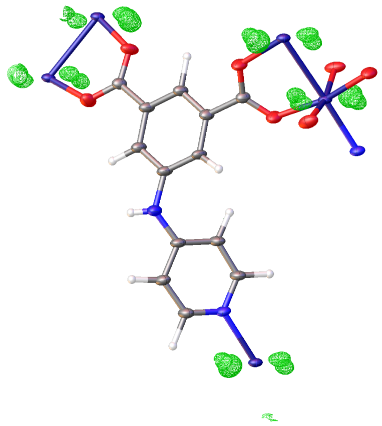

(h) DiSCaMB-HAR, level: ±0.5 e/Å^3

Figure S 19: Residual density for HOYKUD obtained with various refinement techniques.

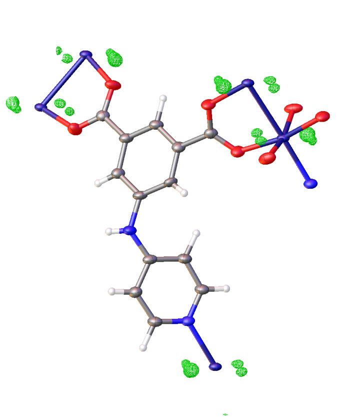

(a) IAM, level: ±0.6 e/Å^3

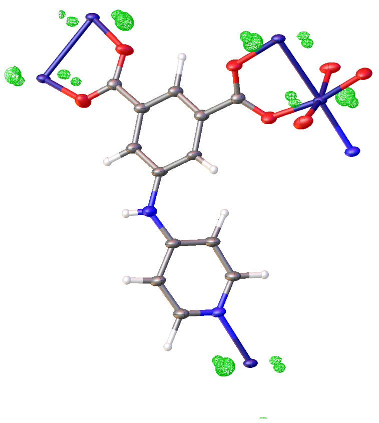

(b) DiSCaMB-HAR, level: ±0.6 e/Å^3

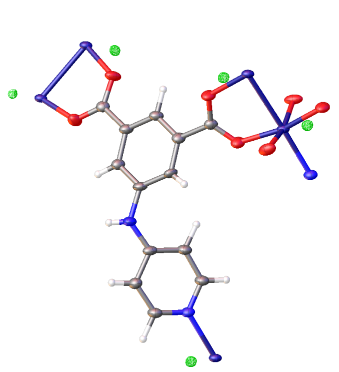

(c) IAM, level: ±0.7 e/Å^3

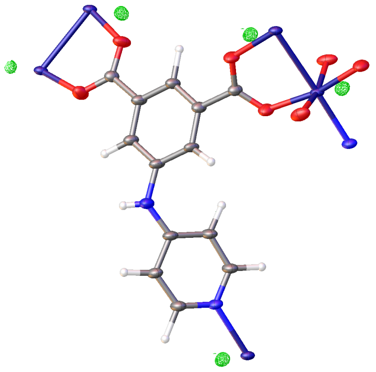

(d) DiSCaMB-HAR, level: ±0.7 e/Å^3

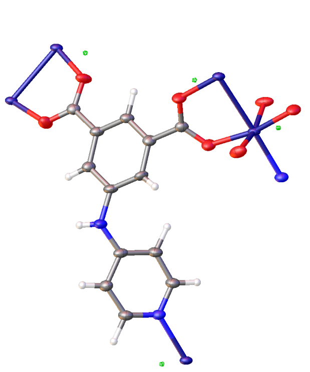

(e) IAM, level: ±0.8 e/Å^3

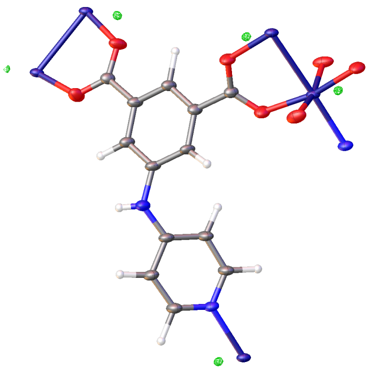

(f) DiSCaMB-HAR, level: ±0.8 e/Å^3

Figure S 20: Residual density for HOYKUD obtained with various refinement techniques.

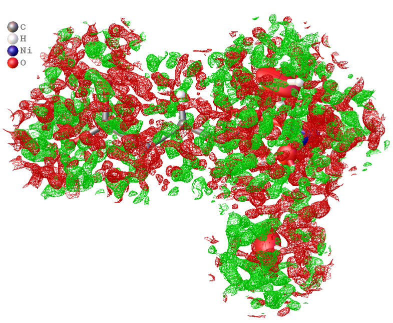

(a) IAM, level: ±0.5 e/Å^3

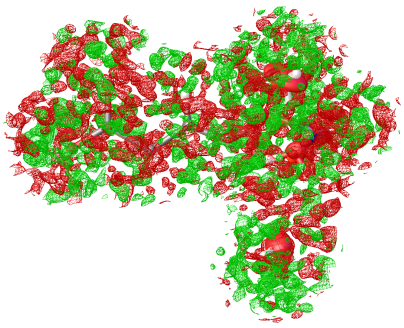

(b) DiSCaMB-HAR, level: ±0.5 e/Å^3

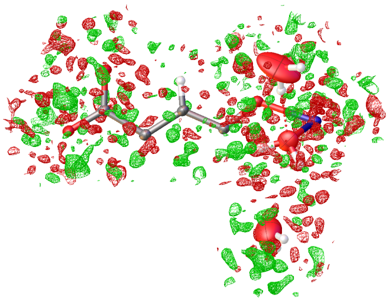

(c) IAM, level: ±0.7 e/Å^3

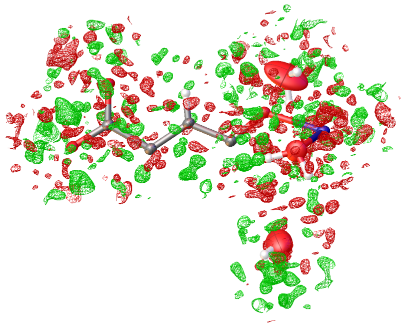

(d) DiSCaMB-HAR, level: ±0.7 e/Å^3

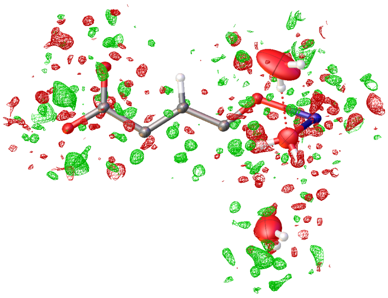

(e) IAM, level: ±0.8 e/Å^3

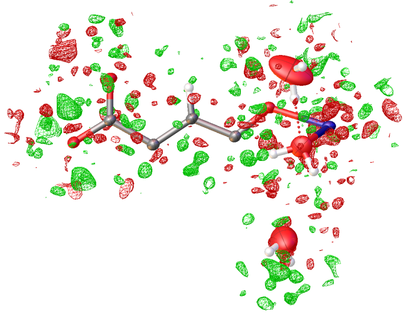

(f) DiSCaMB-HAR, level: ±0.8 e/Å^3

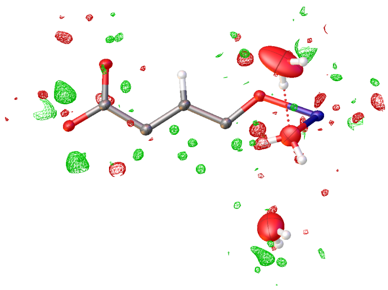

(g) IAM, level: ±1.0 e/Å^3

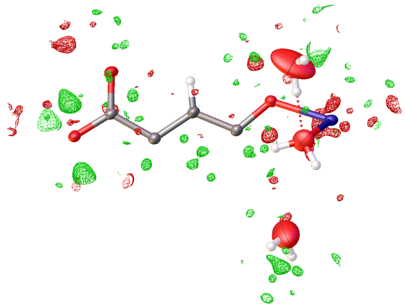

(h) DiSCaMB-HAR, level: ±1.0 e/Å^3

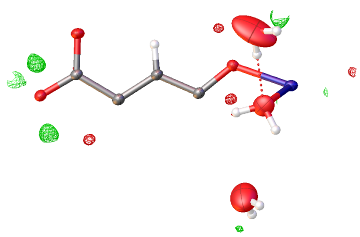

(i) IAM, level: ±1.5 e/Å^3

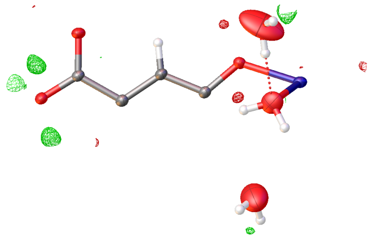

(j) DiSCaMB-HAR, level: ±1.5 e/Å^3

Figure S 21: Residual density for PODWIS obtained with various refinement techniques.

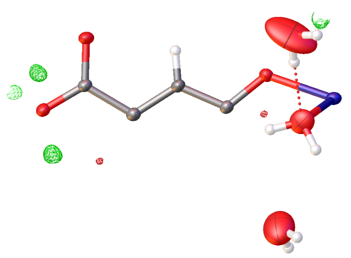

(a) IAM, level: ±2.0 e/Å^3

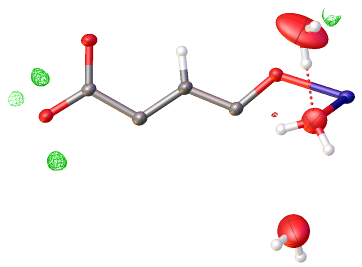

(b) DiSCaMB-HAR, level: ±2.0 e/Å^3

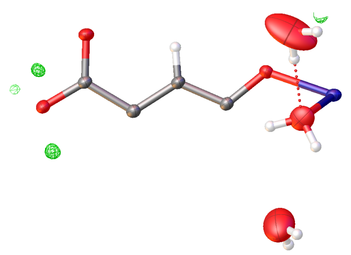

(c) IAM, level: ±2.5 e/Å^3

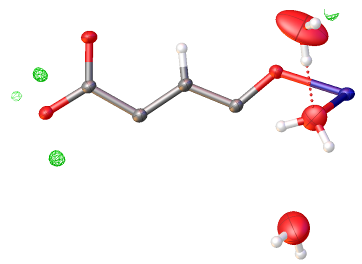

(d) DiSCaMB-HAR, level: ±2.5 e/Å^3

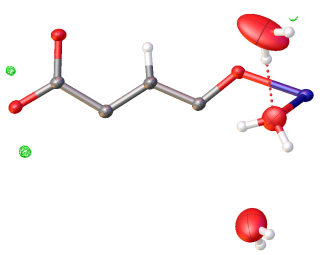

(e) IAM, level: ±3.0 e/Å^3

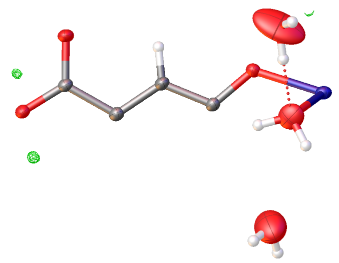

(f) DiSCaMB-HAR, level: ±3.0 e/Å^3

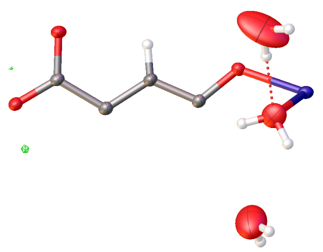

(g) IAM, level: ±3.5 e/Å^3

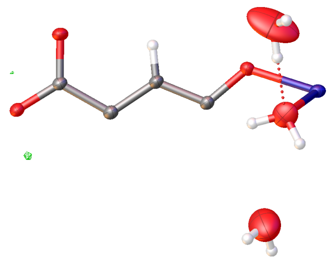

(h) DiSCaMB-HAR, level: ±3.5 e/Å^3

Figure S 22: Residual density for PODWIS obtained with various refinement techniques.

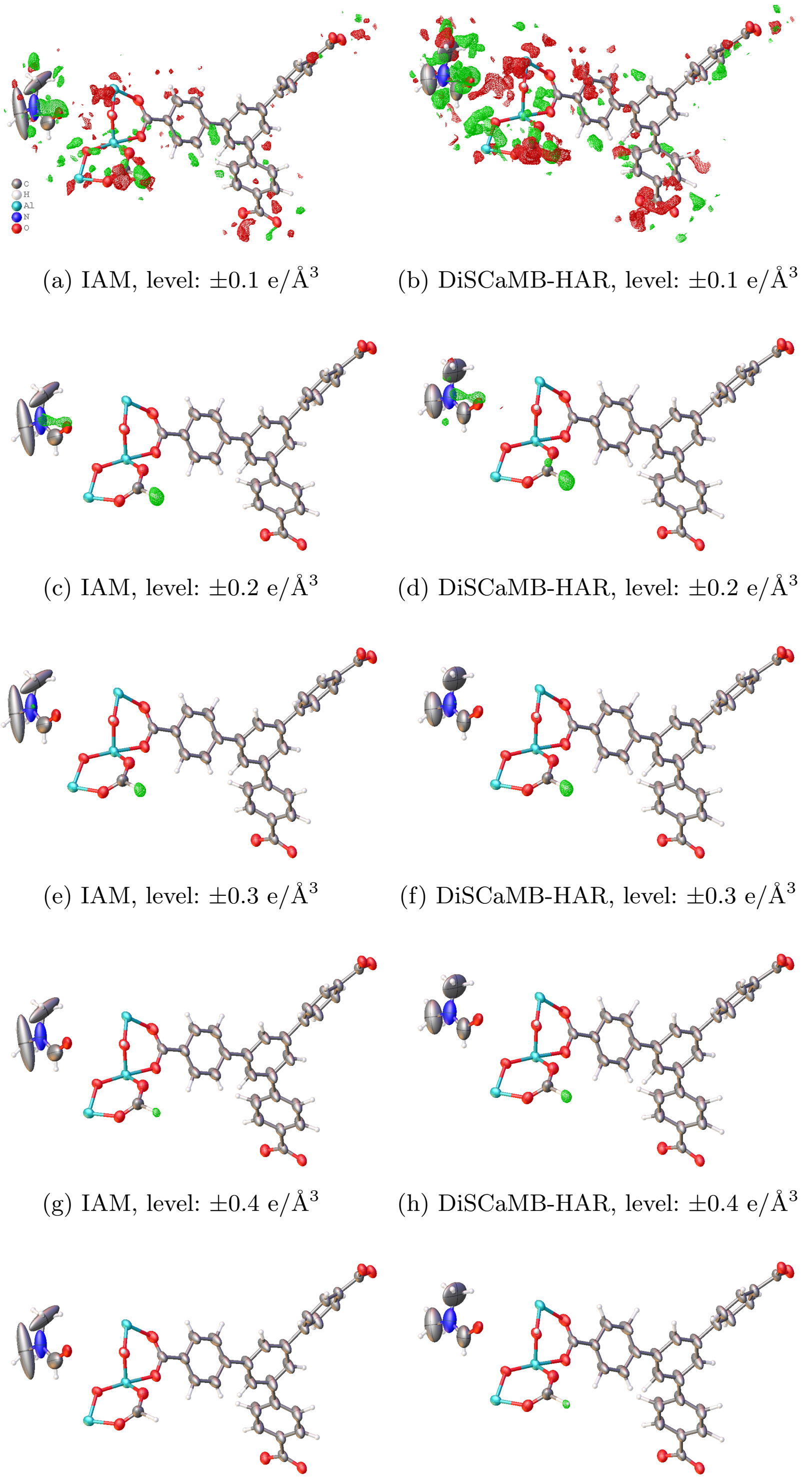

(a) IAM, level: ±0.1 e/Å^3 (b) DiSCaMB-HAR, level: ±0.1 e/Å^3

(c) IAM, level: ±0.2 e/Å^3 (d) DiSCaMB-HAR, level: ±0.2 e/Å^3

(e) IAM, level: ±0.3 e/Å^3 (f) DiSCaMB-HAR, level: ±0.3 e/Å^3

(g) IAM, level: ±0.4 e/Å^3 (h) DiSCaMB-HAR, level: ±0.4 e/Å^3

(i) IAM, level: ±0.5 e/Å^3 (j) DiSCaMB-HAR, level: ±0.5 e/Å^3

Figure S 23: Residual density for QOXVEH obtained with various refinement techniques.

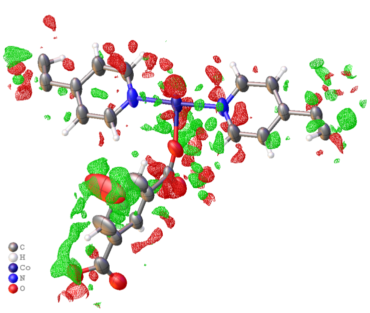

(a) IAM, level: ±0.2 e/Å^3

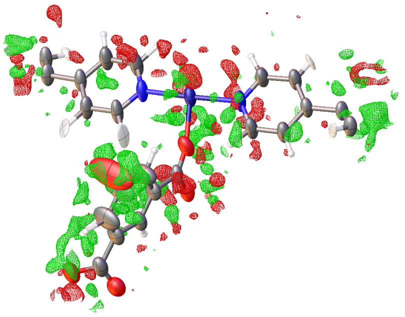

(b) DiSCaMB-HAR, level: ±0.2 e/Å^3

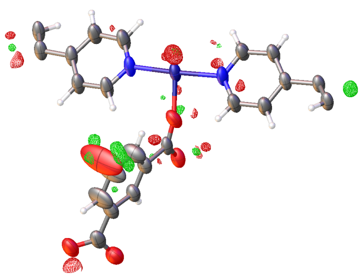

(c) IAM, level: ±0.3 e/Å^3

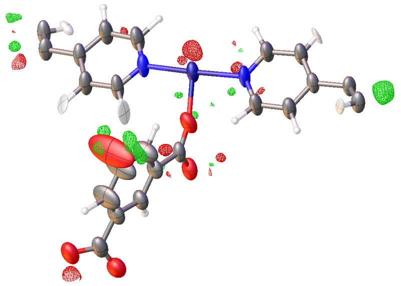

(d) DiSCaMB-HAR, level: ±0.3 e/Å^3

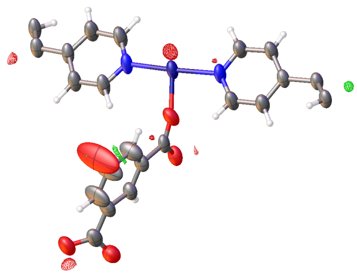

(e) IAM, level: ±0.4 e/Å^3

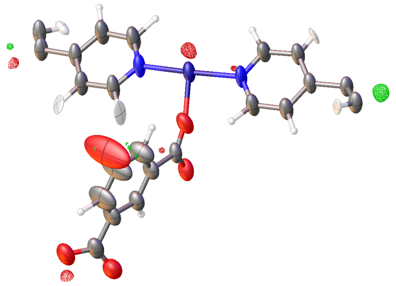

(f) DiSCaMB-HAR, level: ±0.4 e/Å^3

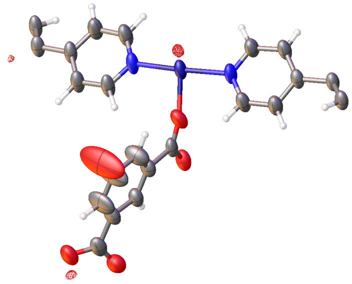

(g) IAM, level: ±0.5 e/Å^3

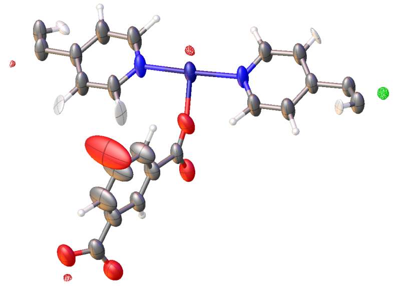

(h) DiSCaMB-HAR, level: ±0.5 e/Å^3

Figure S 24: Residual density for RALFIX obtained with various refinement techniques.

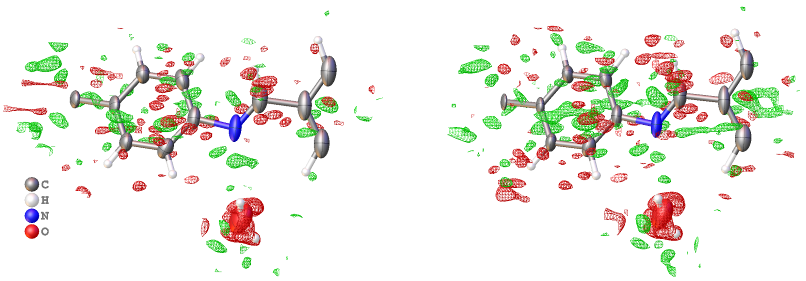

(a) IAM, level: ±0.3 e/Å^3 (b) DiSCaMB-HAR, level: ±0.3 e/Å^3

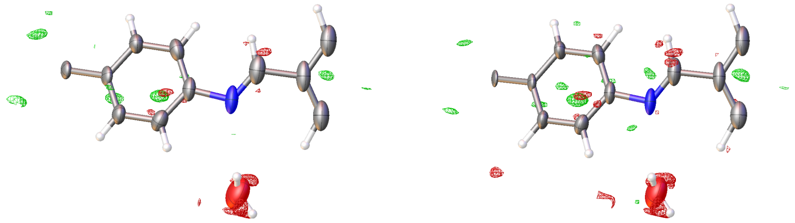

(c) IAM, level: ±0.4 e/Å^3 (d) DiSCaMB-HAR, level: ±0.4 e/Å^3

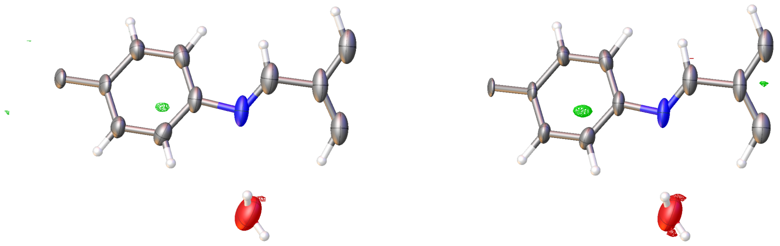

(e) IAM, level: ±0.5 e/Å^3 (f) DiSCaMB-HAR, level: ±0.5 e/Å^3

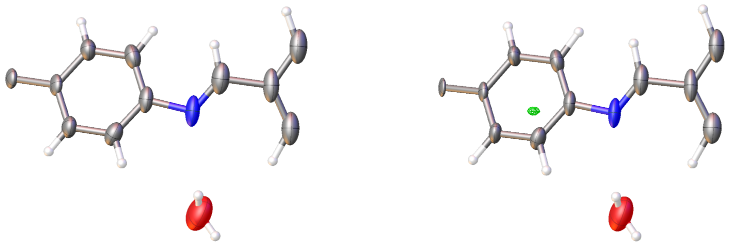

(g) IAM, level: ±0.6 e/Å^3 (h) DiSCaMB-HAR, level: ±0.6 e/Å^3

Figure S 25: Residual density for RIHYIT obtained with various refinement techniques.

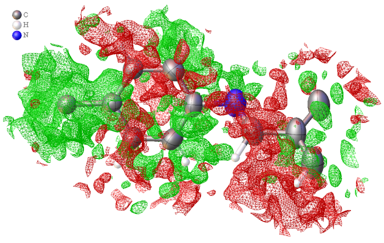

(a) IAM, level: ±0.2 e/Å^3

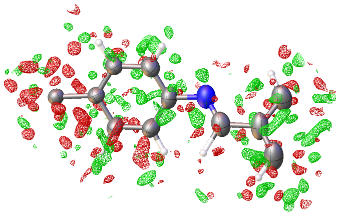

(b) DiSCaMB-HAR, level: ±0.2 e/Å^3

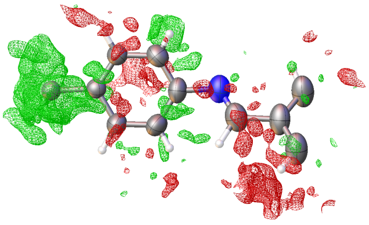

(c) IAM, level: ±0.3 e/Å^3

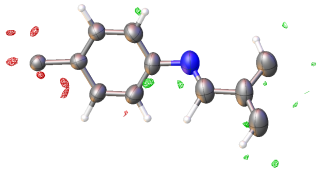

(d) DiSCaMB-HAR, level: ±0.3 e/Å^3

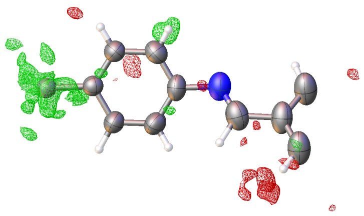

(e) IAM, level: ±0.4 e/Å^3

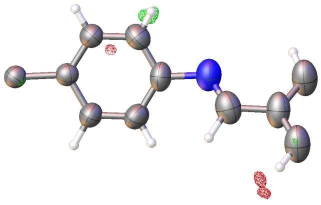

(f) IAM, level: ±0.5 e/Å^3

Figure S 26: Residual density for RIHYOZ01 obtained with various refinement techniques.

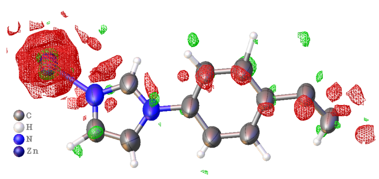

(a) IAM, level: ±0.2 e/Å^3

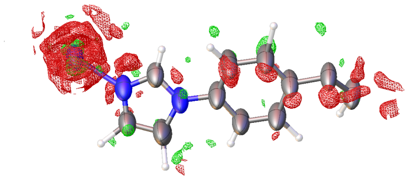

(b) DiSCaMB-HAR, level: ±0.2 e/Å^3

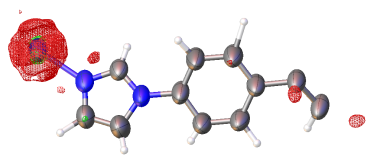

(c) IAM, level: ±0.3 e/Å^3

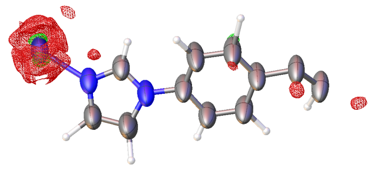

(d) DiSCaMB-HAR, level: ±0.3 e/Å^3

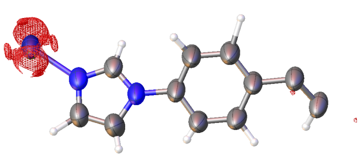

(e) IAM, level: ±0.4 e/Å^3

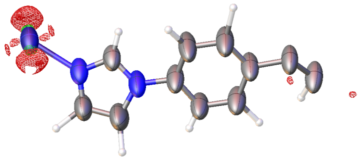

(f) DiSCaMB-HAR, level: ±0.4 e/Å^3

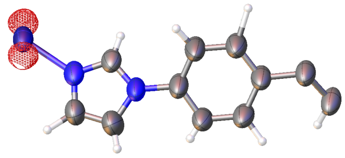

(g) IAM, level: ±0.5 e/Å^3

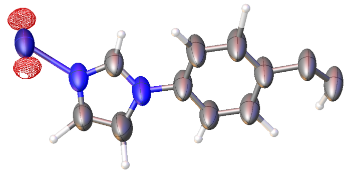

(h) DiSCaMB-HAR, level: ±0.5 e/Å^3

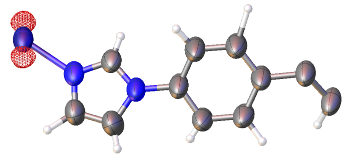

(i) IAM, level: ±0.6 e/Å^3

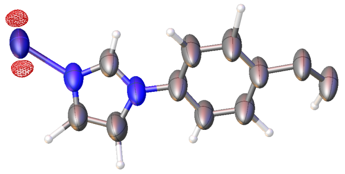

(j) DiSCaMB-HAR, level: ±0.6 e/Å^3

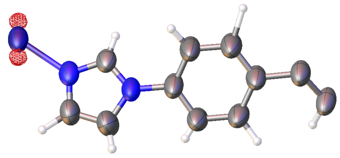

(k) IAM, level: ±0.7 e/Å^3

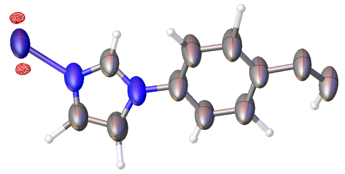

(l) DiSCaMB-HAR, level: ±0.7 e/Å^3

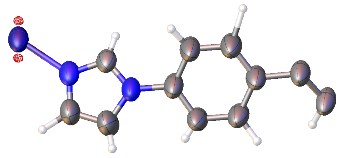

(m) IAM, level: ±0.8 e/Å^3

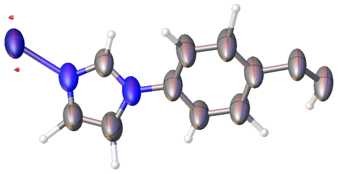

(n) DiSCaMB-HAR, level: ±0.8 e/Å^3

Figure S 27: Residual density for UYETIE obtained with various refinement techniques.

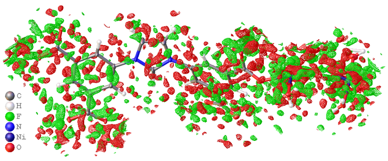

(a) IAM, level: ± 0.3 e/Å^3

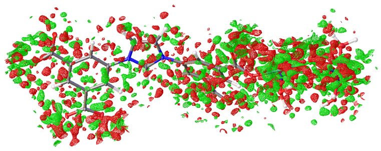

(b) DiSCaMB-HAR, level: ± 0.3 e/Å^3

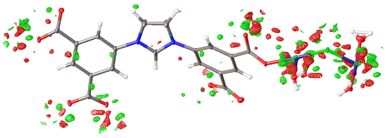

(c) IAM, level: ± 0.5 e/Å^3

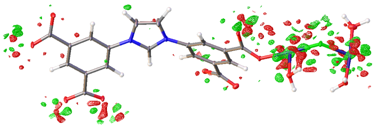

(d) DiSCaMB-HAR, level: ± 0.5 e/Å^3

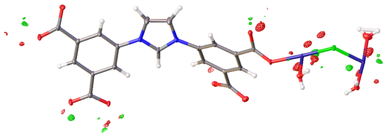

(e) IAM, level: ± 0.7 e/Å^3

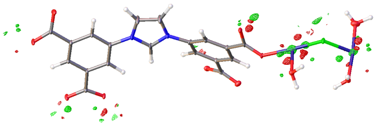

(f) DiSCaMB-HAR, level: ± 0.7 e/Å^3

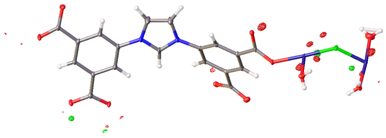

(g) IAM, level: ± 0.8 e/Å^3

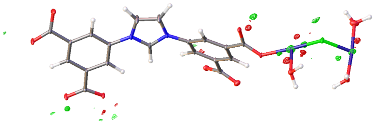

(h) DiSCaMB-HAR, level: ± 0.8 e/Å^3

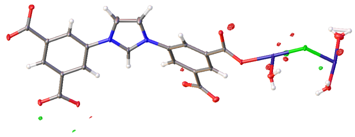

(i) IAM, level: ± 0.9 e/Å^3

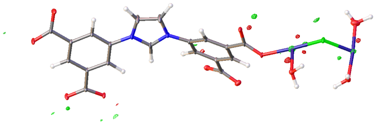

(j) DiSCaMB-HAR, level: ± 0.9 e/Å^3

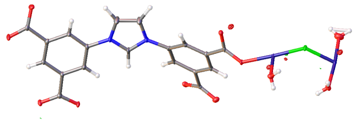

(k) IAM, level: ± 1.0 e/Å^3

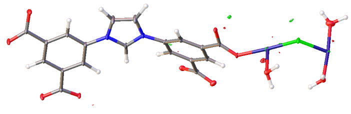

(l) DiSCaMB-HAR, level: ± 1.0 e/Å^3

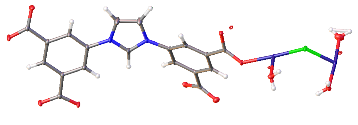

(m) IAM, level: ± 1.1 e/Å^3

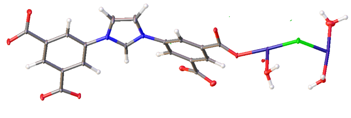

(n) DiSCaMB-HAR, level: ± 1.1 e/Å^3

Figure S 28: Residual density for VOCYEV obtained with various refinement techniques.

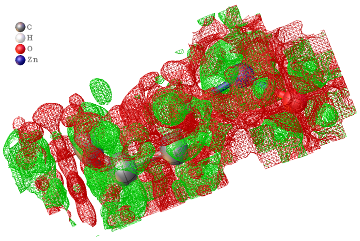

(a) IAM, level: ±0.2 e/Å^3

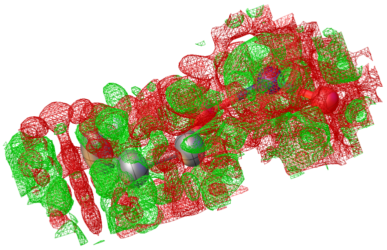

(b) DiSCaMB-HAR, level: ±0.2 e/Å^3

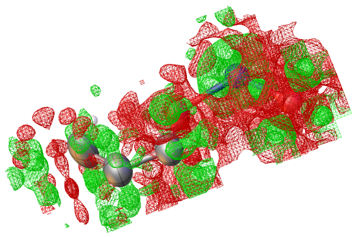

(c) IAM, level: ±0.3 e/Å^3

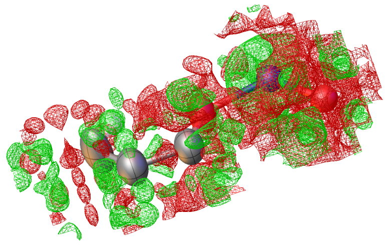

(d) DiSCaMB-HAR, level: ±0.3 e/Å^3

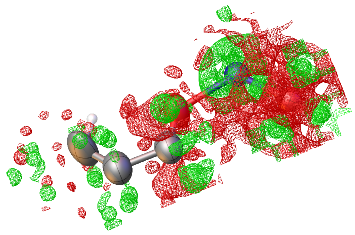

(e) IAM, level: ±0.4 e/Å^3

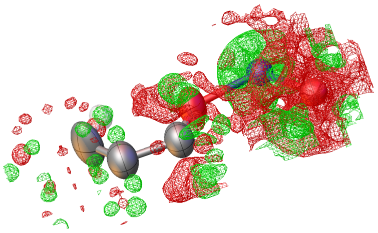

(f) DiSCaMB-HAR, level: ±0.4 e/Å^3

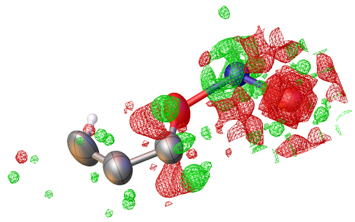

(g) IAM, level: ±0.5 e/Å^3

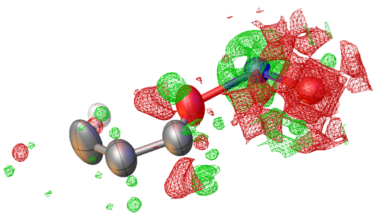

(h) DiSCaMB-HAR, level: ±0.5 e/Å^3

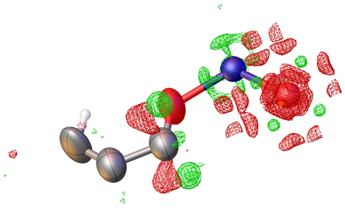

(i) IAM, level: ±0.6 e/Å^3

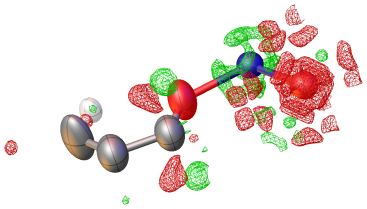

(j) DiSCaMB-HAR, level: ±0.6 e/Å^3

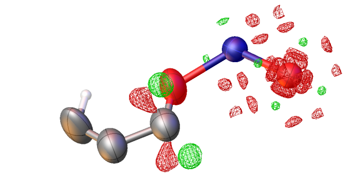

(k) IAM, level: ±0.7 e/Å^3

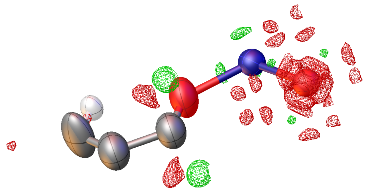

(l) DiSCaMB-HAR, level: ±0.7 e/Å^3

Figure S 29: Residual density for VUSKEA01 obtained with various refinement techniques.

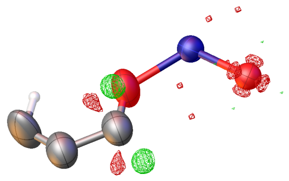

(a) IAM, level: ±0.8 e/Å^3

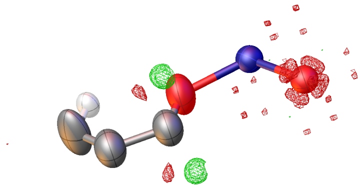

(b) DiSCaMB-HAR, level: ±0.8 e/Å^3

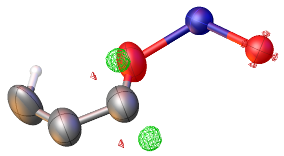

(c) IAM, level: ±0.9 e/Å^3

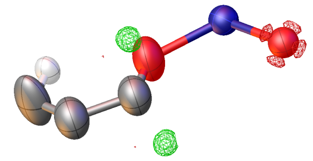

(d) DiSCaMB-HAR, level: ±0.9 e/Å^3

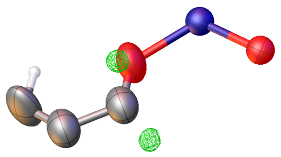

(e) IAM, level: ±1.0 e/Å^3

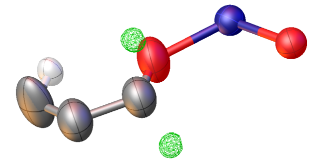

(f) DiSCaMB-HAR, level: ±1.0 e/Å^3

Figure S 30: Residual density for VUSKEA01 obtained with various refinement techniques.

2 Plots of F_{obs} vs. F_{calc}

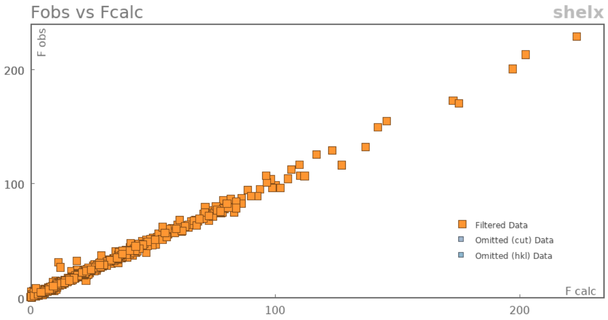

(a) HOYKUD, IAM

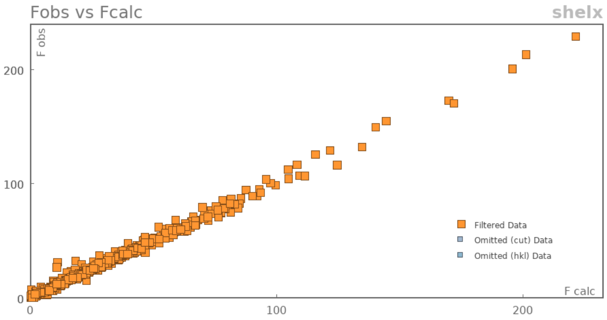

(b) HOYKUD, DiSCaMB-HAR

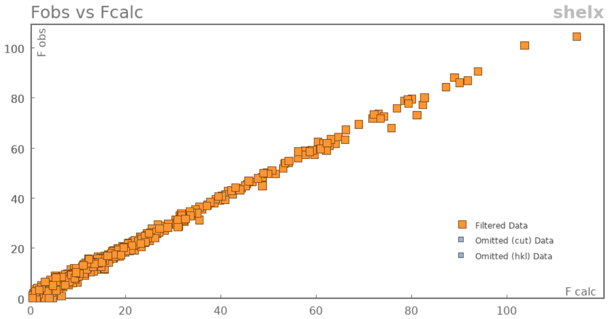

(c) CUGLYM08, IAM

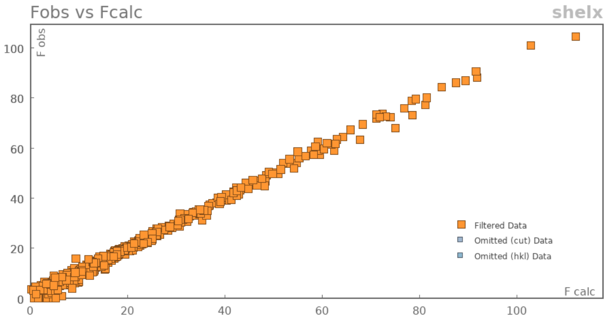

(d) CUGLYM08, DiSCaMB-HAR

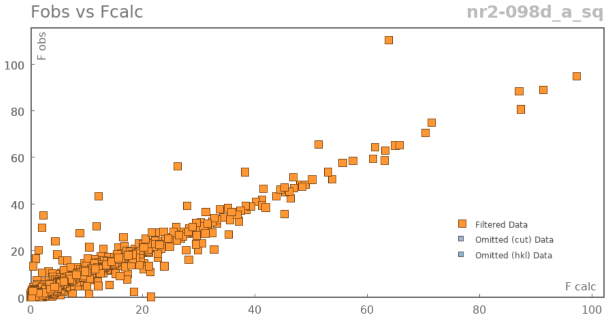

(e) HACNIL, IAM

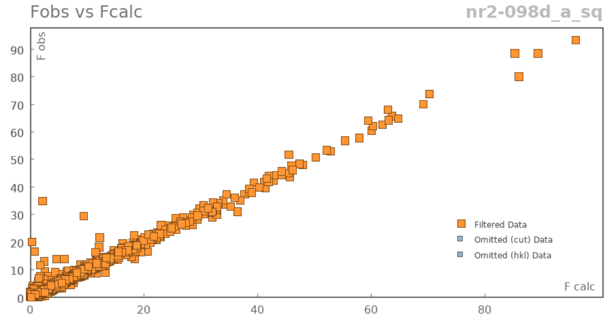

(f) HACNIL, DiSCaMB-HAR

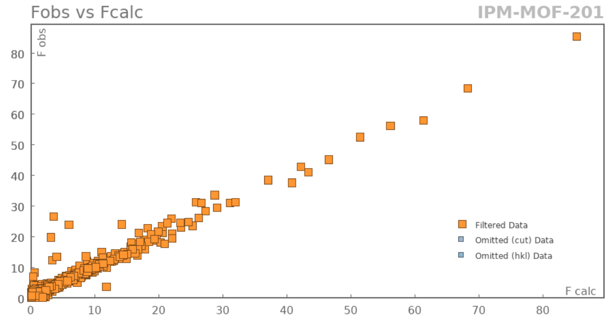

(g) GEYQEJ, IAM

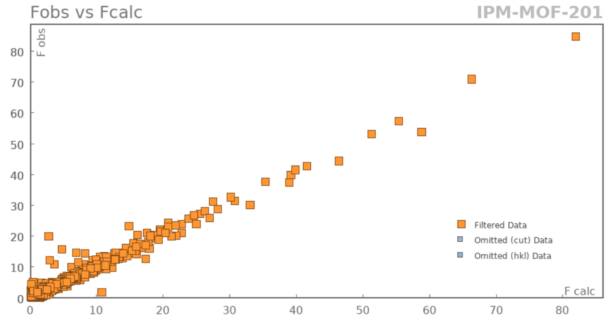

(h) GEYQEJ, DiSCaMB-HAR

Figure S 31: F_{obs} vs. F_{calc} plots.

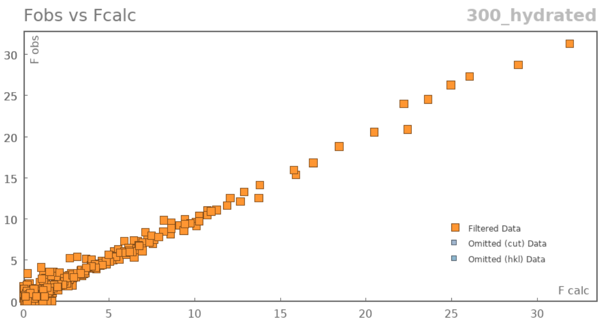

(a) RIHYIT, IAM

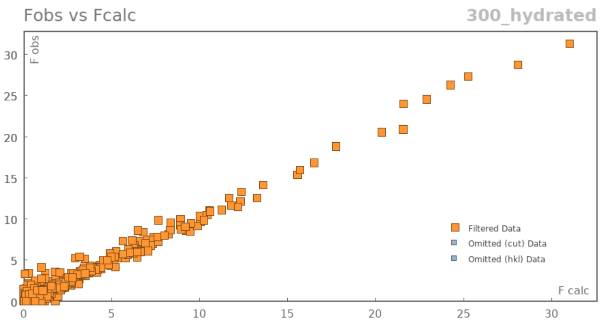

(b) RIHYIT, DiSCaMB-HAR

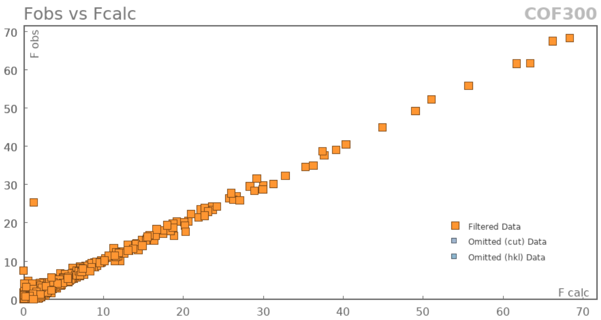

(c) RIHYOZ01, IAM

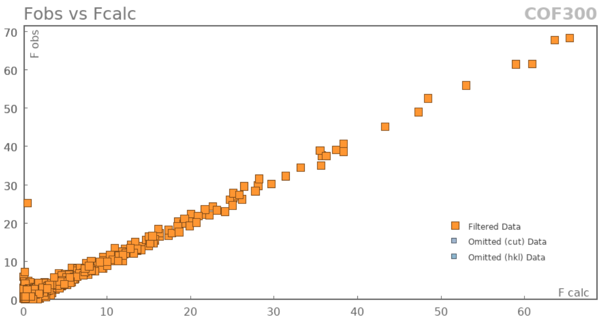

(d) RIHYOZ01, DiSCaMB-HAR

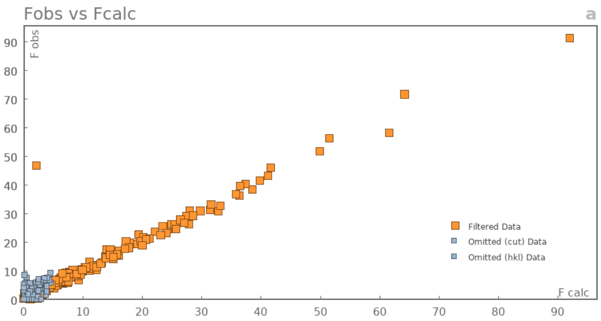

(e) VUSKEA01, IAM

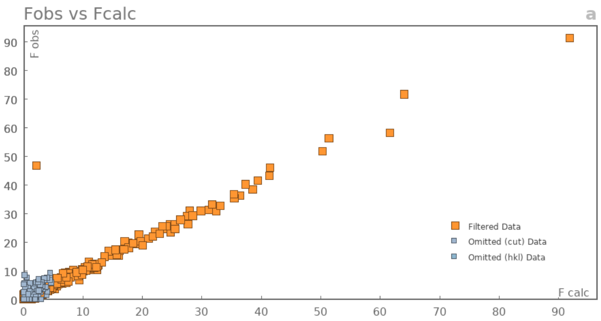

(f) VUSKEA01, DiSCaMB-HAR

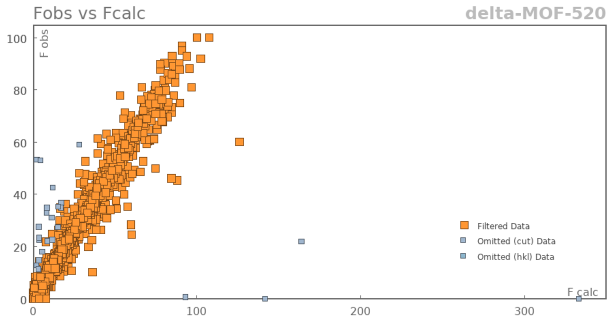

(g) QOXVEH, IAM

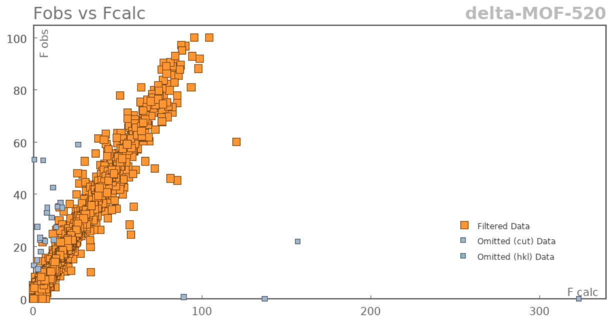

(h) QOXVEH, DiSCaMB-HAR

Figure S 32: F_{obs} vs. F_{calc} plots.

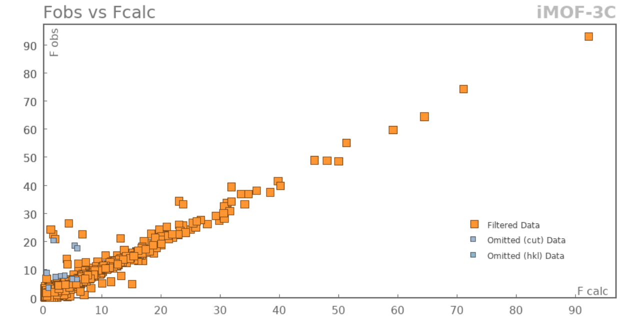

(a) ALUDOD, IAM

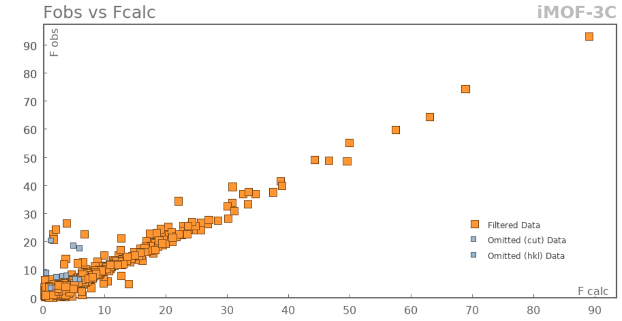

(b) ALUDOD, DiSCaMB-HAR

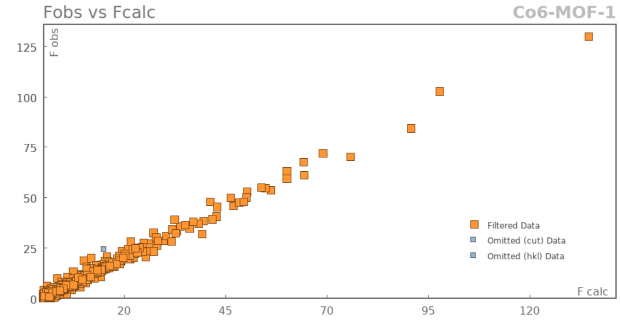

(c) FUVKEP, IAM

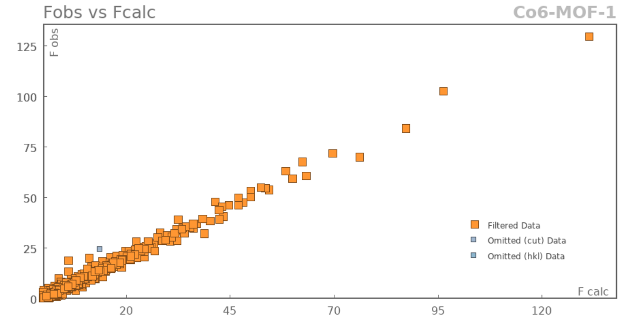

(d) FUVKEP, DiSCaMB-HAR

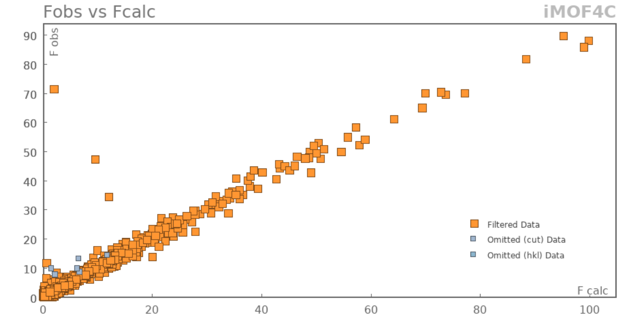

(e) UYETIE, IAM

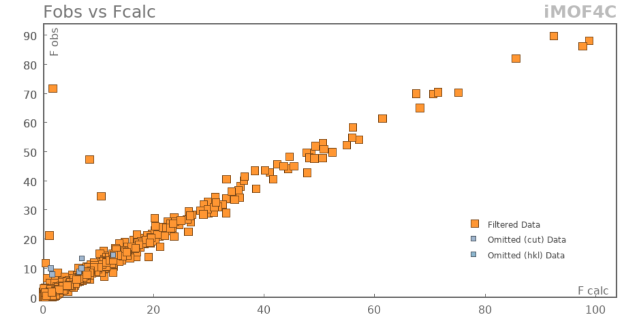

(f) UYETIE, DiSCaMB-HAR

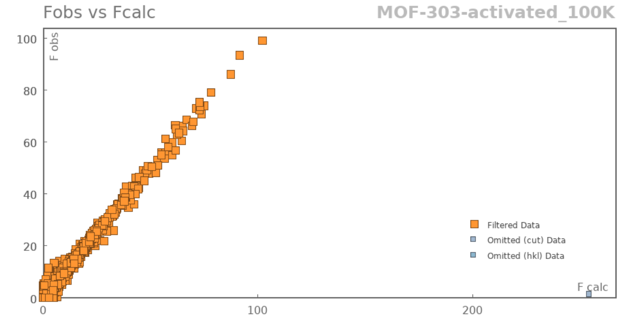

(g) CAMTET, IAM

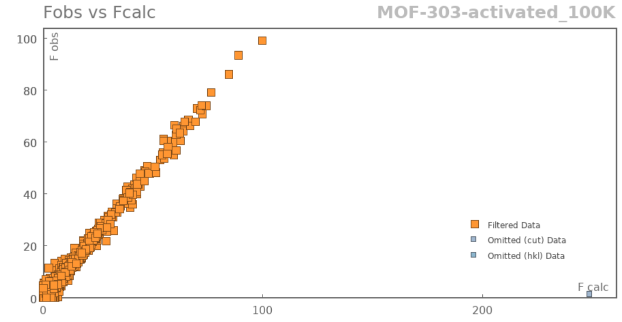

(h) CAMTET, DiSCaMB-HAR

Figure S 33: F_{obs} vs. F_{calc} plots.

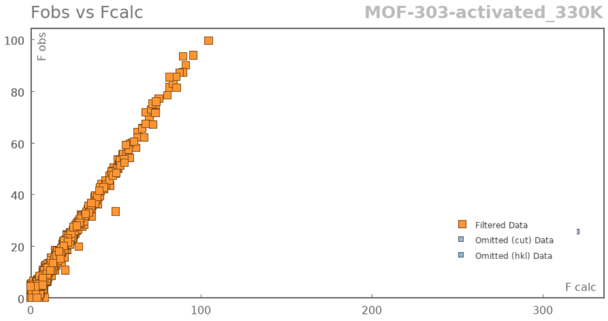

(a) CAMTET01, IAM

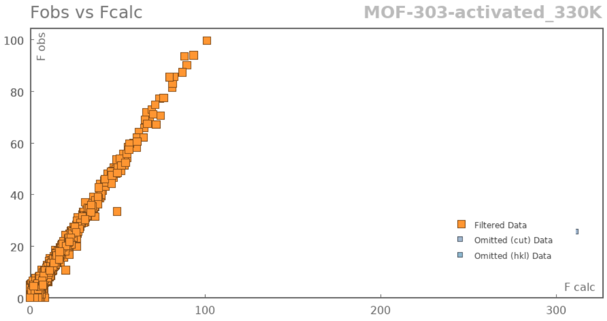

(b) CAMTET01, DiSCaMB-HAR

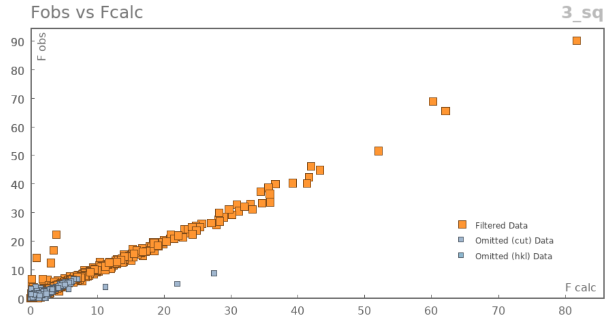

(c) RALFIX, IAM

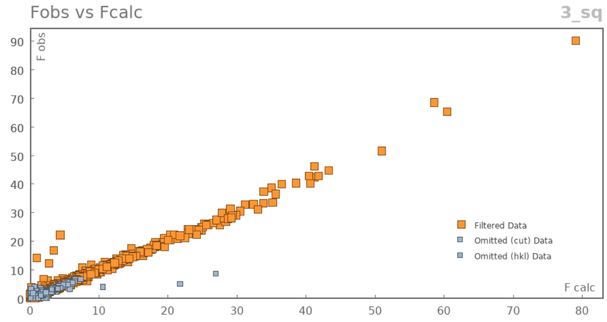

(d) RALFIX, DiSCaMB-HAR

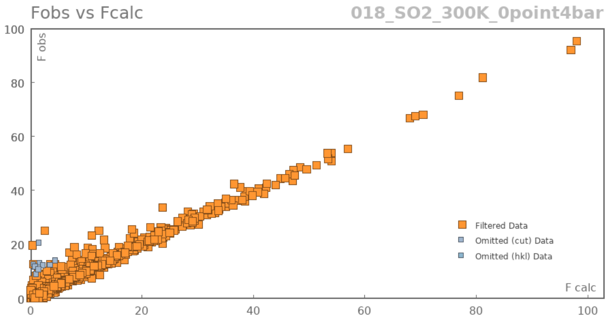

(e) PODWIS, IAM

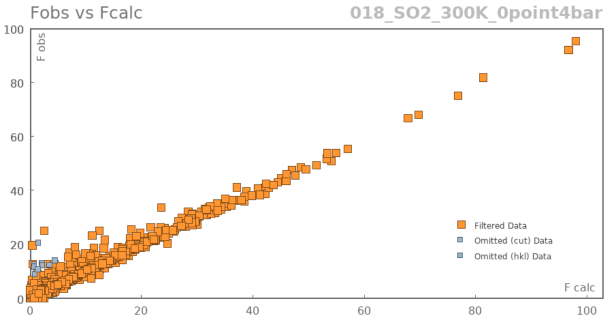

(f) PODWIS, DiSCaMB-HAR

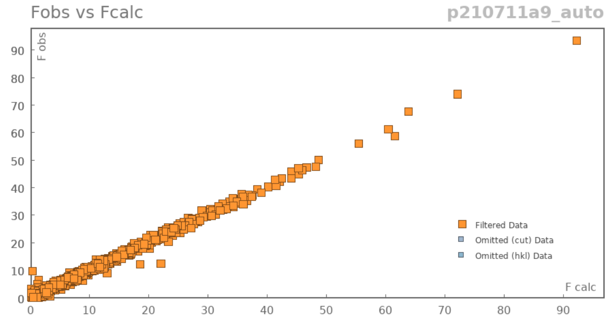

(g) VOCYEV, IAM

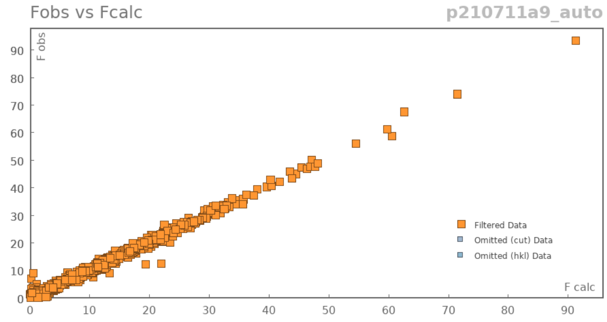

(h) VOCYEV, DiSCaMB-HAR

Figure S 34: F_{obs} vs. F_{calc} plots.

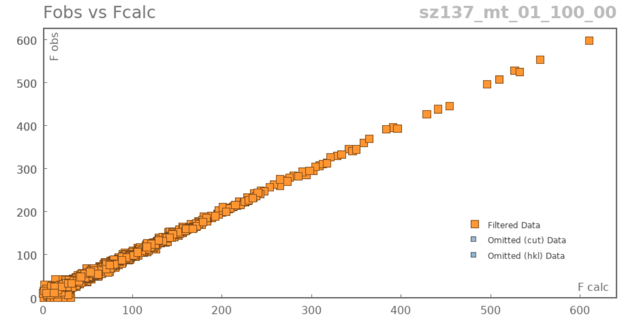

(a) SZ3-150K, IAM

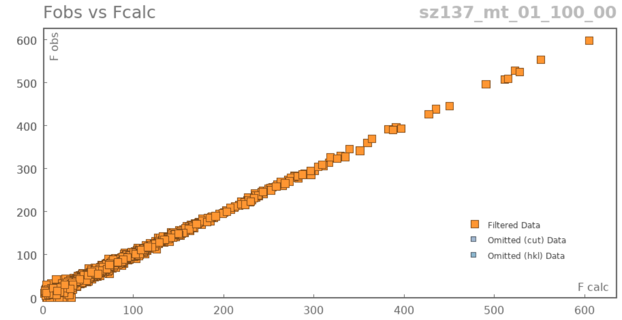

(b) SZ3-150K, DiSCaMB-HAR

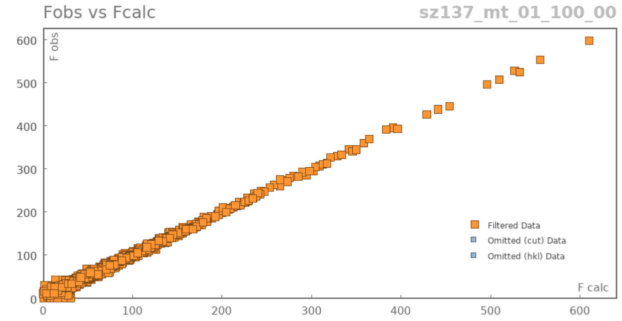

(c) SZ7-100K, IAM

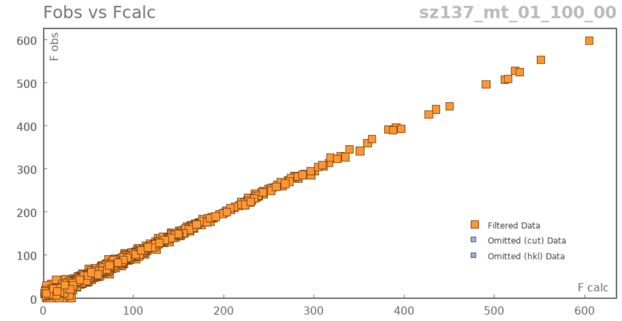

(d) SZ7-100K, DiSCaMB-HAR

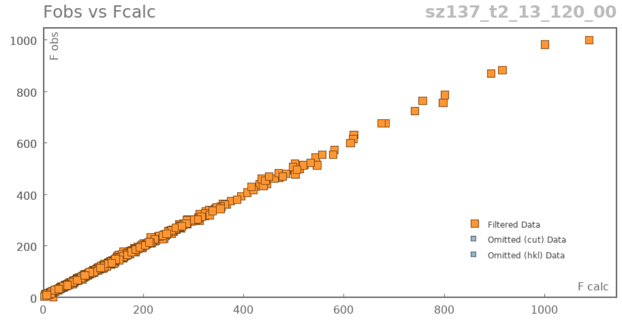

(e) SZ7-125K, IAM

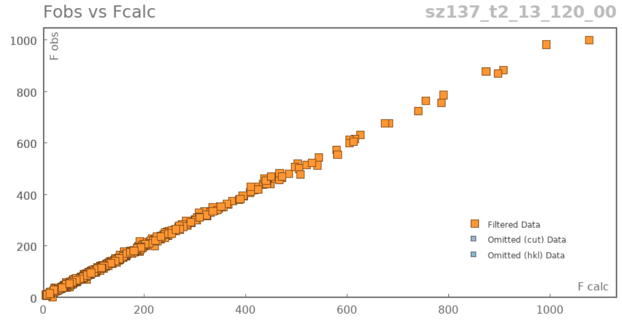

(f) SZ7-125K, DiSCaMB-HAR

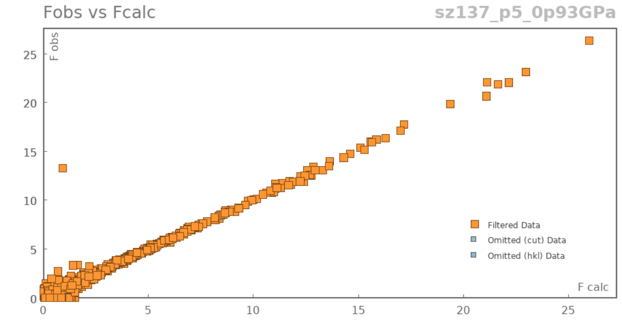

(g) SZ7-1GPa, IAM

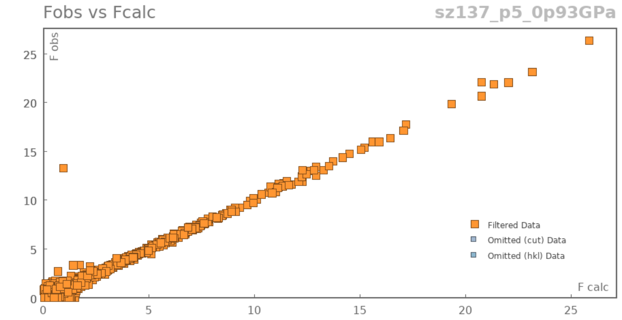

(h) SZ7-1GPa, DiSCaMB-HAR

Figure S 35: F_{obs} vs. F_{calc} plots.

3 Normal probability plots

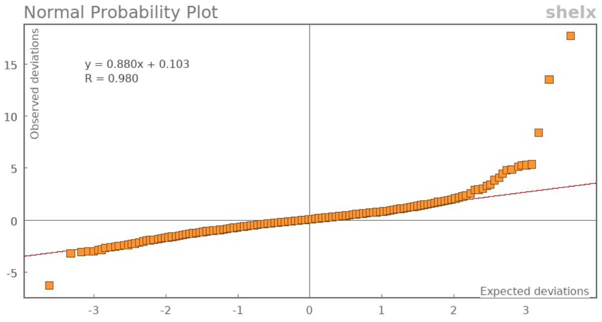

(a) HOYKUD, IAM

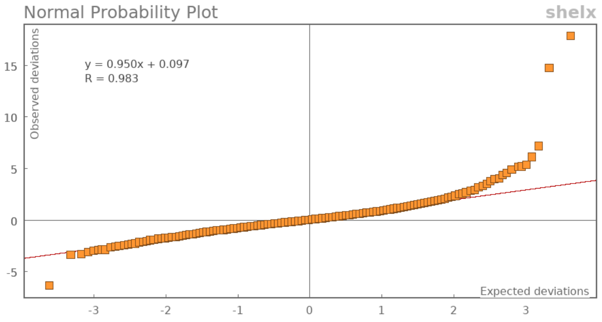

(b) HOYKUD, DiSCaMB-HAR

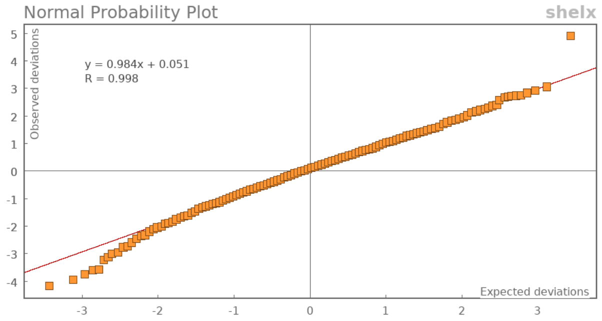

(c) CUGLYM08, IAM

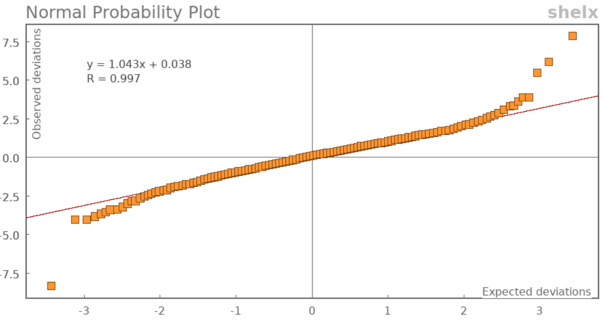

(d) CUGLYM08, DiSCaMB-HAR

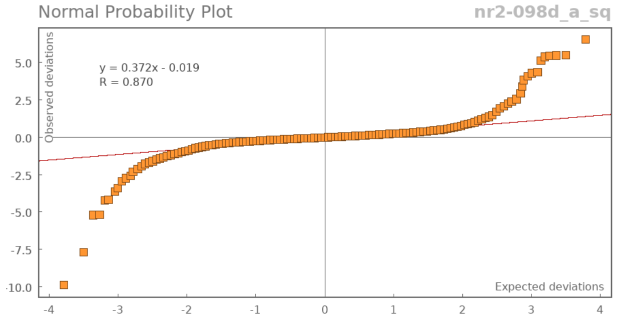

(e) HACNIL, IAM

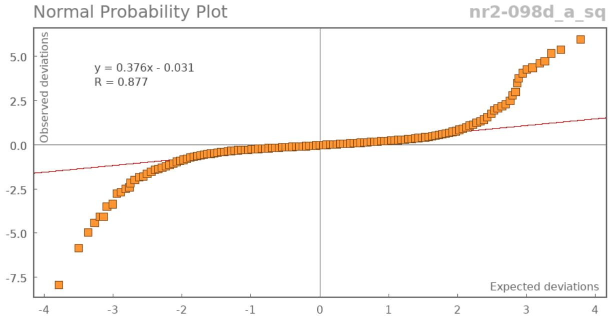

(f) HACNIL, DiSCaMB-HAR

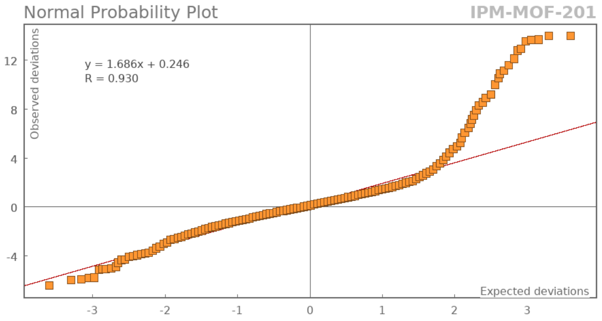

(g) GEYQEJ, IAM

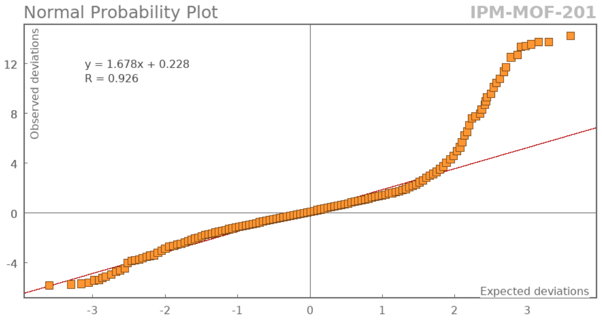

(h) GEYQEJ, DiSCaMB-HAR

Figure S 36: Normal probability plots plots.

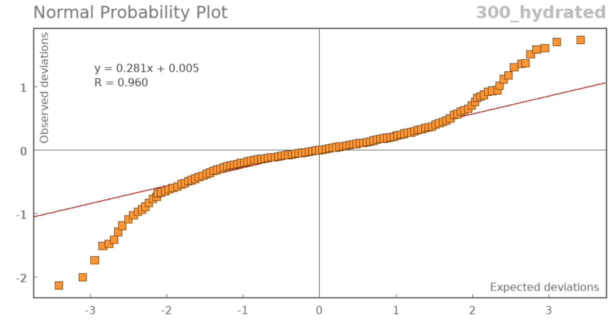

(a) RIHYIT, IAM

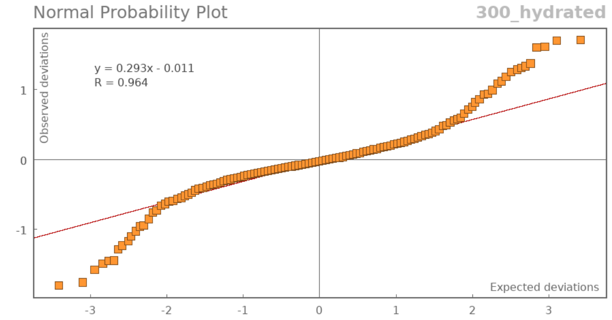

(b) RIHYIT, DiSCaMB-HAR

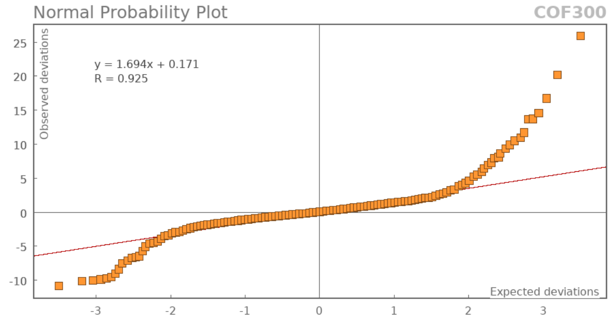

(c) RIHYOZ01, IAM

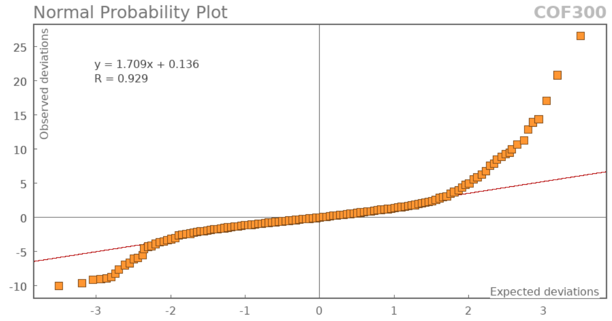

(d) RIHYOZ01, DiSCaMB-HAR

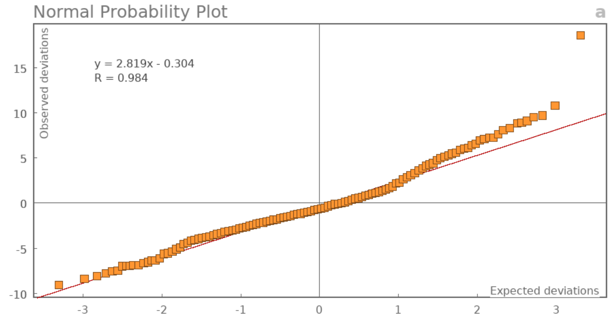

(e) VUSKEA01, IAM

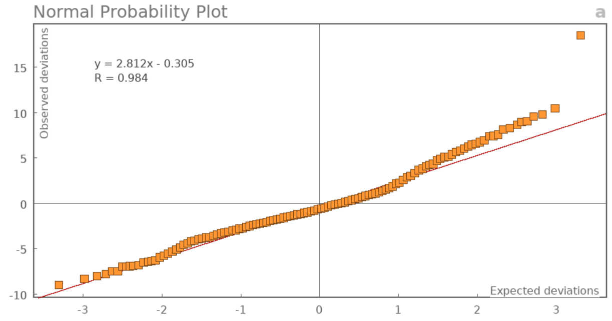

(f) VUSKEA01, DiSCaMB-HAR

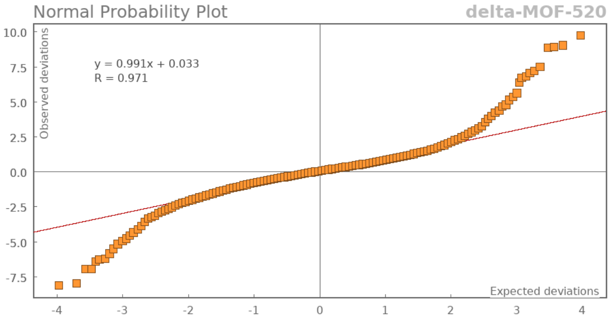

(g) QOXVEH, IAM

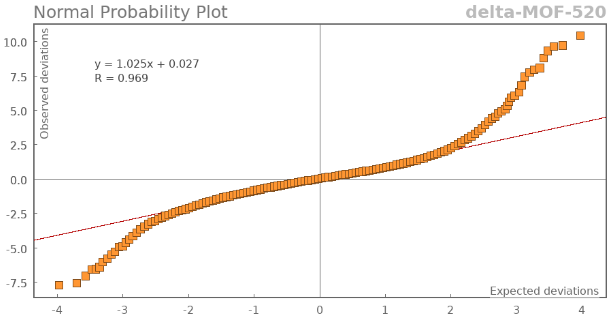

(h) QOXVEH, DiSCaMB-HAR

Figure S 37: Normal probability plots plots.

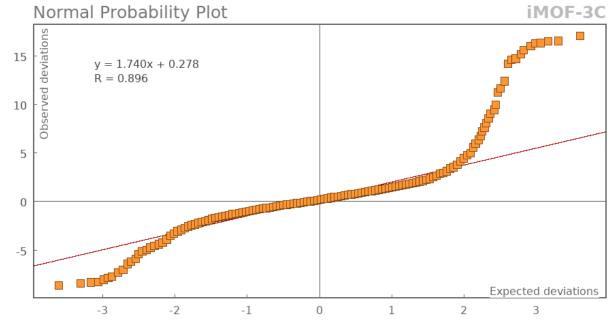

(a) ALUDOD, IAM

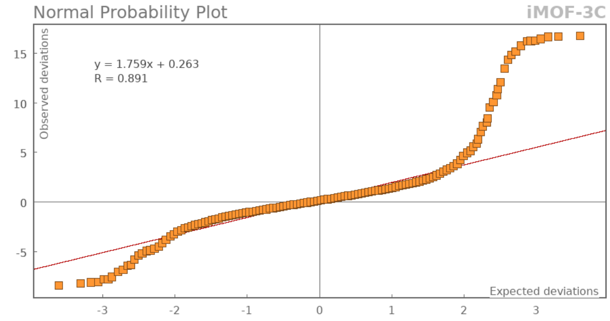

(b) ALUDOD, DiSCaMB-HAR

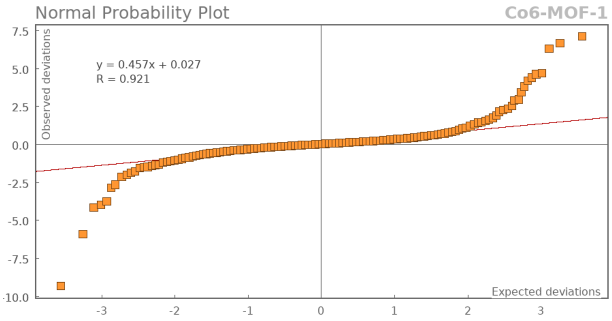

(c) FUVKEP, IAM

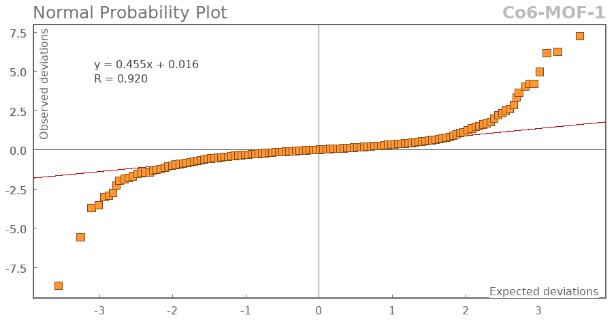

(d) FUVKEP, DiSCaMB-HAR

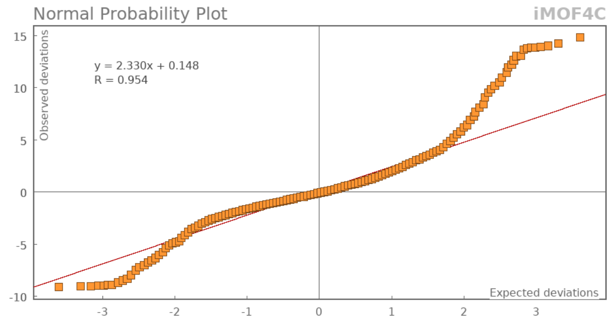

(e) UYETIE, IAM

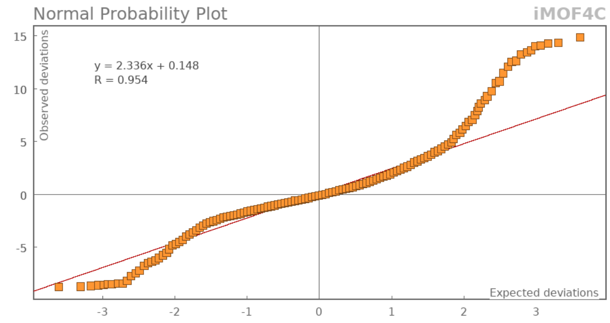

(f) UYETIE, DiSCaMB-HAR

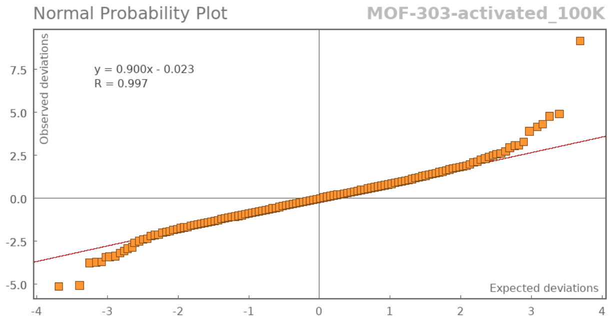

(g) CAMTET, IAM

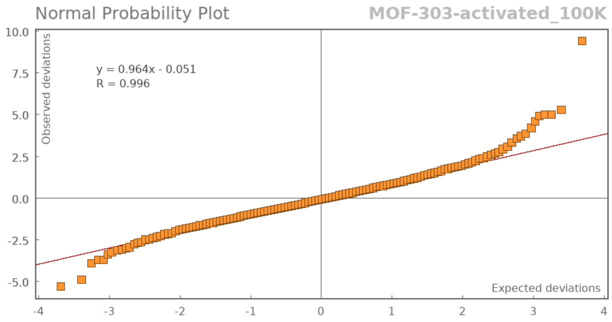

(h) CAMTET, DiSCaMB-HAR

Figure S 38: Normal probability plots plots.

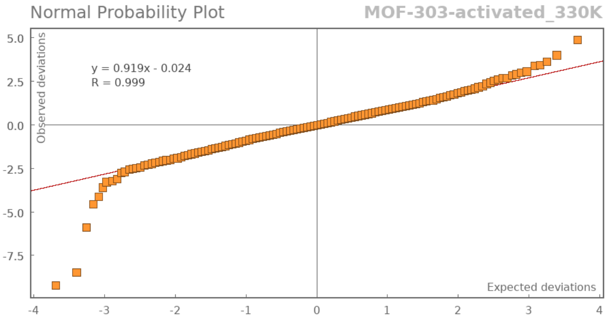

(a) CAMTET01, IAM

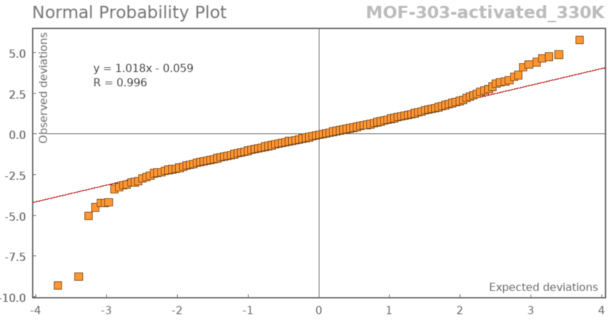

(b) CAMTET01, DiSCaMB-HAR

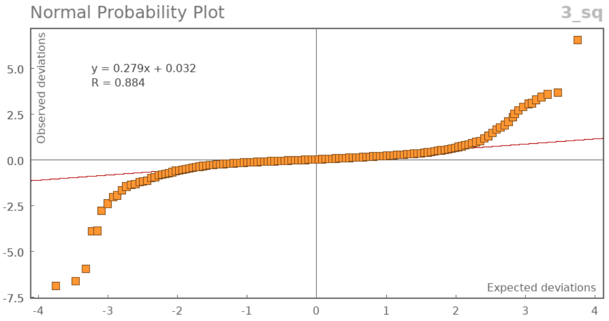

(c) RALFIX, IAM

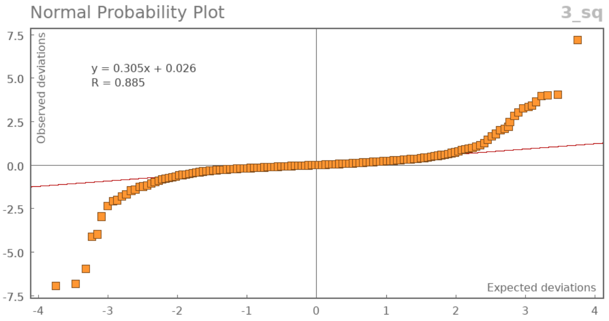

(d) RALFIX, DiSCaMB-HAR

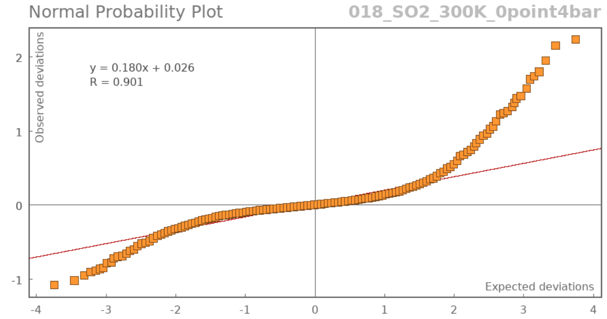

(e) PODWIS, IAM

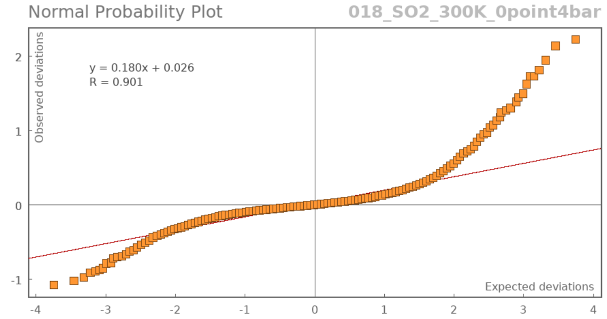

(f) PODWIS, DiSCaMB-HAR

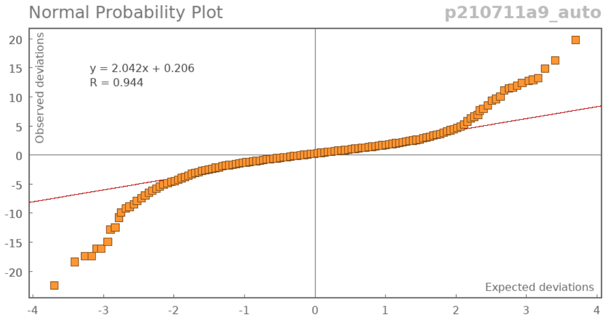

(g) VOCYEV, IAM

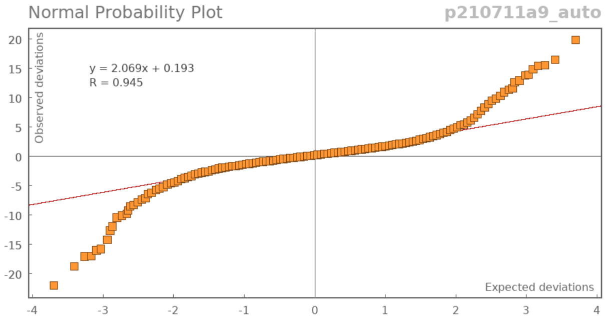

(h) VOCYEV, DiSCaMB-HAR

Figure S 39: Normal probability plots plots.

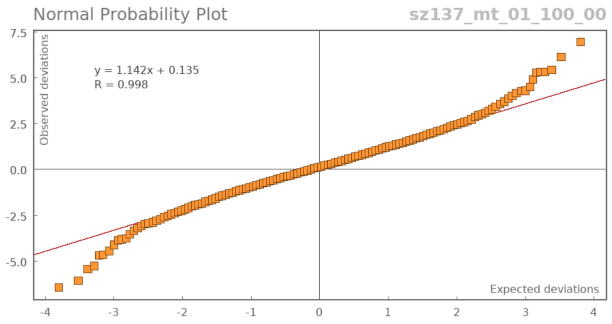

(a) SZ3-150K, IAM

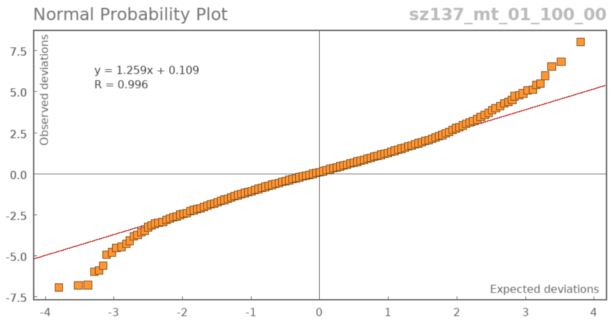

(b) SZ3-150K, DiSCaMB-HAR

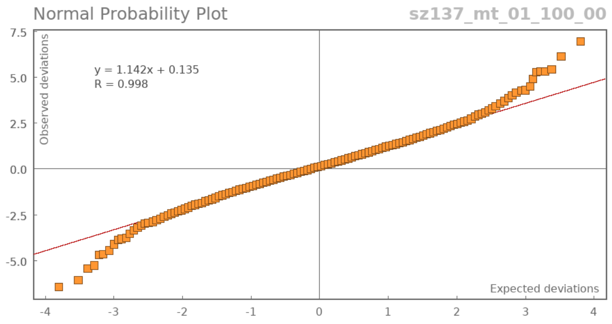

(c) SZ7-100K, IAM

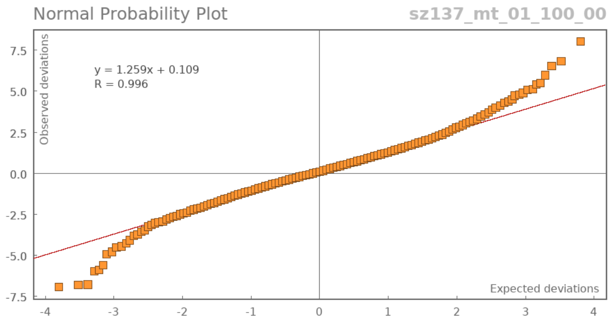

(d) SZ7-100K, DiSCaMB-HAR

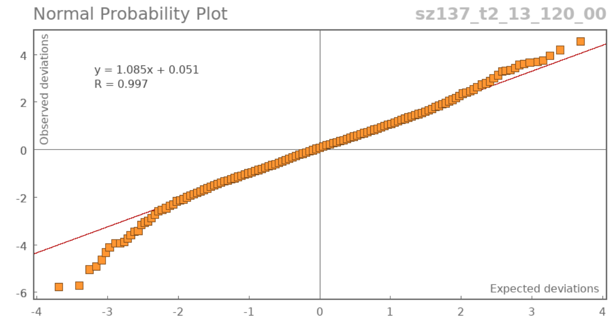

(e) SZ7-125K, IAM

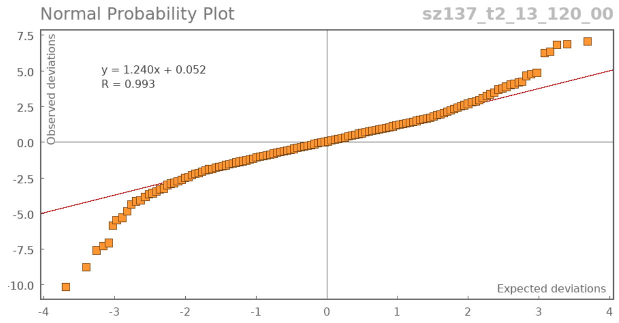

(f) SZ7-125K, DiSCaMB-HAR

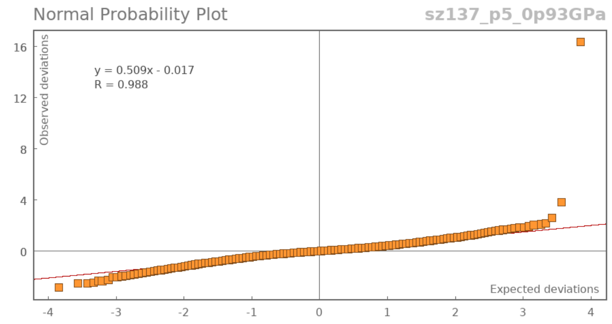

(g) SZ7-1GPa, IAM

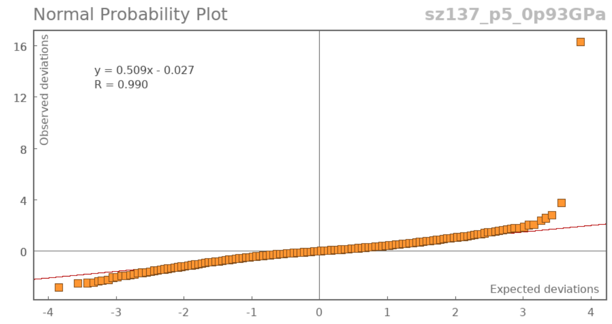

(h) SZ7-1GPa, DiSCaMB-HAR

Figure S 40: Normal probability plots plots.

Table S1 Experimental and computational details of the IAM, DiSCaMB-HAR and XHARPy-HAR crystal structures. The IAM structures were re-refined with *Olex2.refine* based on the original structures.

Identifier	Refinement type	R	wR2	GooF	$\Delta\rho_{min/max}$ [e/ Å^3]	Refined H positions	Refined H thermal motion	Chemical formula	Space group	Temperature (K)	Wavelength [Å]	d [Å]	Completeness	R_{int}	Computational details
**SZ3-150K**	IAM	3.08%	7.47%	1.023	-1.01/0.57	All	Iso, all refined	$C_{12}H_{15}CuN_3OS$	$P\ 2_1/n$	150	0.71073	0.620	99.97%	7.9 %	
	DiSCaMB-HAR	2.63 %	6.48 %	0.906	-0.75/0.64	All	Anis, all refined								B3LYP/Def2-SVP, Gaussian
	XHARPy-HAR	2.63 %	5.35 %	1.003	-0.82/0.57	All	Anis, all refined								SCAN
**SZ7-1GPa**	IAM	4.13 %	7.41 %	1.017	-0.92/0.92	All	Iso, all refined	$C_{12}H_{14}BrCuN_3OS$	$P\ ca2_1$	293(2)	0.56087	0.612	70.00%	6.03 %	
	DiSCaMB-HAR	3.93 %	6.09 %	1.015	-0.99/0.94	All	Iso, all refined								B3LYP/Def2-SVP, Gaussian
**SZ7-100K**	IAM	3.93 %	7.46 %	1.045	-0.67/0.60	All	Iso, 9 of 28	$C_{12}H_{14}BrCuN_3OS$	$P\ ca2_1$	100.00(10)	0.71073	0.720	93.58%	3.99 %	
	DiSCaMB-HAR	3.77 %	7.19 %	1.006	-0.65/0.61	All	Iso, all refined except H5B								B3LYP/Def2-SVP, Orca
	XHARPy-HAR	3.76 %	5.19 %	1.628	-0.69/0.78	All	Iso, 18 of 28 refined								SCAN
**SZ7-125K**	IAM	1.97 %	4.89 %	1.059	-0.40/0.27	All	Iso, all refined	$C_{12}H_{14}BrCuN_3OS$	$P\ ca2_1$	125.06(10)	1.54184	0.806	98.33%	2.36 %	
	DiSCaMB-HAR	1.67 %	4.15 %	0.897	-0.42/0.28	All	Iso, all refined								B3LYP/Def2-SVP, Gaussian
	XHARPy-HAR	1.67 %	3.39 %	1.334	-0.45/0.34	All	Iso, all fixed								PBE
**CUGLYM08**[1]	IAM	3.98 %	8.70 %	1.043	-0.54/0.47	All	Iso, all refined	$C_4H_{10}CuN_2O_5$	$P\ 2_12_12_1$	273(2)	0.71073	0.757	100.00%	5.33 %	
	DiSCaMB-HAR	3.75 %	8.13 %	0.974	-0.53/0.40	All	Iso, all refined								B3LYP/Def2-SVP, Orca
	XHARPy-HAR	3.76 %	6.44 %	1.132	-0.52/0.40	All	Iso, all refined								SCAN
**HOYKUD**[2]	IAM	4.2 %	12.42 %	1.043	-0.36/0.84	All	Iso, all refined	$C_{13}H_8CuN_2O_4$	$P\ 2_1/c$	150(2)	1.54178	0.845	95.60%	2.45 %	
	DiSCaMB-HAR	19 %	54.38 %	0.978	-0.99/0.82	All except H1, H7	Iso, all refined except H1, H7								B3LYP/Def2-SVP, Orca
**HACNIL**[3]	IAM	5.84 %	16.79 %	1.067	-0.38/0.57	7 of 22 refined	Iso, 5 of 22 refined	$C_{38}H_{22}N_2O_5Zn$	$I\ 4_1/a$	100(2)	0.7749	0.928	99.60%	5.51 %	
	DiSCaMB-HAR	6.08 %	23.02 %	1.128	-0.47/0.71	7 of 22 refined	Iso, 7 of 22 refined								B3LYP/Def2-SVP, Orca
**GEYQEJ**[4]	IAM	8.93 %	32.82 %	1.140	-0.45/0.67	All	Iso, all refined	$C_{54}H_{42}N_{14}Ni$	$R\ -3$	100(2)	0.71073	0.748	99.80%	13.07 %	
	DiSCaMB-HAR	8.87 %	33.32 %	1.160	-0.83/0.65	2 of 7 refined	Iso, 2 of 7 refined								B3LYP/Def2-SVP, Gaussian
**RIHYIT**[5]	IAM	8.77 %	25.26 %	1.084	-0.49/0.47	All except H1A, H1B	All except H1A, H1B	$C_{82}H_{56}N_8, 8(H_2O)$	$I\ 4_1/a$	100(2)	1.0332	0.830	99.40%	6.87 %	
	DiSCaMB-HAR	8.77 %	24.97 %	1.096	-0.53/0.52	All except H1A, H1B	All except H1A, H1B								B3LYP/Def2-SVP, Gaussian
**RIHYOZ01**[5]	IAM	5.97 %	18.79 %	0.962	-0.33/0.31	All	Iso, all refined	$C_{41}H_{28}N_4$	$I\ 4_1/a$	99.9(3)	0.71073	0.850	94.80%	18.57 %	
	DiSCaMB-HAR	6.2 %	18.8 %	0.962	-0.33/0.32	All	Iso, all refined								B3LYP/Def2-SVP, Gaussian
**VUSKEA01**[6]	IAM	5.95 %	35.89 %	1.471	-0.91/1.78	All	Iso, all refined	$C_{24}H_{12}O_{13}Zn_4$	$F\ m\ -3\ m$	120	0.71073	0.770	99.90%	17.51 %	
	DiSCaMB-HAR	6.02 %	36.11 %	1.478	-0.87/1.79	All	Iso, all refined								B3LYP/cc-pVDZ, Gaussian
**QOXVEH**[7]	IAM	6.71 %	21.72 %	1.011	-0.25/0.86	16 of 24 refined	10 of 24 refined	$C_{28}H_{18}Al_2O_{10}$, $0.58(C_3H_7NO)$	$P\ 4_22_12$	100	0.7288	0.780	96.60%	5.79 %	
	DiSCaMB-HAR	6.54 %	21.22 %	1.028	-0.25/0.92	16 of 24 refined	10 of 24 refined								B3LYP/cc-pVDZ, Gaussian

Table S2 Experimental and computational details of the IAM, DiSCaMB-HAR and XHARPy-HAR crystal structures. The IAM structures were re-refined with *Olex2.refine* based on the original structures.

Identifier	Refinement type	R	wR2	GooF	$\Delta\rho_{min/max}$ [e/Å^3]	Refined H positions	Refined H thermal motion	Chemical formula	Space group	Temperature (K)	Wavelength [Å]	d [Å]	Completeness	R_{int}	Computational details
**ALUDOD**[8]	IAM	7.61 %	24.46 %	1.011	-0.62/1.31	All	Iso, all refined	$C_{54}H_{42}N_{14}Ni$	R -3	150(2)	0.71073	0.748	99.20%	12.25 %	
	DiSCaMB-HAR	7.39 %	24.12 %	1.011	-0.72/1.37	All	Iso, all except H2								B3LYP/cc-pVDZ, Gaussian
**FUVKEP**[9]	IAM	8.05 %	25.47 %	1.050	-1.63/2.15	All	Iso, all except H2	$C_{36}H_{27}Co_3N_4O_9$	P 6_3/mcm	100	0.71073	0.800	99.90%	8.96 %	
	DiSCaMB-HAR	8.11 %	26.22 %	1.038	-1.57/2.20	All	Iso, all except H2								B3LYP/cc-pVDZ, Gaussian
**UYETIE**[10]	IAM	7.6 %	32.08 %	1.138	-0.82/0.50	All	Iso, all refined	$C_{66}H_{48}N_{12}Zn$	R -3	150(2)	0.71073	0.796	99.00%	4.94 %	
	DiSCaMB-HAR	7.54 %	32.28 %	1.146	-0.77/0.44	All	Iso, all refined								B3LYP/cc-pVDZ, Gaussian
**CAMTET**[11]	IAM	5.54 %	15.46 %	1.022	-0.50/0.86	All	Iso, 3 of 6 refined	$C_5H_3AlN_2O_5$	P 2_1/c	100	0.7288	0.839	99.10%	8.38 %	
	DiSCaMB-HAR	5.37 %	14.87 %	1.027	-0.62/0.98	All except H1A refined	Iso, 3 of 6 refined								B3LYP/cc-pVDZ, Gaussian
**CAMTET01**[11]	IAM	4.82 %	13.22 %	0.990	-0.41/0.71	All	Iso, all except H3	$C_5H_3AlN_2O_5$	P 2_1/c	330	0.7288	0.839	99.00%	7.49 %	
	DiSCaMB-HAR	4.63 %	12.74 %	0.996	-0.40/0.73	All	Iso, all except H3								B3LYP/cc-pVDZ, Gaussian
**RALFIX**[12]	IAM	5.55 %	15.67 %	1.041	-0.57/0.49	All	Iso, all refined	$C_{22}H_{13}CoN_2O_5$	R -3	273.15	0.71073	0.841	99.80%	10.94 %	
	DiSCaMB-HAR	5.42 %	15.25 %	1.061	-0.55/0.57	All	3 anis, 8 iso								B3LYP/cc-pVDZ, Gaussian
**PODWIS**[13]	IAM	7.82 %	26.55 %	1.018	-2.91/3.88	3 of 7 refined	Iso, not refined	$C_8H_7Ni_2O_6$	R -3	300	0.4859	0.508	79.30%	4.99 %	
	DiSCaMB-HAR	7.8 %	26.7 %	1.017	-2.96/3.85	1 of 7 refined	Iso, not refined								B3LYP/cc-pVDZ, Gaussian
**VOCYEV**[14]	IAM	4.4 %	12.12 %	1.064	-0.99/1.07	All	Iso, all refined	$C_8H_7Ni_2O_6$	R -3	120(2)	0.71073	0.728	84.80%	5.07 %	
	DiSCaMB-HAR	4.08 %	11.3 %	0.992	-0.91/1.03	All	Iso, all refined								B3LYP/cc-pVDZ, Gaussian

Table S3 Statistics calculated for X-H bond lengths the 20 single-crystal X-ray structures for three refinement types (IAM, DiSCaMB-HAR and XHARPy-HAR). Structures are ordered according to the DiSCaMB-HAR data-refinement quality ranking. MD stands for mean difference and MAD stands for mean absolute difference between the experimental X-H bond lengths and the values resulting from geometry optimization. Mean σ stands for bond length standard deviation averaged for all X-H bond lengths in given structure. *Difference statistically insignificant.

**Identifier**	**IAM**			**DiSCaMB-HAR**			**XHARPy-HAR**		
	**MD [Å]**	**MAD [Å]**	**Mean σ [Å]**	**MD [Å]**	**MAD [Å]**	**Mean σ [Å]**	**MD [Å]**	**MAD [Å]**	**Mean σ [Å]**
**SZ7-125K**	-0.161	0.161	0.040	-0.005*	0.025	0.039	-0.007*	0.030	0.035
**CUGLYM08**	-0.224	0.224	0.054	-0.071*	0.039	0.064	-0.021*	0.043	0.059
**SZ7-100K**	-0.138	0.142	0.058	0.009*	0.049	0.063	0.004*	0.034	0.051
**SZ3-150K**	-0.170	0.170	0.029	-0.017	0.019	0.026	-0.016	0.019	0.025
**VOCYEV**	-0.163	0.166	0.043	-0.005	0.046	0.043			
**CAMTET01**	0.024	0.233	0.033	-0.029	0.136	0.043			
**SZ7-1GPa**	-0.150	0.158	0.048	-0.005*	0.051	0.051			
**HOYKUD**	-0.128	0.133	0.043	-0.010	0.074	0.030			
**HACNIL**	-0.140	0.140	0.050	-0.086	0.116	0.066			
**RALFIX**	-0.121	0.126	0.042	0.018	0.056	0.045			
**RIHYIT**	-0.110	0.110	0.043	-0.038	0.070	0.049			
**UYETIE**	-0.063	0.129	0.053	0.101	0.169	0.065			
**QOXVEH**	-0.094	0.133	0.039	-0.132	0.165	0.035			
**CAMTET**	-0.060	0.254	0.053	0.046	0.198	0.056			
**PODWIS**	-0.132	0.132	0.090	-0.133	0.133	0.070			
**ALUDOD**	-0.113	0.115	0.041	0.025	0.078	0.050			
**FUVKEP**	0.083	0.259	0.072	0.081	0.123	0.156			
**RIHYOZ**	-0.121	0.131	0.061	-0.010	0.071	0.053			
**VUSKEA01**	-0.060	0.060	0.140	-0.090	0.090	0.040			
**GEYKEJ**	0.013	0.128	0.070	-0.067	0.176	0.021			

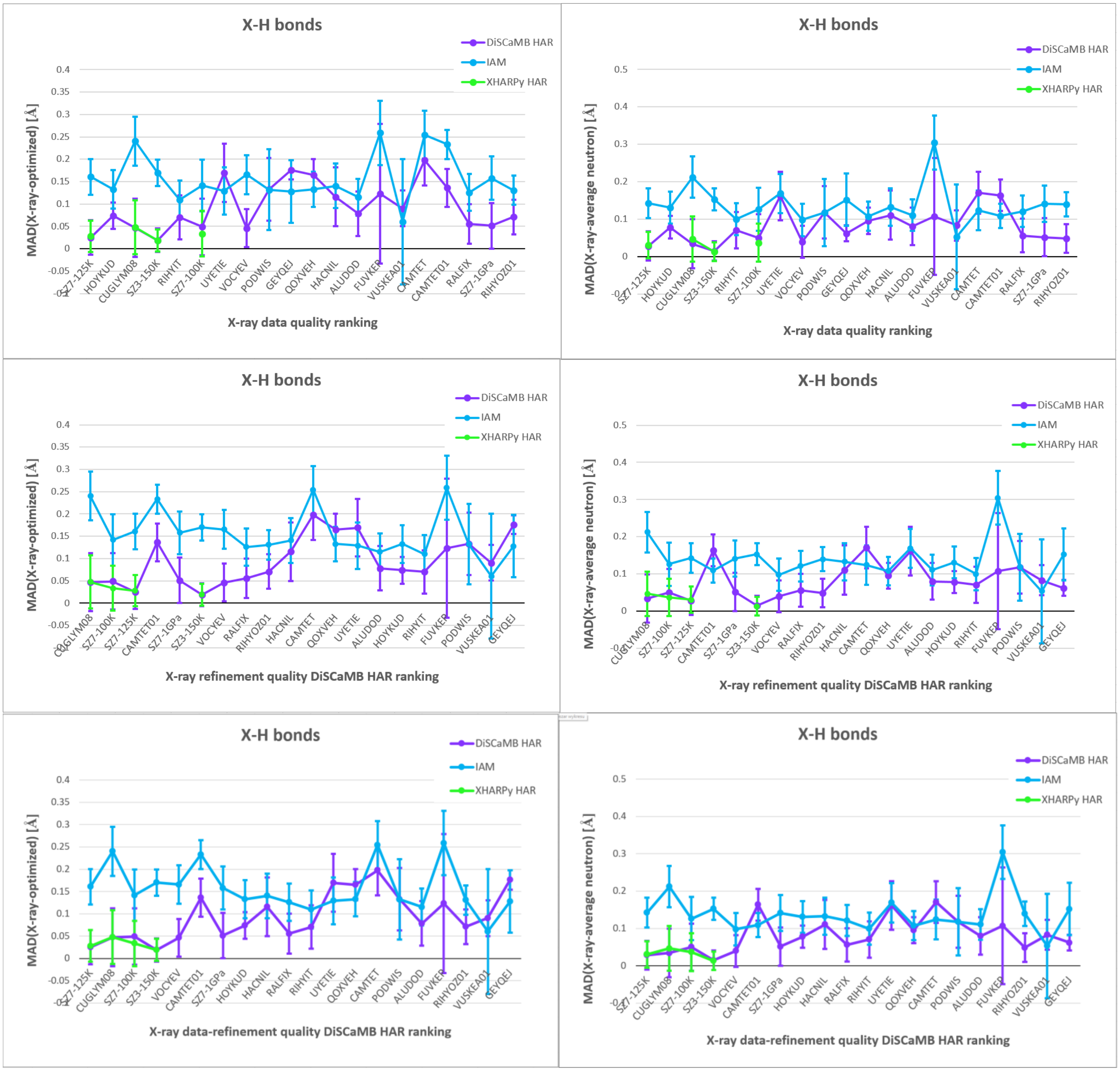

Figure S41 Mean absolute difference between the optimized (left) or average neutron bond lengths (right) and the experimental bond lengths obtained with three refinement types (IAM, DiSCaMB-HAR and XHARPy-HAR) against X-ray data for 20 polymeric structures arranged from the best to the worst according to data, DiSCaMB-HAR refinement and DiSCaMB-HAR data-refinement quality ranking.

Data and refinement quality ranking

The data sets were ranked according to the quality of experimental data and the quality of the refined model. The ranking of structures by data quality was based on data completeness, R_{int} and data resolution. Structures were ordered from the "best" to the "worst" according to given quantity. The place in the ranking became the number of points, which were summed for each structure in order to determine the position of the structure in the final ranking (Table S3). Similarly, quality of the refinement was evaluated using statistical values such as goodness of fit, R, wR2 and the range of residual density values (Δρ range). This ranking was done for IAM (Table S4), DiSCaMB-HAR (Table S5) and XHARPy-HAR (Table S6). The values of all the parameters taken into account are given in Table S1 and S2. At the end, the points obtained in the data quality and the refinement quality ranking were summed for each structure in order to obtain the final data-refinement ranking of IAM, DiSCaMB-HAR and XHARPy-HAR structures (Table S7). The obtained rankings are only tentative since the differences between parameters are in certain cases very small and all the parameters were considered with equal importance, which might not be optimal.

Table S4 Data quality ranking of the structures. The number of points for each structure is given on the left. Structures are ordered from the best to the worst.

Completeness		R_{int}		Resolution		Overall data quality	
1	CUGLYM08	1	SZ7-125K	1	SZ7-125K	12	SZ7-125K
2	SZ3-150K	2	HOYKUD	2	HOYKUD	16	HOYKUD
3	VUSKEA01	3	SZ7-100K	3	RIHYIT	18	CUGLYM08
3	FUVKEP	4	UYETIE	4	SZ3-150K	19	SZ3-150K
4	GEYQEJ	5	PODWIS	5	SZ7-100K	20	RIHYIT
4	RALFIX	6	VOCYEV	6	VOCYEV	22	SZ7-100K
5	HACNIL	7	CUGLYM08	7	PODWIS	27	UYETIE
6	RIHYIT	8	HACNIL	8	GEYQEJ	27	VOCYEV
7	ALUDOD	9	QOXVEH	9	ALUDOD	28	PODWIS
8	CAMTET	10	SZ7-1GPa	10	CUGLYM08	30	GEYQEJ
9	UYETIE	11	RIHYIT	11	QOXVEH	31	QOXVEH
9	CAMTET01	12	CAMTET01	12	VUSKEA01	33	HACNIL
10	SZ7-125K	13	SZ3-150K	13	SZ7-1GPa	33	ALUDOD
11	QOXVEH	14	CAMTET	14	UYETIE	33	FUVKEP
12	HOYKUD	15	FUVKEP	15	FUVKEP	34	VUSKEA01
13	RIHYOZ01	16	RALFIX	16	CAMTET	38	CAMTET
14	SZ7-100K	17	ALUDOD	17	CAMTET01	38	CAMTET01
15	VOCYEV	18	GEYQEJ	18	RALFIX	38	RALFIX
16	PODWIS	19	VUSKEA01	19	RIHYOZ01	40	SZ7-1GPa
17	SZ7-1GPa	20	RIHYOZ01	20	HACNIL	52	RIHYOZ01

Table S5 Refinement quality ranking of the structures refined with IAM. The number of points for each structure is given on the left. Structures are ordered from the best to the worst.

GooF		R		wR2		$\Delta\rho_{min/max}$		Overall refinement quality	
1	QOXVEH	1	SZ7-125K	1	SZ7-125K	1	RIHYOZ01	19	SZ7-125K
1	ALUDOD	2	SZ3-150K	2	SZ3-150K	2	SZ7-125K	20	SZ7-100K
1	CAMTET01	3	SZ7-100K	3	SZ7-1GPa	3	HACNIL	22	CUGLYM08
2	SZ7-1GPa	4	CUGLYM08	4	SZ7-100K	4	RIHYIT	29	PODWIS
3	PODWIS	5	SZ7-1GPa	5	CUGLYM08	5	CUGLYM08	29	SZ7-1GPa
4	CAMTET	6	HOYKUD	6	VOCYEV	6	RALFIX	31	RALFIX
5	SZ3-150K	7	VOCYEV	7	HOYKUD	7	QOXVEH	32	HOYKUD
6	RIHYOZ01	8	CAMTET01	8	CAMTET01	8	CAMTET01	32	CAMTET01
7	RALFIX	9	CAMTET	9	CAMTET	9	GEYQEJ	35	RIHYOZ01
8	CUGLYM08	10	RALFIX	10	RALFIX	10	HOYKUD	38	ALUDOD
9	HOYKUD	11	HACNIL	11	HACNIL	11	SZ7-100K	39	HACNIL
10	SZ7-100K	12	VUSKEA01	12	RIHYOZ01	12	UYETIE	43	SZ3-150K
11	FUVKEP	13	RIHYOZ01	13	QOXVEH	13	CAMTET	46	QOXVEH
12	SZ7-125K	14	QOXVEH	14	ALUDOD	14	SZ3-150K	49	CAMTET
13	VOCYEV	15	UYETIE	15	RIHYIT	15	SZ7-1GPa	51	VUSKEA01
14	HACNIL	16	ALUDOD	16	FUVKEP	16	ALUDOD	58	RIHYIT
15	RIHYIT	17	PODWIS	17	PODWIS	17	VOCYEV	59	GEYQEJ
16	UYETIE	18	FUVKEP	18	UYETIE	18	VUSKEA01	61	FUVKEP
17	GEYQEJ	19	RIHYIT	19	GEYQEJ	19	FUVKEP	67	VOCYEV
18	VUSKEA01	20	GEYQEJ	20	VUSKEA01	20	PODWIS	68	UYETIE

Table S6 Refinement quality ranking of the structures refined with DiSCaMB-HAR. The number of points for each structure is given on the left. Structures are ordered from the best to the worst.

	GooF		R		wR2		$\Delta\rho_{min/max}$		Overall refinement quality
1	CAMTET01	1	SZ7-125K	1	SZ7-125K	1	RIHYOZ01	18	CUGLYM08
2	SZ7-100K	2	SZ3-150K	2	SZ7-1GPa	2	SZ7-125K	20	SZ7-100K
3	VOCYEV	3	CUGLYM08	3	SZ3-150K	3	CUGLYM08	20	SZ7-125K
4	ALUDOD	4	SZ7-100K	4	SZ7-100K	4	RIHYIT	21	CAMTET01
5	SZ7-1GPa	5	SZ7-1GPa	5	CUGLYM08	5	RALFIX	27	SZ7-1GPa
6	PODWIS	6	VOCYEV	6	VOCYEV	6	CAMTET01	30	SZ3-150K
7	HOYKUD	7	CAMTET01	7	CAMTET01	7	QOXVEH	31	VOCYEV
8	CUGLYM08	8	CAMTET	8	CAMTET	8	HACNIL	36	RALFIX
9	CAMTET	9	RALFIX	9	RALFIX	9	UYETIE	38	RIHYOZ01
10	QOXVEH	10	VUSKEA01	10	RIHYOZ01	10	SZ7-100K	39	HACNIL
11	FUVKEP	11	HACNIL	11	QOXVEH	11	SZ3-150K	47	CAMTET
12	RIHYOZ01	12	RIHYOZ01	12	HACNIL	12	GEYQEJ	51	QOXVEH
13	RALFIX	13	QOXVEH	13	ALUDOD	13	CAMTET	52	UYETIE
14	SZ3-150K	14	ALUDOD	14	RIHYIT	14	HOYKUD	54	HOYKUD
15	RIHYIT	15	UYETIE	15	FUVKEP	15	SZ7-1GPa	54	ALUDOD
16	SZ7-125K	16	PODWIS	16	PODWIS	16	VOCYEV	55	RIHYIT
17	HACNIL	17	FUVKEP	17	UYETIE	17	ALUDOD	55	FUVKEP
18	UYETIE	18	RIHYIT	18	GEYQEJ	18	VUSKEA01	58	PODWIS
19	GEYQEJ	19	GEYQEJ	19	VUSKEA01	19	FUVKEP	59	VUSKEA01
20	VUSKEA01	20	HOYKUD	20	HOYKUD	20	PODWIS	66	GEYQEJ

Table S7 Refinement quality ranking of the structures refined with XHARPy-HAR. The number of points for each structure is given on the left. Structures are ordered from the best to the worst.

	GooF		R		wR2		$\Delta\rho_{min/max}$		Overall refinement quality
1	SZ3-150K	1	SZ7-125K	1	SZ7-125K	1	SZ7-125K	6	SZ7-125K
2	CUGLYM08	2	SZ3-150K	2	SZ7-100K	2	CUGLYM08	9	SZ3-150K
3	SZ7-125K	3	CUGLYM08	3	SZ3-150K	3	SZ3-150K	11	CUGLYM08
4	SZ7-100K	3	SZ7-100K	4	CUGLYM08	4	SZ7-100K	13	SZ7-100K

Table S8 Data and refinement quality ranking of the structures refined with IAM, DiSCaMB-HAR and XHARPy-HAR. The number of points for each structure is given on the left. Structures are ordered from the best to the worst.

IAM		DiSCaMB-HAR		XHARPy-HAR	
31	SZ7-125K	32	SZ7-125K	18	SZ7-125K
40	CUGLYM08	36	CUGLYM08	28	SZ3-150K
42	SZ7-100K	42	SZ7-100K	29	CUGLYM08
48	HOYKUD	49	SZ3-150K	35	SZ7-100K
57	PODWIS	58	VOCYEV		
62	SZ3-150K	59	CAMTET01		
69	RALFIX	67	SZ7-1GPa		
69	SZ7-1GPa	70	HOYKUD		
70	CAMTET01	72	HACNIL		
71	ALUDOD	74	RALFIX		
72	HACNIL	75	RIHYIT		
77	QOXVEH	79	UYETIE		
78	RIHYIT	82	QOXVEH		
85	VUSKEA01	85	CAMTET		
87	RIHYOZ01	86	PODWIS		
87	CAMTET	87	ALUDOD		
89	GEYQEJ	88	FUVKEP		
94	FUVKEP	90	RIHYOZ01		
94	VOCYEV	93	VUSKEA01		
95	UYETIE	96	GEYQEJ		

Experimental details for the SZ3-150K, SZ7-100K, SZ7-125K and SZ7-1GPa compounds.

Crystallization

The crystals were obtained by slow evaporation of the solvent from a solution in acetonitrile.

Sample preparation for high-pressure experiments

Selected crystal specimen (AM1GPa) was placed in a Diamond Anvil Cell (DAC) of a modified Merrill and Bassett design[15] of an effective opening angle of 52°, using n-pentane/isopentane as pressure-transferring medium. The crystal was oriented on the diamond culet using epoxy glue to improve data completeness[16]. A steel gasket of 260 μm thickness with a 350 μm diameter opening was prepared for the experiment. The pressure within the gasket hole was estimated by the ruby fluorescence method, using pRuby package[17], by fitting a Gaussian curve to the fluorescence spectrum and using IPPS ruby gauge[18] and temperature correction[19].

Single-crystal X-ray Diffraction

X-ray diffraction (XRD) data were collected using three different Rigaku Oxford Diffraction SuperNova diffractometers equipped with an Eos CCD detector and either copper (Cu Kα, λ = 1.54184 Å), molybdenum (Mo Kα, λ = 0.71073 Å), or silver (Ag Kα, λ = 0.56087 Å) microsource. Ambient pressure samples were mounted on Mitegen loops using a trace amount of Paratone-N oil and cooled to temperatures of 100 K, 125 K, and 150 K using an Oxford Cryosystems cooler. The CrysAlisPro program was used for data collection and reduction. Absorption correction based on the crystal shape was applied using the same software, employing **SCALE3 ABSPACK** as well as **Absorb**[20,21], except for sample Sz150K, for which a multi-scan absorption correction via SCALE3 ABSPACK was used without considering the crystal shape.

Crystal structure solution

Crystal structures were solved by SHELXS[22] and then refined by SHELSXL[23] within Olex2 graphical user interface[24]. In the case of SZ3-150K, data was cut from the resolution of 2θ = 128.5º to the resolution of 2θ = 70º so that all hydrogen ADPs could be refined with HAR.

Table S9 Data reduction and refinement details for the SZ3-150K, SZ7-100K, SZ7-125K and SZ7-1GPa compounds.

Identifier	AM100K	AM125K	AM1GPa	Sz150K
**Crystal data**				
Sum Formula, M_r	$C_{12}H_{14}BrCuN_3OS$, 391.77			$C_{12}H_{15}CuN_3OS$, 312.87
Symmetry information	Orthorhombic, $Pca2_1$, Z = 8, Z` = 2			Monoclinic, $P2_1/n$, Z = 4, Z` = 1
Temperature [K]	100.00(10)	125.06(10)	293(2)	150
Pressure [GPa]	0.00	0.00	0.93	0.00
Opening Angle [°]	-	-	52	-
a [Å]	11.6813(4)	11.69284(15)	11.6130(7)	6.98066(9)
b [Å]	7.3182(2)	7.32217(10)	7.2186(3)	18.8024(3)
c [Å]	33.7593(10)	33.7855(4)	33.071(3)	10.12710(16)
β [°]	90	90	90	97.3016(14)
V [Å³]	2885.94(16)	2892.61(7)	2772.3(3)	1318.43(3)
Crystal Size [mm³]	0.18 x 0.11 x 0.06	0.45 x 0.11 x 0.08	0.18 x 0.15 x 0.1	0.37 x 0.22 x 0.08
Density [g/cm³]	1.803	1.799	1.877	1.576
**Data collection**				
Radiation type	micro-focus sealed X-ray tube			
Radiation Source	Mo Kα	Cu Kα	Ag Kα	Mo Kα
Wavelength [Å]	0.71073	1.54184	0.56087	0.71073
Absorption correction	Gaussian			Multi-scan
μ [mm⁻¹]	4.421	6.679	2.438	1.805
Tmin	0.589	0.190	0.446	0.42561
Tmax	0.981	1.000	0.555	1
Diffractometer	Rigaku Oxford Diffraction SuperNova			
Resolution [Å]	0.7197	0.8064	0.6124	0.6189
Reflections: measured	25476	12063	38252	34131
independent	6950	4393	8147	5825
observed	6429	4355	5143	5194
h range	[-15, 13]	[-14, 14]	[-16, 17]	[-11, 11]
k range	[8, -9]	[-8, 8]	[-10, 11]	[-29, 30]
l range	[-45, 46]	[-41, 39]	[-42, 41]	[-16, 16]
Rint	0.0399	0.0236	0.0603	0.0544
Rsigma	0.044	0.026	0.106	0.0484
Completeness	1.000	0.996	0.864	0.999
**Refinement**				
R1	0.0442	0.0241	0.0968	0.036
R1 (I > 2σ(I))	0.0388	0.0239	0.0416	0.0308
wR2	0.0761	0.0619	0.0768	0.0728
wR2 (I > 2σ(I))	0.0737	0.0617	0.0669	0.0695
GooF	1.056	1.044	1.01	1.044
Restrains	1			0
Parameters	347			165
Δρmax [e/Å³]	0.618	0.330	0.425	0.569
Δρmin [e/Å³]	-0.671	-0.426	-0.418	-1.011
Δρrms [e/Å³]	0.105	0.060	0.090	0.078

References

(1) Sattar, T.; Athar, M. Hydrothermal Synthesis and Characterization of Copper Glycinate (Bio-MOF-29) and Its in Vitro Drugs Adsorption Studies. *Open Journal of Inorganic Chemistry* **2017**, *7* (2), 17–27. https://doi.org/10.4236/ojic.2017.72002.

(2) Chen, Z.; Adil, K.; Weseliński, Ł. J.; Belmabkhout, Y.; Eddaoudi, M. A Supermolecular Building Layer Approach for Gas Separation and Storage Applications: The Eea and Rtl MOF Platforms for CO2 Capture and Hydrocarbon Separation. *J. Mater. Chem. A* **2015**, *3* (12), 6276–6281. https://doi.org/10.1039/C4TA07115H.

(3) Rudd, N. D.; Wang, H.; Fuentes-Fernandez, E. M. A.; Teat, S. J.; Chen, F.; Hall, G.; Chabal, Y. J.; Li, J. Highly Efficient Luminescent Metal–Organic Framework for the Simultaneous Detection and Removal of Heavy Metals from Water. *ACS Appl. Mater. Interfaces* **2016**, *8* (44), 30294–30303. https://doi.org/10.1021/acsami.6b10890.

(4) Desai, A. V.; Roy, A.; Samanta, P.; Manna, B.; Ghosh, S. K. Base-Resistant Ionic Metal-Organic Framework as a Porous Ion-Exchange Sorbent. *iScience* **2018**, *3*, 21–30. https://doi.org/10.1016/j.isci.2018.04.004.

(5) Ma, T.; Kapustin, E. A.; Yin, S. X.; Liang, L.; Zhou, Z.; Niu, J.; Li, L.-H.; Wang, Y.; Su, J.; Li, J.; Wang, X.; Wang, W. D.; Wang, W.; Sun, J.; Yaghi, O. M. Single-Crystal x-Ray Diffraction Structures of Covalent Organic Frameworks. *Science* **2018**, *361* (6397), 48–52. https://doi.org/10.1126/science.aat7679.

(6) Denisov, G. L.; Primakov, P. V.; Korlyukov, A. A.; Novikov, V. V.; Nelyubina, Yu. V. Solvothermal Synthesis of the Metal-Organic Framework MOF-5 in Autoclaves Prepared by 3D Printing. *Russ J Coord Chem* **2019**, *45* (12), 836–842. https://doi.org/10.1134/S1070328419120030.

(7) Pei, X.; Bürgi, H.-B.; Kapustin, E. A.; Liu, Y.; Yaghi, O. M. Coordinative Alignment in the Pores of MOFs for the Structural Determination of N-, S-, and P-Containing Organic Compounds Including Complex Chiral Molecules. *J. Am. Chem. Soc.* **2019**, *141* (47), 18862–18869. https://doi.org/10.1021/jacs.9b10501.

(8) Sharma, S.; Let, S.; Desai, A. V.; Dutta, S.; Karuppasamy, G.; Shirolkar, M. M.; Babarao, R.; Ghosh, S. K. Rapid, Selective Capture of Toxic Oxo-Anions of Se(IV), Se(VI) and As(V) from Water by an Ionic Metal–Organic Framework (iMOF). *J. Mater. Chem. A* **2021**, *9* (10), 6499–6507. https://doi.org/10.1039/D0TA04898D.

(9) Zhang, X.; Zhang, Y.-Z.; Jin, Y.-Q.; Geng, L.; Zhang, D.-S.; Hu, H.; Li, T.; Wang, B.; Li, J.-R. Pillar-Layered Metal–Organic Frameworks Based on a Hexaprismane [Co6(M3-OH)6] Cluster: Structural Modulation and Catalytic Performance in Aerobic Oxidation Reaction. *Inorg. Chem.* **2020**, *59* (16), 11728–11735. https://doi.org/10.1021/acs.inorgchem.0c01611.

(10) Dutta, S.; Let, S.; Shirolkar, M. M.; Desai, A. V.; Samanta, P.; Fajal, S.; More, Y. D.; Ghosh, S. K. A Luminescent Cationic MOF for Bimodal Recognition of Chromium and Arsenic Based Oxo-Anions in Water. *Dalton Trans.* **2021**, *50* (29), 10133–10141. https://doi.org/10.1039/D1DT01097B.

(11) *Evolution of water structures in metal-organic frameworks for improved atmospheric water harvesting | Science*. https://www.science.org/doi/10.1126/science.abj0890 (accessed 2025-04-28).

(12) Liu, Q.; Zhang, L.-Y.; Bao, Y.-M.; Zhang, N.; Zhang, J.-Y.; Xing, Y.-Y.; Deng, W.; Liu, Z.-J. Structures and Catalytic Oxidative Coupling Reaction of Four Co-MOFs Modified with R-Isophthalic Acid (RH, OH and COOH) and Trigonal Ligands. *CrystEngComm* **2021**, *23* (43), 7590–7601. https://doi.org/10.1039/D1CE01221E.

(13) Main, R. M.; Vornholt, S. M.; Ettlinger, R.; Netzsch, P.; Stanzione, M. G.; Rice, C. M.; Elliott, C.; Russell, S. E.; Warren, M. R.; Ashbrook, S. E.; Morris, R. E. In Situ Single-Crystal X-Ray Diffraction Studies of Physisorption and Chemisorption of SO2 within a Metal–Organic Framework and Its Competitive Adsorption with Water. *J. Am. Chem. Soc.* **2024**, *146* (5), 3270–3278. https://doi.org/10.1021/jacs.3c11847.

(14) Guo, P.-F.; Liang, Z.-L.; Jiao, Y.-E.; Xu, H.; Zhao, B. Highly Selective and Eco-Friendly Dihydroisoquinoline Synthesis via Cu/Co Synergistic Catalysis in Cu NPs@MOFs Catalyst under Mild Conditions. *Sci. China Chem.* **2024**, *67* (5), 1561–1568. https://doi.org/10.1007/s11426-024-1997-2.

(15) Merrill, L.; Bassett, W. A. Miniature Diamond Anvil Pressure Cell for Single Crystal X-ray Diffraction Studies. *Review of Scientific Instruments* **1974**, *45* (2), 290–294. https://doi.org/10.1063/1.1686607.

(16) Tchoń, D.; Makal, A. Maximizing Completeness in Single-Crystal High-Pressure Diffraction Experiments: Phase Transitions in 2°AP. *IUCrJ* **2021**, *8* (6), 1006–1017. https://doi.org/10.1107/S2052252521009532.
(17) Tchoń, D. Baharis/pRuby, 2024. https://github.com/Baharis/pRuby (accessed 2025-04-28).
(18) Shen, G.; Wang, Y.; Dewaele, A.; Wu, C.; Fratanduono, D. E.; Eggert, J.; Klotz, S.; Dziubek, K. F.; Loubeyre, P.; Fat'yanov, O. V.; Asimow, P. D.; Mashimo, T.; Wentzcovitch, R. M. M. Toward an International Practical Pressure Scale: A Proposal for an IPPS Ruby Gauge (IPPS-Ruby2020). *High Pressure Research* **2020**, *40* (3), 299–314. https://doi.org/10.1080/08957959.2020.1791107.
(19) Ragan, D. D.; Gustavsen, R.; Schiferl, D. Calibration of the Ruby R1 and R2 Fluorescence Shifts as a Function of Temperature from 0 to 600 K. *Journal of Applied Physics* **1992**, *72* (12), 5539–5544. https://doi.org/10.1063/1.351951.
(20) Angel, R. J. Absorption Corrections for Diamond-Anvil Pressure Cells Implemented in the Software Package Absorb6.0. *J Appl Cryst* **2004**, *37* (3), 486–492. https://doi.org/10.1107/S0021889804005229.
(21) Burnham, C. W. Computation of Absorption Corrections, and the Significance of End Effect. *American Mineralogist* **1966**, *51* (1–2), 159–167.
(22) Sheldrick, G. M. A Short History of SHELX. *Acta Cryst A* **2008**, *64* (1), 112–122. https://doi.org/10.1107/S0108767307043930.
(23) Sheldrick, G. M. SHELXT – Integrated Space-Group and Crystal-Structure Determination. *Acta Cryst A* **2015**, *71* (1), 3–8. https://doi.org/10.1107/S2053273314026370.
(24) Dolomanov, O. V.; Bourhis, L. J.; Gildea, R. J.; Howard, J. a. K.; Puschmann, H. OLEX2: A Complete Structure Solution, Refinement and Analysis Program. *J Appl Cryst* **2009**, *42* (2), 339–341. https://doi.org/10.1107/S0021889808042726.
